# Correlation between white matter microstructure and executive functions suggests early developmental influence on long fibre tracts in preterm born adolescents

**DOI:** 10.1371/journal.pone.0178893

**Published:** 2017-06-08

**Authors:** Brigitte Vollmer, Aiko Lundequist, Gustaf Mårtensson, Zoltan Nagy, Hugo Lagercrantz, Ann-Charlotte Smedler, Hans Forssberg

**Affiliations:** 1 Neuropaediatrics, Department of Women’s and Children’s Health, Karolinska Institutet, Astrid Lindgren Children’s Hospital, Stockholm, Sweden; 2 Clinical Neurosciences, Clinical and Experimental Sciences, Faculty of Medicine, University of Southampton, Southampton, United Kingdom; 3 Department of Economics, University of Zürich, Zürich, Switzerland; 4 Neonatal Research Unit, Department of Women’s and Children’s Health, Karolinska Institutet, Astrid Lindgren Children’s Hospital, Stockholm, Sweden; 5 Department of Psychology, University of Stockholm, Stockholm, Sweden; Hopital Robert Debre, FRANCE

## Abstract

**Main objectives:**

Executive functions are frequently a weakness in children born preterm. We examined associations of executive functions and general cognitive abilities with brain structure in preterm born adolescents who were born with appropriate weight for gestational age and who have no radiological signs of preterm brain injury on neuroimaging.

**Methods:**

The Stockholm Neonatal Project (SNP) is a longitudinal, population-based study of children born preterm (<36 weeks of gestation) with very low birth weight (<1501g) between 1988–1993. At age 18 years (mean 18 years, SD 2 weeks) 134 preterm born and 94 full term participants underwent psychological assessment (general intelligence, executive function measures). Of these, 71 preterm and 63 full term participants underwent Magnetic Resonance Imaging (MRI) at mean 15.2 years (range 12–18 years), including 3D T1-weighted images for volumetric analyses and Diffusion Tensor Imaging (DTI) for assessment of white matter microstructure. Group comparisons of regional grey and white matter volumes and fractional anisotropy (FA, as a measure of white matter microstructure) and, within each group, correlation analyses of cognitive measures with MRI metrics were carried out.

**Results:**

Significant differences in grey and white matter regional volumes and widespread differences in FA were seen between the two groups. No significant correlations were found between cognitive measures and brain volumes in any group after correction for multiple comparisons. However, there were significant correlations between FA in projection fibres and long association fibres, linking frontal, temporal, parietal, and occipital lobes, and measures of executive function and general cognitive abilities in the preterm born adolescents, but not in the term born adolescents.

**Overall significance of the study:**

In persons born preterm, in the absence of perinatal brain injury on visual inspection of MRI, widespread alterations in regional brain tissue volumes and microstructure are present in adolescence/young adulthood. Importantly, these alterations in WM tracts are correlated with measures of executive function and general cognitive abilities. Our findings suggest that disturbance of neural pathways, rather than changes in regional brain volumes, are involved in the impaired cognitive functions.

## Introduction

It is well known that preterm birth is associated with adverse developmental and cognitive outcomes [1] even in the absence of neuromotor impairment, and there is evidence that difficulties persist into adolescence and young adulthood (e.g [2–6]). Based on a comprehensive review of the literature, Howard and colleagues [7] concluded that preterm born children, including low-risk children, are at risk for specific impairment in most areas of executive functioning such as working memory, cognitive flexibility and sustained attention. We recently reported on the cognitive outcome at age 18 years of our population-based cohort (the Stockholm Neonatal Project, SNP) of preterm born adolescents and their matched full term controls [8]. At odds with some previous reports, in our cohort preterm participants born after 28 weeks of gestation with appropriate birth weight and no perinatal complications performed on a par with term born controls. By contrast, preterm participants born before gestational week 28 exhibited poorer cognitive outcome, in particular executive functions. Likewise, participants who were born moderately preterm but small for gestational age, underperformed both with regards to executive functions and general cognitive abilities.

Impaired executive functions in adolescents and young adults born preterm are also reported by others [9–11], and these studies suggest that the influence of preterm birth on early brain development has life-long consequences. A meta-analysis [12] of a large number of functional magnetic resonance imaging (fMRI) studies suggests that executive functions are controlled by a bilateral network in the brain, involving dorsolateral prefrontal, anterior cingulate, and parietal cortices. The widespread fronto-cingulate-parietal network require well-functioning pathways connecting the involved cortical regions. The importance of these pathways is supported by structural MRI studies. For example, Seghete et al [13] examined typically developing adolescents with Diffusion Tensor Imaging (DTI) and found associations between two aspects of executive function (inhibition and task-switching) and fractional anisotropy (FA; often used as a surrogate measure of white matter organisation) in regions that support cross-cortical and cortical-subcortical connections from the prefrontal cortex. Diffusion MRI studies in preterm born children and adolescents, including our own [14], have shown that white matter is affected in several brain regions, including parts of the corpus callosum and external and internal capsules, as well as the superior fronto-occipital fasciculus, superior longitudinal fasciculus, posterior thalamic radiation and left cingulum and posterior corona radiata (see [15] for review). Several of these areas contain fibre tracts that connect the various cortical regions involved in the “executive network”. Disturbances of these pathways may thus influence the efficiency of the network resulting in poorer executive functioning observed in preterm born children and adolescents.

In the present study, we wanted to investigate relationships between brain white matter microstructure and cognitive functions, particularly executive functions, in the cohort of preterm adolescents for which we have recently reported cognitive outcome at adolescent age [8]. The specific hypothesis was based on this previous studies in this cohort, which showed that preterm born participants had perturbed executive functions, and that this was a robust finding at both the 5.5 year assessments [16] and the 18 year assessments [8]. Furthermore, in an earlier MRI study of a subsample of the SNP cohort we found altered total and regional grey and white matter volumes, alterations in white matter microstructure [14], and alterations in cortical thickness [17] in comparison to the term born controls. Findings from existing studies on preterm adolescents and/or young adults, using DTI to examine white matter microstructure, suggest an association between atypical white matter organisation and executive function deficits [18]. However, the existing studies often include participants with brain injury according to conventional structural MRI, and those born small for gestational age, which might introduce some bias in the sample. Therefore, in the present study only preterm born adolescents with appropriate for gestational age birth weight and without signs of preterm brain injury on radiological assessment of MRI were included. Our main hypothesis was that there is a correlation between measures of executive functioning and FA in pathways connecting the cortical regions that are involved in executive functions. In addition, we investigated relationships between FA and measures of general intelligence, which was also affected in this cohort. Finally, we explored whether variations in regional white and grey matter volumes in preterm born individuals were related to general intelligence and/or executive functions.

## Methods

### Participants

The preterm participants of this study are part of the cohort that forms the Stockholm Neonatal Project (SNP), a longitudinal, population-based study of children born preterm with very low birth weight (< 1501g, VLBW) between 1988–93, at Karolinska Hospital or Löwenströmska Hospital in Stockholm, Sweden. The gestational age range of the SNP cohort is 23–36 weeks (mean 28.1). This cohort has been followed prospectively since birth. The SNP cohort was psychologically assessed at 10 months [19] and 5 ½ years of age [16, 20]. At the age of 18 years (mean age at testing 18 years ± 2 weeks), a total of 134 preterm born and 94 term born controls participated in a comprehensive psychological assessment [8, 21]. The control children were born on the same day and at the same hospital as the preterm children, had a gestational age of at least 37 weeks, a birth weight of at least 2500 g, and were classified as a healthy baby at birth. Of these, 71 preterm adolescents and 63 term born controls underwent MRI (mean age at scanning 15.2 years, SD 1.6, range 12–18 years). Eleven preterm participants who were born small for gestational age (SGA, defined as more than 2 SD below the population mean) were excluded from the analysis to avoid confounding effects caused by intrauterine growth retardation [8]. At a later stage (see below for details), eight participants were excluded due to radiological signs of preterm brain injury. There were no significant differences between the preterm born (mean gestational age 27.6, SD 2.3 weeks; birth weight 1049, SD 256 grams; 47.1% male) and the term born group (mean gestational age 40, SD 1.2 weeks; birth weight 3543, SD 83 grams; 52.9% male) in distribution of sex, age at scanning, demographic and socioeconomic background, the residential area, schooling, parental education, or mother’s age at birth. The preterm subsample that did not participate in MRI scanning differed significantly in birth weight and in some cognitive measures from the subsample that underwent neuroimaging and for whom good quality MRI data were available (see S1 Table), with significant differences in all three measures of attention and speed (coding, symbol search, trail making test), and in one measure of cognitive flexibility (colour wording test).

### Ethics approval

The longitudinal study was originally approved by the Ethics committee at Karolinska Hospital, and cognitive assessments at age 18 years, MRI, as well as continued use of the original data base was approved by the Regional Ethics Board of Stockholm (2007/46-31/3 and 2009/1229-32). All participants gave their written consent prior to the data collection.

### Measures

#### Cognitive measures

The assessment involved an extensive battery of cognitive tests, described in detail elsewhere [8]. In the present study we used data from the Wechsler Intelligence Scale for Children III (WISC-III, [22]), which provides verbal (VIQ), and performance (PIQ) measures. Three aspects of executive function were assessed, i.e. working memory (digit span and arithmetic from WISC-III; Corsi block from the Wechsler Adult Intelligence Scales III NI, [23]); attention and speed (coding and symbol search from the WISC-III, trail making 1 from the Delis-Kaplan Executive Function Systems, D-KEFS, [24]); cognitive flexibility (trail making 3, verbal fluency, design fluency and colour word 3 from D-KEFS). Group comparisons for the cognitive measures were performed using Student’s t-test.

### Neuroimaging

#### MRI data acquisition

Data were acquired on a 1.5 T MR-system (Signa Excite Twinspeed, GE,Waukesha, WI, USA) at Karolinska University Hospital in Stockholm, Sweden. The majority of the data were acquired between April 2005 and February 2006 (n = 119). The remaining data were acquired between October 2009 and December 2009 (n = 15). In between these two data collection periods, the scanner underwent a software upgrade. An analysis including the scanner as a confounding variable was performed and showed that this did not have an effect. The protocol included the following sequences: a high resolution 3D T1-weighted (w) Spoiled Gradient Echo (3D SPGR) image with TR = 24ms, TE = 6 ms, flip angle 30°, voxel size 0.98x0.98x1.5 mm^3^; axial Turbo Spin Echo T2-w images (TE = 85 ms, TR = 6000 ms, flip angle 90°, voxel size 0.98x0.98x4.0 mm^3^), and coronal Fluid Attenuated Inversion Recovery (FLAIR) images (TE = 140 ms, TR = 9000 ms, slice thickness 4 mm), and a 2D twice-refocused Spin Echo Diffusion dataset (30 directions, b = 1000 s/mm^2^, 4 reference images, b = 0 s/mm^2^, TE = 72 ms, flip angle = 90°, voxel size 1.96 x 1.96 x 3.0 mm^3^).

Images were visually inspected for quality (movement artifacts, signal dropouts). Eleven 3D T1-w datasets (all from preterm participants) and 15 DTI datasets (11 preterm and 4 term born controls) of the original 134 datasets were excluded because of poor quality. The T1-w and T2-w images were assessed for radiological signs of preterm brain injury (i.e. ventricular dilatation, periventricular white matter abnormalities consisting of high signal on T2-w images), and eight datasets from the original number of good quality datasets from the preterm group were excluded (n = 2 ventricular dilatation, n = 6 periventricular white matter abnormalities). None of the term born control datasets had radiological abnormalities. After exclusion of datasets from participants who were SGA at birth, those who had radiological signs of preterm brain injury on visual inspection of MRI, and the data sets with poor quality, the final dataset consisted of 52 preterm participants and 63 full term controls for voxel-based morphometry analyses, and 52 preterm participants and 59 term controls for the DTI analyses.

#### MR data processing and analysis

The 3D T1-w datasets were pre-processed and analysed in SPM8 using the VBM8 toolbox [25]. After checking the raw images for quality, images were first re-aligned, then normalised (using DARTEL [26]). A modulation step was included to adjust for the brain volume changes resulting from the normalisation. Images where then segmented into grey matter (GM), white matter (WM), and cerebro-spinal fluid. The GM and WM segments were smoothed with a 6 mm FWHM Gaussian kernel prior to voxel-wise statistical analyses. First, group comparisons between the preterm and the term born control group were conducted with age at scanning and sex as covariates. Second, correlation analyses, separately within the preterm and within the term born group, investigating correlations between the measures VIQ, PIQ, as well as the executive function domains working memory, attention and speed, and cognitive flexibility, with GM and WM volumes, with age at scanning and sex as covariates were performed. Regions were localised with the help of WFU Pickatlas [27]. Only clusters with a voxel number of > 10 were considered, and results were corrected for multiple comparisons using family-wise error (FWE) correction. Probability values < 0.05 were considered statistically significant.

The Diffusion MR data sets were corrected individually for Eddy current artifacts and realigned. The mean of the four reference images was used to estimate the extent of susceptibility-induced distortion. The two data sets were then combined into a single undistorted series, from which the diffusion tensor and fractional anisotropy (FA) maps were calculated.

The processing and statistical analysis of the FA images followed the Tract-Based Spatial Statistics pipeline (TBSS [28]) made available through the FMRIB Software Library (FSL, [29]) software package. Utilising a non-linear registration, the FA images of all participants were aligned and resampled to a standard-space image with 1-mm isotropic voxels. A minimum threshold FA value of 0.2 was used. Because the null distribution was not known and the images had not been smoothed, permutation methods were used on the cluster level using the randomise tool in FSL. Student’s t-tests were used to compare the preterm and the control group. Correlation analyses, controlling for age at scanning and sex, between the cognitive measures VIQ and PIQ as well as the three executive function indices and the voxel-wise FA values were performed. Sets of separate analyses were performed for the preterm group and the term born control group. Probability values of p < 0.05 were considered statistically significant for the raw probability images, and threshold free cluster enhancement, TFCE, (fully corrected for multiple comparisons across space) was applied.. Regions were localised with the help of the JHU white matter tractography atlas and JHU ICPM_DTI-81 white matter labels [30–32].

## Results

### General intelligence and executive functions

Table 1 shows the participants’ measures of general intelligence and executive functions. In line with the findings for the whole cohort [8], the preterm group scored significantly lower than the controls on the IQ scales, all three attention and speed tests, all three working memory tests, and for two of the cognitive flexibility tests (verbal fluency and trail making test 3).

**Table 1:** Participants’ measures of general intelligence and executive functions.

	Preterm-born	Term-born	Comparison preterm-born versus term-born group *
	n = 52	n = 63	
	Mean (SD)	Mean (SD)	p-value
***General Intelligence***			
*Full Scale IQ (WISC-III)*	90.8 (21.2)	96.9 (17.0)	0.037
*Performance IQ (WISC-III)*	90.0 (23.7)	95.5 (20.0)	0.036
*Verbal IQ (WISC-III)*	94.2 (7.1)	99.2 (15.0)	0.039
***Executive functions***			
Attention and speed			
*Coding (WISC-III)*	63.1 (12.8)	66.2 (12.8)	0.042
*Symbol search (WISC-III)*	33.2 (7.2)	35.5 (5.5)	0.047
*Trail making test 1 (D-KEFS)*	8.8 (3.4)	9.7 (3.0)	0.038
Working memory			
*Digit span (WISC-III)*	14.2 (3.2)	15.4 (3.1)	0.018
*Arithmetic (WISC-III)*	20.0 (3.2)	21.4 (3.5)	0.027
*Corsi block (WAIS-III NI)*	16.9 (3.1)	18.1 (2.8)	0.020
Cognitive flexibility			
*Verbal fluency (D-KEFS)*	10.8 (3.5)	11.7 (3.5)	0.024
*Design fluency (D-KEFS)*	31.3 (6.3)	31.7 (7.0)	0.908
*Color word test (D-KEFS)*	8.7 (3.0)	9.5 (3.1)	0.310
*Trail making test 3 (D-KEFS)*	1 (4.0)	8.9 (2.9)	0.008

*Student’s t-test

WISC-III: Wechsler Intelligence Scale for Children, 3^rd^ edition; raw scores

WAIS-III NI: Wechsler Adult Intelligence Scale, III NI; raw scores

D-KEFS: Delis-Kaplan Executive Function System; scaled scores

#### Regional GM and WM volumes

Voxel-wise comparisons of GM showed smaller volumes in the preterm group compared to the term group in the frontal lobe (left middle frontal and right Rolandic area); the parietal and occipital lobe (left post-central region and occipital superior gyrus), and the temporal lobe (bilateral middle temporal gyrus, temporal inferior gyrus). The preterm group had larger GM volumes in the temporal lobe (right and left fusiform gyrus), the limbic lobe, the anterior cingulate, the right insula, and right anterior amygdala. White matter volumes were smaller in the preterm group within the temporal lobe (right fusiform gyrus, right and left middle temporal gyrus, left inferior temporal gyrus), but larger in the right insula.

### Correlations between regional brain volumes and executive function measures

In the preterm group there were no positive or negative correlations between any of the executive function measures and GM or WM volumes at corrected (FWE, p < 0.05) level. In the term born group, there were no significant positive or negative correlations between any of these measures and GM at corrected level. For WM, there was a significant positive correlation (FWE corrected, p = 0.015) with the score for attention and speed in the left frontal lobe (middle frontal gyrus; cluster of only 10 voxels).

### Correlations between regional brain volumes and general intelligence measures

In neither the preterm group nor the term group were there any positive or negative correlations between VIQ or PIQ measures and GM or WM seen after correction for multiple comparisons (FWE, p < 0.05).

### Fractional anisotropy (FA) in WM

The preterm group had lower FA than the term born group in the inferior longitudinal fasciculus bilaterally, the external capsule bilaterally (inferior fronto-occipital fasciculus, uncinate fasciculus), the right anterior limb of the internal capsule, the left fornix, and the left occipital WM. There was no significant difference for the opposite contrast in any of the analyses.

### Correlations between FA and executive function measures

**Fig 1:**
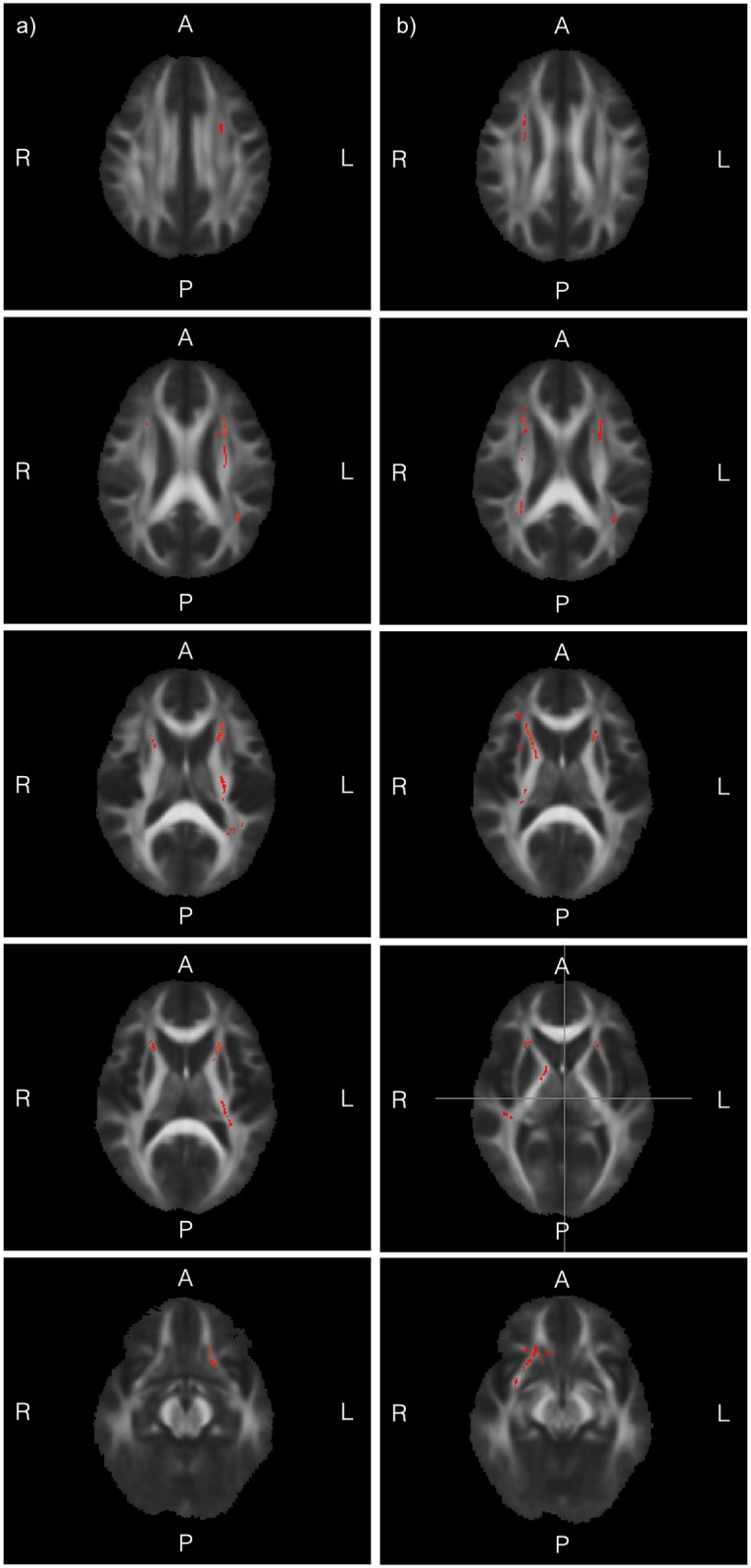
(a and b): White matter tracts for which there was positive correlation between FA and executive function measures in the preterm group. Fig 1a: Results of TBSS analyses examining correlations between FA and working memory scores, superimposed on a group mean FA image in axial orientation. Yellow-red areas indicate white matter tract regions in which positive correlations were seen at p < 0.05 (TFCE; corrected for multiple comparisons); results are controlled for age at MRI and sex. For details of regions, see Table 2. Fig 1b: Results of TBSS analyses examining correlations between FA and cognitive flexibility score, superimposed on a group mean FA image in axial orientation. Yellow-red areas indicate white matter tract regions in which positive correlations were seen at p < 0.05 (TFCE; corrected for multiple comparisons); results are controlled for age at MRI and sex. For details of regions, see Table 2.

Results from the correlation analyses are shown in Table 2 and in Fig 1a and 1b.

**Table 2:** Correlations between FA and measures of general intelligence and executive functions.

JHU white matter tractography atlas/ICBM-DTI-81 white matter-labels	Side	^1^Group difference in FA^#^	^2^VIQ	^2^PIQ	^2^Working Memory score	^2^Cognitive Flexibility score
Uncinate fasciculus	right	x	-	-	x	x
Uncinate fasciculus	left	x	x	x	x	x
Inferior fronto-occipital fasciculus	right	x	x	-	x	x
Inferior fronto-occipital fasciculus	left	x	x	x	x	x
Inferior longitudinal fasciculus	right	x	-	-	x	-
Inferior longitudinal fasciculus	left	x	-	-	x	-
Superior longitudinal fasciculus	right	-	x	-	-	x
Superior longitudinal fasciculus	left	-	x	x	x (temporal part)	x
Retrolenticular part of posterior limb of internal capsule	left	-	x	-	x	x
Anterior limb of internal capsule	right	x	-	-	-	-
Corticospinal tract	right	-	-	-	-	x
Corticospinal tract	left	-	x	x	x	x
Anterior thalamic radiation	right	-	x	-	x	x
Anterior thalamic radiation	left	-	x	x	x	x
External capsule	right	x	-	-	-	x
External capsule	bilateral	x	-	-	-	-
Forceps minor	bilateral	x	-	-	x	-
Fornix	left	x	-	-	-	-
Occipital white matter	left	x	-	-	-	-

^1^ Group comparison (preterm born versus full term born participants) of fractional anisotropy (FA) on a whole brain level using Tract-based Spatial Statistics (TBSS). X = areas in which significant group differences were seen at p < 0.05 (TFCE, corrected for multiple comparisons)); results are controlled for age at MRI and sex.

^#^ = FA lower in preterm group (no significant findings of higher FA in preterm group).

^2^ Correlation analyses examining correlations between FA and cognitive scores (VIQ = Verbal IQ, PIQ = Performance IQ; working memory, cognitive flexibility scores) in the preterm group. X = areas in which positive correlations were seen at p < 0.05 ((TFCE; corrected for multiple comparisons); results are controlled for age at MRI and sex. There were no significant correlations for FA and attention and speed scores after correction for multiple comparisons, therefore this is not included in this table.

In the preterm group significant positive correlations between working memory scores and FA were present in the uncinate fasciculus bilaterally; the forceps minor, the retrolenticular part of the posterior limb of the internal capsule on the left; on the level of the anterior limb of the internal capsule bilaterally in the anterior thalamic radiation and the inferior fronto-occipital fasciculus, and the inferior longitudinal fasciculus bilaterally; on the level of the superior corona radiata the cortico-spinal tract and the superior-longitudinal fasciculus (temporal part) on the left; the anterior thalamic radiation bilaterally.

For the cognitive flexibility scores, significant positive correlations with FA were seen in the uncinate fasciculus bilaterally, the inferior fronto-occipital fasciculus bilaterally, the superior longitudinal fasciculus bilaterally; on the level of the internal capsule the right cortico-spinal tract, and the retrolenticular part on the left; the anterior thalamic radiation bilaterally; the right external capsule; on the level of the superior corona radiata the cortico-spinal tract, and the anterior thalamic radiation bilaterally.

There were no significant positive correlations between FA and attention and speed scores at corrected level, although there were some trends in the right anterior thalamic radiation, the right inferior fronto-occipital fasciculus and the right superior longitudinal fasciculus (p < 0.06). There were no significant negative correlations between FA and any of the executive function scores at corrected level.

In the term born group, no significant positive or negative correlations between any of the executive functions measures and FA were seen after correction for multiple comparisons.

### Correlations between FA and general intelligence measures

**Fig 2:**
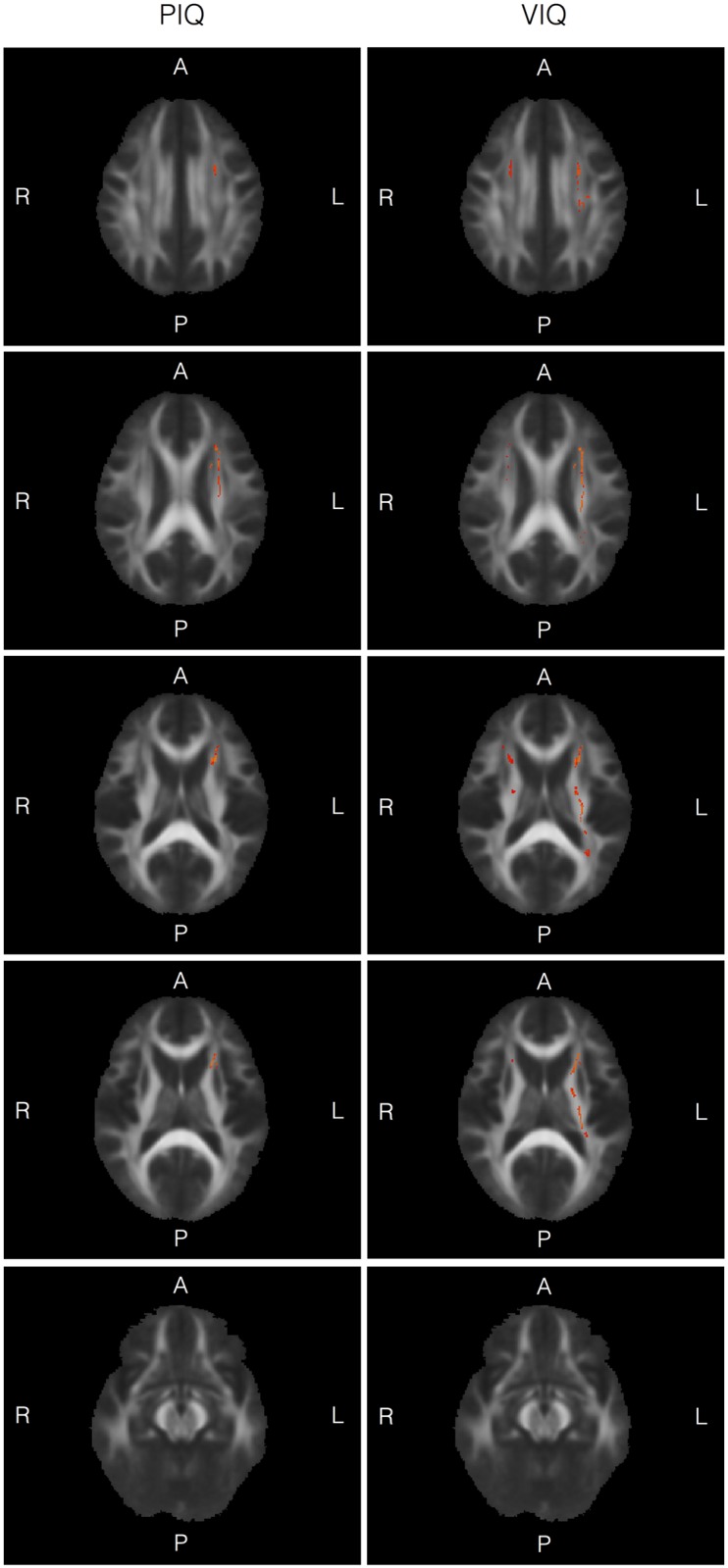
(a and b): White matter tracts for which there was positive correlation between FA and general intelligence measures in the preterm group. Fig 2a: Results of TBSS analyses examining correlations between FA and Performance IQ, superimposed on a group mean FA image in axial orientation. Yellow-red areas indicate white matter tract regions in which positive correlations were seen at p < 0.05 (TFCE; corrected for multiple comparisons). Results are controlled for age at MRI and sex. For details of regions, see Table 2. Fig 2b: Results of TBSS analyses examining correlations between FA and Verbal IQ, superimposed on a group mean FA image in axial orientation. Yellow-red areas indicate white matter tract regions in which positive correlations were seen at p < 0.05 (TFCE; corrected for multiple comparisons). Results are controlled for age at MRI and sex. For details of regions, see Table 2.

Fig 2a and 2b and Table 2 show areas with significant correlations between measures of intelligence (VIQ, PIQ) and FA. In the preterm group, there were significant positive correlations between FA and both IQ measures in the left uncinate fasciculus, left cortico-spinal tract, left inferior-frontal fasciculus, left superior-longitudinal fasciculus, and left anterior thalamic radiation. For VIQ, there were additional positive correlations with FA measures in the inferior fronto-occipital fasciculus bilaterally, superior longitudinal fasciculus bilaterally (left more than right); and the retrolenticular part of the posterior limb; the anterior thalamic radiation bilaterally (left more than right).. Finally, for PIQ, there were additional positive correlations with FA measures in the left inferior fronto-occipital fasciculus. No negative correlations between FA and IQ scores were seen at corrected level.

In the term born group, there were no positive or negative correlations between FA and IQ measures after correction for multiple comparisons.

## Discussion

The main finding of this study is that there were strong correlations between microstructure of multiple WM tracts and several cognitive functions in the preterm adolescents, whereas this was not seen in the term born controls after correction for multiple comparisons. Importantly, these correlations were seen in a sample from which those preterm participants who were born SGA and/or had radiological signs of preterm brain injury were excluded. The well-defined and characterised cohort with stable cognitive abilities between the 5.5 year and 18 year assessments, the rigorous inclusion and exclusion criteria, and the application of rigorous statistical correction for multiple comparisons in the neuroimaging analyses, are particular strengths of this study. Overall, our findings provide additional strong evidence that the observed differences in cognitive functioning between persons born preterm and those born at term are a consequence of widespread altered brain connectivity, still present many years after preterm birth. Furthermore, our findings also support the notion that preterm birth per se induces altered brain development, with long lasting anatomical and behavioural consequences.

Interestingly, the areas for which correlations between cognitive measures and WM structure were seen were also areas in which group differences in WM structure between the preterm and term born participants were detected. It is tempting to infer a causal relationship, i.e., that disturbance of the neural pathways in these specific areas are the primary cause of the impaired cognitive functions. However, our data can only show associations, and there might be other primary causes, e.g. neuronal death in cortical areas resulting in secondary changes of the axons. Against that, however, speaks that we could only find minor changes in GM, and that those did not show correlations with cognitive measures. While several GM areas differed in volume between the preterm and the term born group, there were no significant correlations between GM volumes and cognitive measures once corrected for multiple comparisons. White matter volumes were also different between the two groups, but in contrast to the GM volumes, there was a significant correlation between left frontal lobe (middle frontal gyrus) volume and the score for attention and speed in the term born group.

When examining correlations of FA measures with cognitive measures, in the preterm group significant positive correlations of both IQ measures and, in particular, two of the three executive function measures (namely working memory scores and cognitive flexibility scores) were seen, while no such correlations were seen in the term born group. Intact WM microstructure appears particularly crucial in relation to functional outcome after preterm birth, while normal variation in term born children does not seem to interfere. Fractional anisotropy is a measure that is often used to infer on WM microstructure and is influenced by a variety of factors [33], which include, amongst others, distribution of axonal diameters, axonal density, and myelination status. In our study the alterations in WM microstructure involved long association fibres linking frontal and temporal lobe, frontal and parietal lobe, and the temporal and occipital lobe, as well as projection fibres (cortico-spinal tract and anterior thalamic radiation). Changes were often bilateral, but overall more pronounced in the left hemisphere. For the attention and speed measures, only trends were seen for correlations with FA in long association (right inferior fronto-occipital fasciculus, and superior longitudinal fasciculus) fibres and projection fibres (right anterior thalamic radiation). The overlapping areas for the three executive function measures is not surprising, since the tasks reflect a common executive factor as well as specific executive abilities. Overlapping areas were also described by e.g., Tamnes et al, [34], in a study examining cortical thickness and associations with executive function measures in children and adolescents.

Neuroimaging and lesion studies have shown that executive functions are not solely located in the frontal lobe. It has been proposed that the prefrontal cortex serves as a modulator, subserved by posterior cortical regions, dependent on distributed neural networks including fronto-parietal and fronto-striatal pathways (see e.g. [35, 13], for review). Stevens and Skudlarksi [36] have examined adolescents and adults and could show with a multimodal imaging approach (including measurement of FA) that the maturation of executive functions is directly associated with the relationship between WM development and age-related changes in the functional interaction of neuronal networks. They suggested that executive functions develop as a more widely distributed network is engaged (fronto-cingulate-parietal, fronto-striatal networks), depending on the greater coherence of WM that occurs with maturation. Such neural networks rely on optimally functioning long distance connections (see e.g. [37] for review), i.e. intact WM pathways. Factors that influence growth and development of WM tracts that provide the connections between the distributed neural networks, will thus have an effect on network function and, consequently, on cognition and behaviour.

There is strong evidence from histopathology that preterm birth carries a considerable risk not only for focal periventricular white matter injury, but also for long term alterations in WM with accompanying axonal and neuronal changes in the cortex, thalamus, basal ganglia, brainstem, and the cerebellum (see [38] for review). Neuroimaging studies using diffusion MRI (mainly DTI) and volumetric analyses, from neonatal age (e.g [39,40]) to adolescence/young adulthood (see e.g. [41, 14, 42, 43]) further support that preterm birth, even in those who survive without major focal brain injury, is associated with primary or secondary altered WM development as well as with reduced cortical volumes in several brain regions (see e.g. [44,45] for review). Taken all this together, it appears very likely that preterm birth is associated with structural, and subsequent functional, impairment of large scale networks involved in cognitive, behavioural, and motor processes.

The findings in our study are, overall, in accordance with the few existing studies on brain structure—executive function associations in preterm adolescents or young adults, although studies at that age are still limited in number and, in addition, are only comparable to a certain extent since there is a considerable variation in tests employed, executive function models used, as well as imaging acquisition, processing, and analysis techniques. This might also partly explain why some studies have not found any associations between measures of altered WM structure and performance on executive function tests [46, 47], although in the study by Allin et al [46] global memory scores were associated with FA measures in several association and projection pathways.

Although we detected significant group differences in regional brain volumes, we did not find significant correlations between regional WM or GM volumes and any of the cognitive measures after controlling for multiple comparisons. Other studies, for example, Nosarti et al, [42], have found associations between both regional GM and WM volume alterations and performance on tests of executive function (mainly cognitive flexibility) in early adulthood in participants born very preterm. It is also of interest that in this subsample of the SNP cohort we did not find differences in FA measures or volume measures of the corpus callosum, which has been reported in some other studies of preterm adolescents/young adults, including in a slightly different subsample of the SNP cohort [14], which, however, included participants with radiological signs of preterm brain injury, and once those were excluded from the analyses such differences were no longer seen. In the study by Nosarti et al [42], for example, the smaller volume of the posterior corpus callosum in the preterm group explained a large proportion of the variation in executive function measures, something that has also been described by Narberhaus et al [48], although in that study correlations were mainly found with anterior parts of the corpus callosum. A possible explanation could be that, in contrast to most studies, we have excluded participants who were born small for gestational age or had any radiological signs of preterm brain injury, and that we applied rigorous correction for multiple comparisons in our VBM analyses. In addition, although clear differences in cognitive function between the preterm born and the term born participants are present in the SNP cohort, these are relatively subtle and this might partly explain why detected correlations in the VBM analyses were only seen at uncorrected level and did not survive correction for multiple comparisons.

An important factor that might have influenced the results of our study is that the age range at time of neuroimaging was 12–18 years. It is well known that both GM and WM continue to develop, with GM volume peaking during childhood, and WM volume increasing into young adulthood, with sex-specific trajectories (e.g. [49]). Similarly, FA continues to increase to adolescence and adulthood [50] for review). However, we have taken this into account in the analyses by controlling for age at scanning and for sex.

In conclusion, this study provides strong evidence that in persons born preterm, in the absence of radiological signs of preterm brain injury, widespread alterations in regional brain tissue volumes and white matter microstructure are still present in adolescence/young adulthood. Importantly, the observed microstructural alterations in projection fibres and the long association fibres linking frontal, temporal, and parietal lobes and also temporal and occipital lobes, are correlated with measures of general cognitive abilities and executive function measures. Based on this, our findings suggest that early growth disturbance of the neural pathways, rather than changes in regional brain volumes, are the primary cause of poorer cognitive functions in preterm born persons.

## Supporting information

S1 Table S1 Table shows comparison of cognitive measures, perinatal variables and parental education between those who did and those who did not participate in neuroimaging. (DOCX)

S1 File S1 File contains copies of papers that have used SNP cohort data. (ZIP)
