# Supplementary material for: Correlation between white matter microstructure and executive functions suggests early developmental influence on long fibre tracts in preterm born adolescents
Source: PLoS One. 2017 Jun 8;12(6):e0178893. doi: 10.1371/journal.pone.0178893 (PMC5464584; doi:10.1371/journal.pone.0178893)
Supplement: S1 Table — (DOCX) [file pone.0178893.s001.docx]

| Table S1: Comparison between those who did and those who did not participate in neuroimaging with regards to cognitive measures, perinatal variables and parental education. | | | |
| --- | --- | --- | --- |
|  | Preterms with MRI**  n = 52 | Preterms without MR**  n = 57 | p-value* |
| Gestational age at birth in weeks; mean (SD) | 27.5 (2.2) | 27.2 (2.4) | 0.32 |
| Birth weight; mean (SD) | 1053 g (225) | 915 g (209) | .004 |
| Sex (boys), n (%) | 25 (48.1) | 24 (42.1) | 0.62 |
| ROP stage 3+, n (%) | 6 (11.5) | 6 (10.5) | - |
| CLD , n (%) | 5 (9.6) | 7 (12.3) | - |
| Mother’s education^$^; mean (SD) | 4.2 ( 1.3) | 4.1 (1.5) | 0.65 |
| Father’s education^$^; mean (SD) | 4.1 (1.5) | 4.0 (1.4) | 0.86 |
| ***General cognitive function*** |  |  |  |
| WISC-III: Full Scale IQ; mean (SD) | 90.3 (21.2) | 87.6 (20.5) | 0.40 |
| WISC-III: Performance IQ; mean (SD) | 90.0 (23.7) | 82.2 (22.1) | 0.07 |
| WISC-III: Verbal IQ; mean (SD) | 94.2 (17.1) | 94.7 (13.2) | 0.90 |
| ***Executive functions*** |  |  |  |
| Attention & Speed  *- Coding (WISC-III);* mean (SD)  *- Symbol Search (WISC-III);* mean (SD)  *- Trail making test* 1 *(D-KEFS)*; mean (SD) | 63.1 (12.8)  33.2 (7.2)  8.8 (3.4) | 58.1 (13.0)  30.0 (7.5)  7.5 (3.3) | 0.04  0.01  0.03 |
| Working memory  *- Digit span (WISC-III)*; mean (SD)  *- Arithmetic (WISC-III)*; mean (SD)  *- Corsi block (WAIS)*; mean (SD) | 14.2 (3.2)  20.0 (3.2)  16.9 (3.1) | 14.1 (3.2)  19.8 (3.8)  15.9 (3.2) | 0.79  0.75  0.07 |
| Cognitive Flexibility  *- Verbal fluency (D-KEFS)*; mean (SD)  *- Design fluency (D-KEFS)*; mean (SD)  *- Color word test (D-KEFS)*; mean (SD)  *- Trail making test 3(D-KEFS)*; mean (SD) | 10.8 (3.5)  31.3 (6.3)  8.7 (3.0)  7.1 (4.0) | 9.7 (3.2)  29.0 (6.3)  7.5 (3.7)  6.3 (3.7) | 0.10  0.06  0.02  0.12 |

*Student’s t-test; ** Participants born SGA are excluded from this study

^$^ Education according to Statistics Sweden. 0=no formal education; 1=did not complete elementary school; 2=graduated from junior high school; 3=completed two years of high school; 4= completed three years of high school; 5=Bachelor’s degree; 6=Master’s degree; 7=doctoral degree

WISC-III: Wechsler Intelligence Scale for Children, 3^rd^ edition; raw scores

WAIS-III NI: Wechsler Adult Intelligence Scale, revised; raw scores

D-KEFS: Delis-Kaplan Executive Function System; scaled scores

ROP= Retinopathy of prematurity; CLD= chronic lung disease
