# Supplementary material for: Correlation between white matter microstructure and executive functions suggests early developmental influence on long fibre tracts in preterm born adolescents
Source: PLoS One. 2017 Jun 8;12(6):e0178893. doi: 10.1371/journal.pone.0178893 (PMC5464584; doi:10.1371/journal.pone.0178893)
Supplement: S1 File — (ZIP) [file pone.0178893.s002.zip › New folder/Chu et al_2015.pdf]

RESEARCH ARTICLE

Investigating the Use of Support Vector Machine Classification on Structural Brain Images of Preterm–Born Teenagers as a Biological Marker

Carlton Chu[1,4], Hugo Lagercrantz[2], Hans Forssberg[2], Zoltan Nagy[3,4]*

1 DeepMind Technologies Ltd., London, United Kingdom, **2** Department of Women's and Children's Health, Karolinska Institutet, Stockholm, Sweden, **3** Laboratory for Social and Neural Systems Research, University of Zurich, Zurich, Switzerland, **4** Wellcome Trust Centre for Neuroimaging, UCL Institute of Neurology, London, United Kingdom

* zoltan.nagy@uz.ch

CrossMark
click for updates

OPEN ACCESS

Citation: Chu C, Lagercrantz H, Forssberg H, Nagy Z (2015) Investigating the Use of Support Vector Machine Classification on Structural Brain Images of Preterm–Born Teenagers as a Biological Marker. PLoS ONE 10(4): e0123108. doi:10.1371/journal.pone.0123108

Academic Editor: Satoru Hayasaka, Wake Forest School of Medicine, UNITED STATES

Received: October 31, 2014

Accepted: February 27, 2015

Published: April 2, 2015

Copyright: © 2015 Chu et al. This is an open access article distributed under the terms of the Creative Commons Attribution License, which permits unrestricted use, distribution, and reproduction in any medium, provided the original author and source are credited.

Data Availability Statement: Data contain patient-identifying information and are prevented from being shared by ethical restrictions. Requests for anonymised data may be sent to Professor Hans Forssberg (hans.forssberg@ki.se).

Funding: Funding support was provided by Sällskapet Barnavård (Sweden), the Swedish Research Council, the Bank of Sweden Tercentenary Foundation and the Wellcome Trust (079866/Z/06/Z). The funders had no role in study design, data collection and analysis, decision to publish, or preparation of the manuscript. DeepMind

Abstract

Preterm birth has been shown to induce an altered developmental trajectory of brain structure and function. With the aid support vector machine (SVM) classification methods we aimed to investigate whether MRI data, collected in adolescence, could be used to predict whether an individual had been born preterm or at term. To this end we collected T1–weighted anatomical MRI data from 143 individuals (69 controls, mean age 14.6y). The inclusion criteria for those born preterm were birth weight $\leq$ 1500g and gestational age $<$ 37w. A linear SVM was trained on the grey matter segment of MR images in two different ways. First, all the individuals were used for training and classification was performed by the leave–one–out method, yielding 93% correct classification (sensitivity = 0.905, specificity = 0.942). Separately, a random half of the available data were used for training twice and each time the other, unseen, half of the data was classified, resulting 86% and 91% accurate classifications. Both gestational age (R = –0.24, p<0.04) and birth weight (R = –0.51, p $<$ 0.001) correlated with the distance to decision boundary within the group of individuals born preterm. Statistically significant correlations were also found between IQ (R = –0.30, p $<$ 0.001) and the distance to decision boundary. Those born small for gestational age did not form a separate subgroup in these analyses. The high rate of correct classification by the SVM motivates further investigation. The long–term goal is to automatically and non–invasively predict the outcome of preterm–born individuals on an individual basis using as early a scan as possible.

Introduction

Following the long–term outcome of individuals that were born preterm, and in particular their structural and functional brain development, has been an active area of research in pediatrics. Some studies are descriptive in nature, identifying the issue at hand [1–8], while others

Technologies Ltd., provided support in the form of salary for author Carlton Chu, but did not have any additional role in the study design, data collection and analysis, decision to publish, or preparation of the manuscript. The specific role of this author is articulated in the 'author contributions' section.

Competing Interests: The authors have the following interests: the major part of the work was conceived and performed while Dr. Chu worked at the Functional Imaging Laboratory within the University College London. Dr. Chu now works at and receives his salary from DeepMind Technologies Ltd and his efforts in finishing this paper have been expanded in his free time. There are no patents, products in development or marketed products to declare. This does not alter the authors' adherence to all the PLOS ONE policies on sharing data and materials.

actively investigate the efficacy of certain markers in predicting short—or long–term developmental outcome [3,9–11].

While the descriptive studies show a statistically significant difference between the groups of control and preterm–born individuals, there is often a wide distribution in the variable of interest in both groups [3,5,6], where some members of each group could easily belong to the other group based on the measure investigated. Clearly a more individualized method would be desirable. In investigations into specific markers of outcome the results can be much more specific to individuals who took part in the respective studies, however, such investigations usually involve considerable manual input, which cannot be followed on a day–to–day basis.

There has been significant recent interest and development in machine–learning methods, which address both of the above aspects. I.e. the results can be applied on an individual basis and with minimal user intervention. One well–known and widely used variant is support vector machines (SVMs) [12–15]. The employment of SVMs in pediatric research is not unprecedented [16,17], albeit MRI–based pattern recognition is still new in this field. For example, Franke et al. [18] used a related approach to establish normal aging processes in healthy children and adolescents and included a group of preterm born adolescents to test their method. In other fields, e.g. degenerative neurological conditions (see for example [19–21]) and in developmental cognitive neuroscience [22] SVM methods have been used on MR images. Briefly, the first step in using SVMs for automatic classification of images is to use a dataset where the group adherence of each subject with an MRI dataset is known. The result of this training is to identify an optimal boundary between the case and control groups in a multidimensional space (Fig 1). Future, datasets can be classified, based on the so defined boundary during the training step.

Of course the goal is not simply to identify individuals as preterm–born or term–born. That is already known at birth and needs no further investigation. Rather, the long–term goal for such methods is to predict from MR images whether a preterm–born individual has brain structure that is more indicative of a preterm–born or term–born person. Successful identification of brains by the SVM is a necessary condition for future success of the method toward that goal. Therefore, the aim of this study was to use a structural MRI images from a cohort of individuals who had been born preterm and matched controls to investigate how successfully the SVM methodology could be used to correctly identify to which group an individual belonged.

Methods

This study was performed with the approval of the regional ethics board of Stockholm North. One of the parents or a legal guardian signed a written, informed consent form for each participant before the MRI examination.

The MRI data used here were collected as part of a previously published study [6], in which preterm–born individuals and matched controls underwent an MRI examination in adolescence. The case group consisted of individuals who were enrolled into the Stockholm Neonatal Project [23] at birth. The inclusion criteria were birth weight (BW) $\leq$ 1500 g and gestational age (GA) $\leq$ 36 weeks. At the age 5.5 years, during a psychological follow–up, a control group was recruited from a population based register given that they had GA $\geq$ 37 weeks and matching birth dates and birth hospitals [24]. All the individuals who participated in the 5.5–year follow–up (182 cases and 125 controls) were invited for an MRI follow–up study and 74 cases and 69 controls complied. The participants and non–participants did not differ on any of the available measures: GA, BW, gender distribution, mothers' age at birth, mother's level of education or IQ scores measured on the follow–up at 5.5–years of age [24]. Furthermore, there were no statistically significant differences between the two groups of participants (case/control) with

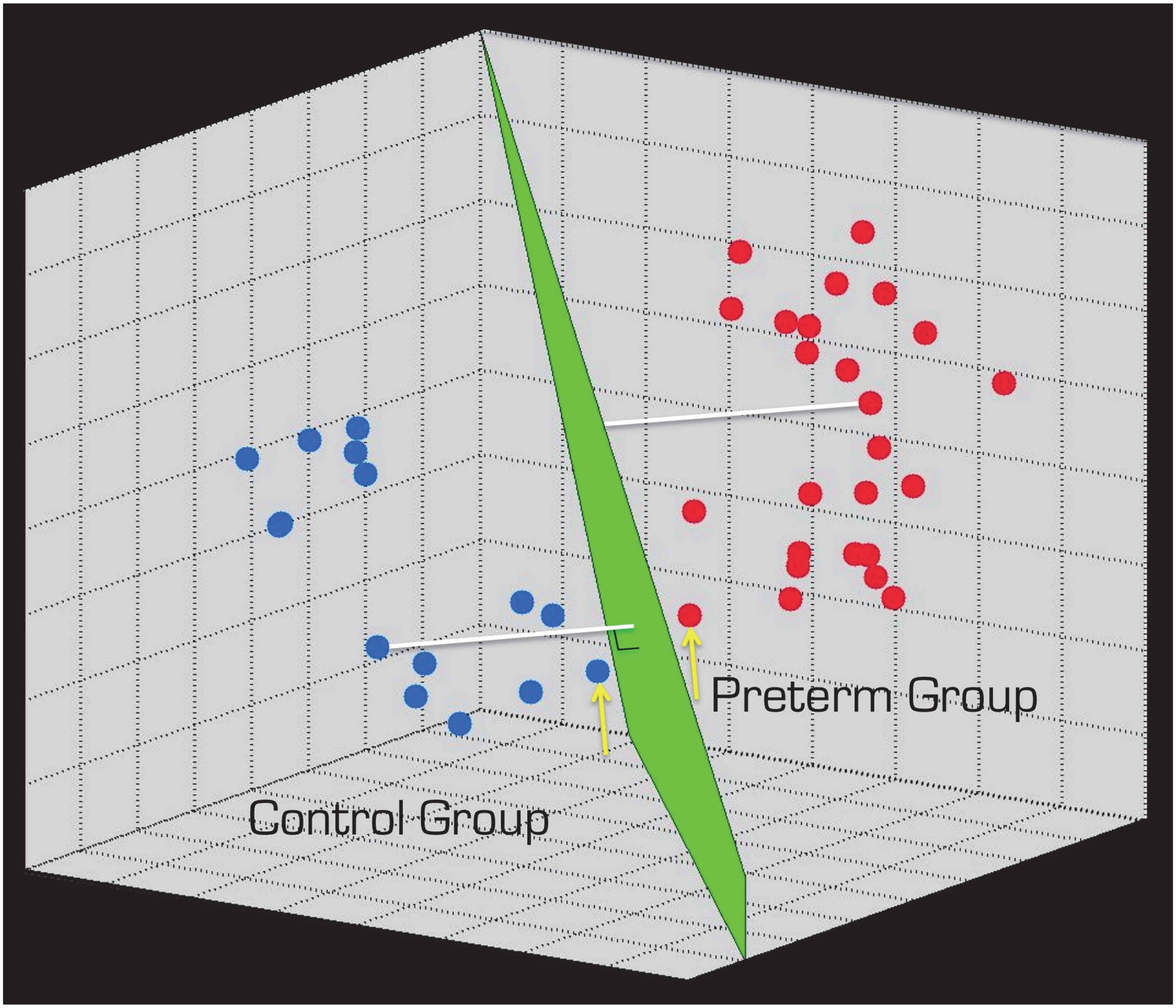

Fig 1. Illustrative representation of the SVM method. Although, SVMs work in multidimensional space where the dimensionality depends on the number voxels in the MR image, for simplicity and to aid the visual explanation, here only a 3D representation is shown. The filled blue and red circles represent hypothetical data from the two groups of interest. The green plane (which would be a called hyper plane in higher dimensions) between the clustered circles represents the decision boundary, consisting of points that maximize the distance (indicated by white lines) between the two groups. The most ambiguous points (i.e. closes to the plane) are called the 'support vectors' (indicated by yellow arrows).

doi:10.1371/journal.pone.0123108.g001

respect to weight/height at the time of scanning, gender distribution, mothers' age at birth or mothers' level of education [6]. The age of participants at the time of scanning ranged between 12.2 and 17.7 years (mean 14.6 years). For details please see Table 1.

All MRI data were collected at the Karolinska Hospital using a 1.5 T Signa scanner (General Electric, Waukesha, WI). The protocol included a T1–weighted gradient echo 3D anatomical image with echo time = 6 ms, repetition time = 24 ms, flip angle = 30° and voxel size 0.98 mm x 0.98 mm x 1.5 mm. Using the freely available SPM software (Wellcome Trust Centre for

Table 1. Description of the two groups compared in this study.

	Case Group	Control Group
**Number**	74 (51% girls)	69 (53% girls)
**Age (years)***	14.90 [12.38–17.7]	14.30 [12.18–16.47]
**Weight (kg)**	53.18. [25.6–83.8]	55.38 [32.4–88.9]
**Height (cm)**	163.94 [140.0–196.4]	165.60 [138.0–191.0]
**Gestational Age (weeks)**	28.54 [24–36]	39.72 [37–42]
**Birth Weight (g)**	1069.54 [645–1486]	3530 [2750–4655]
**Mother's age at birth (years)**	30.66 [20–42]	30.86 [22–44]

In the rows with Age and below the values are quoted as mean [range].

* The age quoted for those born preterm was not corrected for gestational age. If this correction is made the difference between the ages is not statistically significant

doi:10.1371/journal.pone.0123108.t001

Neuroimaging, London, UK) the T1–weighted images were segmented into gray matter (GM), white matter and cerebro-spinal fluid [25]. Using DARTEL [26] in SPM, which is a robust non–linear registration method [27], the GM segments were normalized to the common space of the mean GM segment of all the subjects, then subsequently modulated by the Jacobian determinants, re–sampled to 1.5 mm isotropic voxels and then smoothed isotropically using a filter with 6 mm at full width at half maximum. Subsequently, the above GM images were used, without feature selection or feature reduction, as input features to train a linear, hard margin SVM (http://www.csie.ntu.edu.tw/~cjlin/libsvm/) in Matlab (Mathworks, Natick, MA, USA). The groups of control and preterm born individuals were labeled -1 and +1 respectively [12]. Leave–one–out cross validation was applied to estimate accuracies [15]. The *prediction scores* (i.e. distance to the decision boundary including sign—see Fig 1), which is the dot–product between a test image and the weight map (Fig 2) plus the bias, were further used for correlation analyses. To assess the validity of results that the above leave–one–out procedure yielded, we made two additional training/classification cycles. In each case, we randomly selected 37 out of the 74 cases and 35 of the 69 controls to train the SVM. Subsequently the remaining, unseen individuals were given to the SVM for classification. In addition to the simple classification, receive operating characteristic (ROC) curves and the area under the ROC cureves (AUC) were calculated.

To obtain a non–parametric p value for the classification and the ROC/AUC, the above procedure was repeated 50000 times with the labels for the 143 participants randomly permuted. In a separate permutation sequence we repeated the procedure 2000 to generate a p value map.

Previous investigators reported a relationship between preterm birth and a reduced brain volume. Indeed we have confirmed this in a previous publication involving the same individuals [6]. Therefore, separate analyses were performed to examine how much of the variance is explained by total intracranial volume (TIV). Age and sex were also included as covariates to test the matching of the control and case groups. Residual forming matrix was used to efficiently remove the covariates [28]. To statistically compare TIV with the distance to decision boundary as a marker for preterm birth we used the DeLong test [29], which is specifically developed for correlated ROC curves.

Because the severity of outcome is inversely related to GA we made separate analyses in which the subjects born preterm were subdivided into 3 subgroups according to the definition of the World Health Organization. This stratification lead to 11 of the participants being moderately preterm (32 w $\leq$ GA $<$ 37 w), 36 participants being very preterm (28 w $\leq$ GA $<$ 32 w) and 27 participants being extremely preterm (GA $<$ 28 w). These 3 groups were designated as +1,

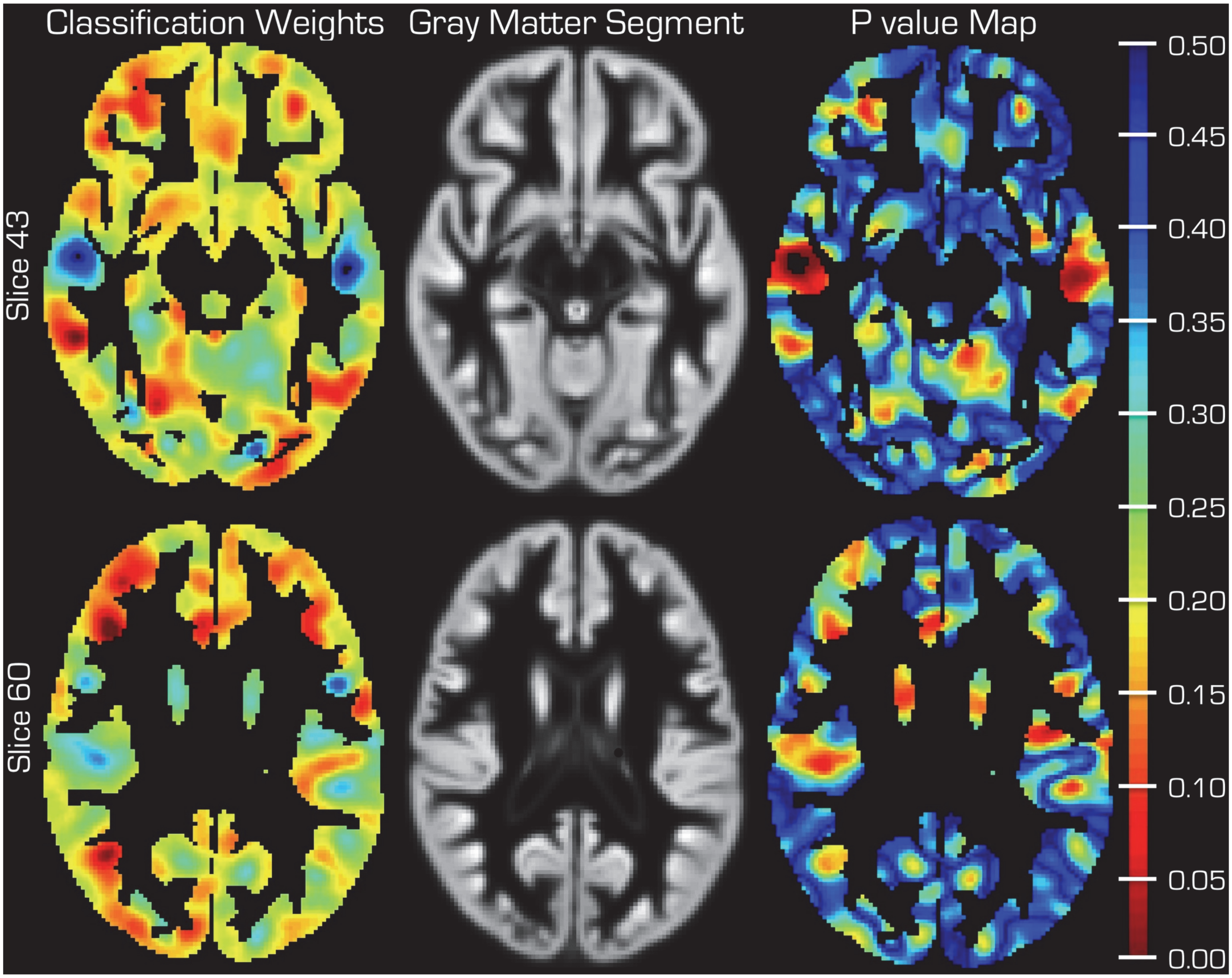

Fig 2. Gray Matter segment discriminative power for classification and p value map. Mean gray matter segment of all subjects is shown in the middle column for anatomical orientation. The color code on the images of the left column is in arbitrary units and indicates the weight (i.e. *discriminative power*) in each voxel. This value in each voxel is relative to the weight in all other voxels containing brain GM. The plots in the right column depict the corresponding p value maps. The color bar on the far right belongs only to the p value maps.

doi:10.1371/journal.pone.0123108.g002

+2 and +3 respectively. The additional analyses were performed between controls and Group +2 or Group +3 as well as between Group +2 and Group +3 to investigate whether the latter two groups (very preterm and extremely preterm) could be identified as two separate sub-groups. Another important factor is whether an individual was born smaller than his/her expected birth weight (i.e. born small for gestational age (SGA)). Following tables compiled by Fenton [30] we identified as SGA 16 (22%) out of the 74 preterm–born individuals. To test the possible confound where the SGA individuals, who may be more severely affected, may drive our results we performed an additional cycle of training and leave–one–out classification in which only the 58 individuals were included who were born with a weight appropriate for their GA.

Cognitive ability at 5.5 years of age was measured using the revised Swedish version of the Wechsler Preschool and Primary Scale of Intelligence Test as detailed in [24]. This test measures verbal IQ, performance IQ and full IQ. For 60 preterm–born individuals and 62 term–born individuals out of the 143 participants we had all 3 measures of IQ available for further analysis.

Using BW, GA and the different measures of IQ from 5.5 years of age [24] as dependent variables and the prediction score as a independent variable we quantified correlations by the Pearson's Correlation Coefficient. Note that, the higher the prediction score, the more likely the brain was from an individual that had been born preterm.

Results

We observed a high percentage of correct classifications (93%) from the leave–one–out cross validation. In other words, after training the SVM on the available data, it could correctly identify a person as belonging to the case or control group 93% of the time. This amounts to a sensitivity of 90.5% and a specificity of 94.2%. Different GM regions provide a varying weight toward the identification of brains (Fig 2). The corresponding ROC is plotted in Fig 3 (red curve) for which the AUC was 0.9755.

We assessed the reliability of above results by performing the leave–one–out procedure 50000 times. In the generated distribution of results the maximum accurate classification rate was 67% and the maximum AUC was 0.7240. The non–parametric p value for obtaining the original results of 93% accurate classifications and an AUC of 0.9755 was $p < 0.00002$.

After removing the effects of age and gender the original results were comparable (90% accurate classification). Including TIV as a covariate explained more variance, resulting in the reduction of correct classifications to 66%, which is still statistically significant ($p < 0.0011$). Because the correct classification did not reduce to chance (i.e. 50%) TIV is not the only significant discriminating factor of preterm birth. Indeed when we performed an SVM classification between the two groups based on the single feature of TIV the rate of accurate classifications was only 64%. The corresponding ROC is plotted in Fig 3 (blue curve) for which the AUC was 0.7012. Using the DeLong test [29] for correlated ROC curves we found that the improvement in predicting preterm birth with the distance to decision boundary is statistically significant ($p < 1.98 \times 10^{-10}$). In fact the 95% confidence interval for the AUC curve of the distance to decision boundary was 0.956–0.995.

In the two additional training/classification cycles where only a randomly selected half of the available individuals were used for training the SVM and the other half classified by the SVM the correct classification rates were 86% and 91%.

When testing the heterogeneity of the group of volunteers born preterm we found that the gestational age was indeed an important factor. Fig 4A depicts the results of the above SVM analysis on the entire group where the group of subjects born preterm is color–coded (blue = moderately preterm, green = very preterm, red = extremely preterm). The mean distances to the decision boundary for these 3 subgroups are 0.43, 0.73 and 1.20 respectively. The additional specific analyses performed between subgroups corroborated these findings. When including only the controls and the 27 individuals born extremely preterm (Group +3) the SVM classification was 100% accurate. This accuracy dropped to 90% when including only the controls and the 36 individuals born very preterm (Group +2). When directly comparing the 27 individuals born extremely preterm and the 36 individuals born very preterm directly the classification rate was 67% correct.

Fig 3B highlights each of the 16 individuals that had been born SGA. Their distance to decision boundary is distributed through the full range of the group of individuals born preterm,

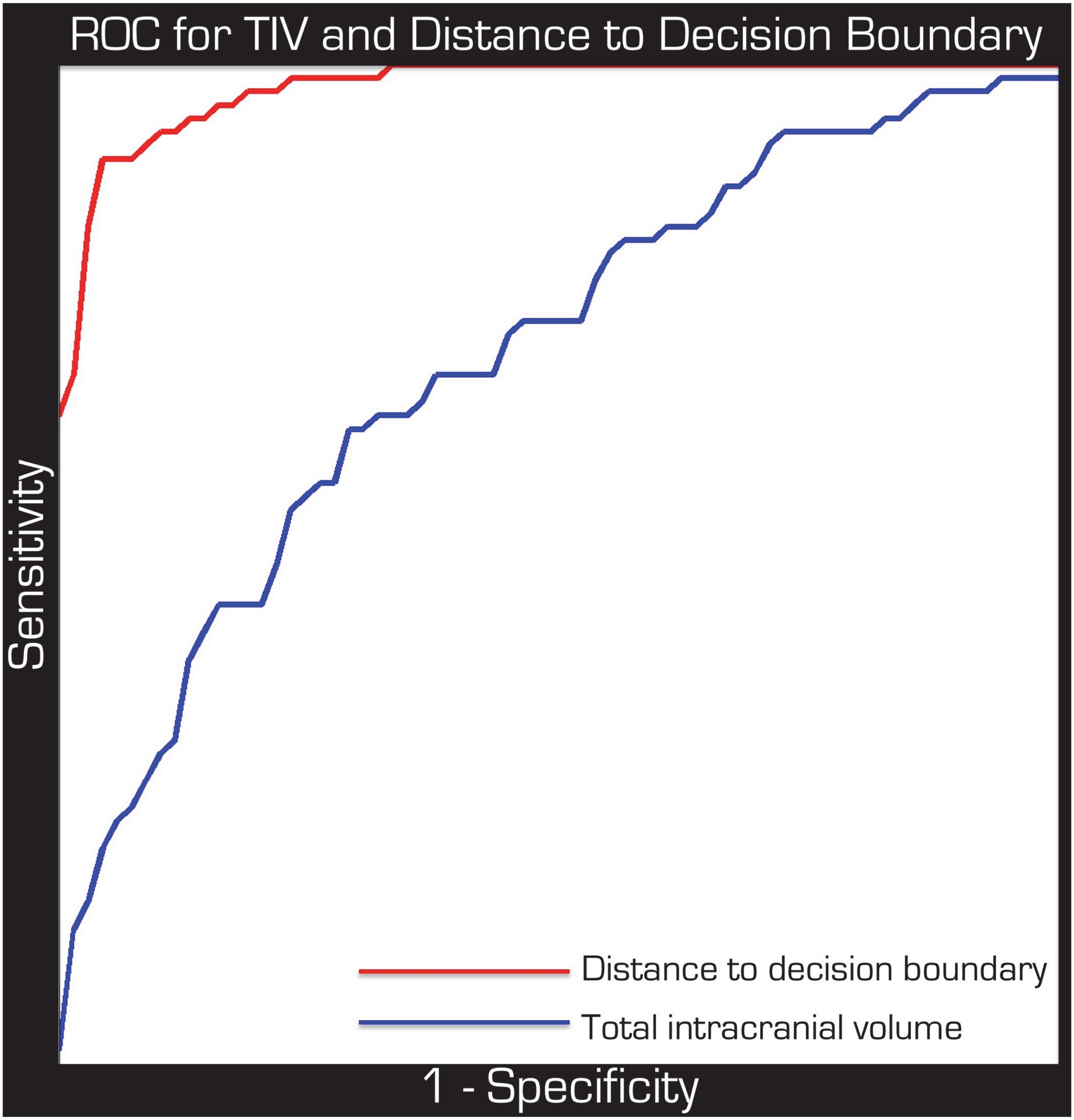

Fig 3. ROC curves for TIV and distance to decision boundary. Although these two measures are correlated markers for preterm birth the distance to decision boundary (red curve) is statistically significantly better. Both the x and y axes run between 0 and 1.

doi:10.1371/journal.pone.0123108.g003

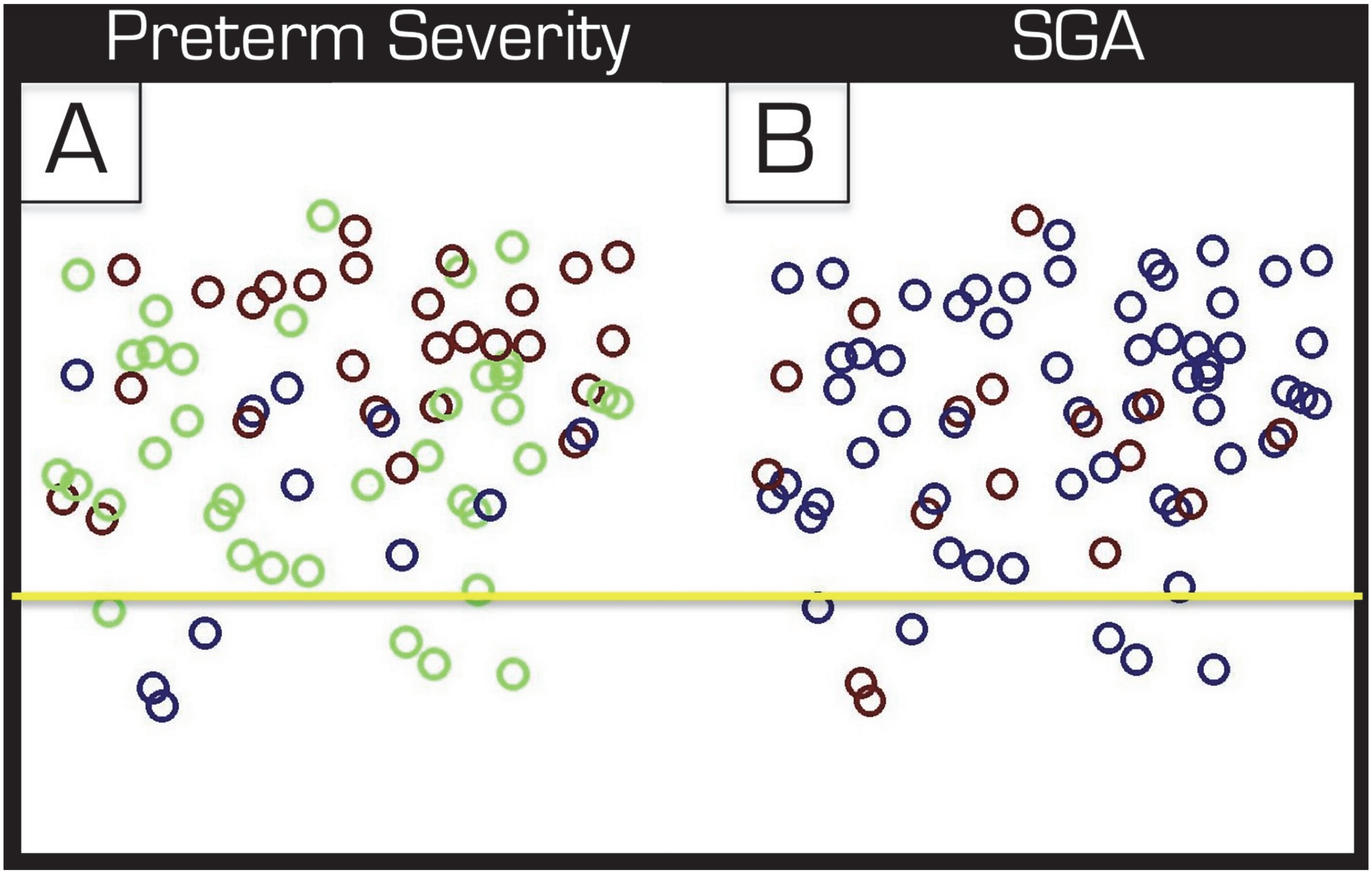

Fig 4. Effect of extent of preterm birth and SGA birth on results. The group of 74 participants that were born preterm is represented by colored circles. The vertical distance from the horizontal yellow line represents the distance to decision boundary. The further above the line is more like preterm whereas further below the line is more like control. In part A the color code represents the 11 moderately preterm (blue), 36 very preterm (green) and 27 extremely preterm (red) births based on gestational age. In part B the 16 individuals that were born SGA are represented by red circles while the other 58 by blue circles.

doi:10.1371/journal.pone.0123108.g004

which indicates that they are representative of group as a whole. This finding is corroborated by a separate analysis in which we compared the controls and the 58 individuals that were born with weights that were appropriate for their gestational age and 91% of the individuals were classified correctly by the SVM.

The correlation with the prediction score was stronger for BW (R = –0.51, $p < 0.000001$) than for GA (R = –0.24, $p < 0.0392$) in the group of preterm born individuals. As a check, BW was also correlated within the control group only but this did not yield statistically significant results (R = –0.13, $p < 0.2919$).

All measures of IQ were significantly correlated with the distance to decision boundary when both groups were considered: full IQ (R = –0.30, $p < 0.001$), performance IQ (R = –0.23, $p < 0.0133$) and verbal IQ (R = –0.29, p = 0.0012). Within only the group of individuals born preterm the correlation with Full IQ was also statistically significant (R = –0.34, $p < 0.0103$). Fig 5 depicts the prediction scores for both groups. Note that, while the mean predictive scores are clearly distinct for the two groups some individuals of each group possess scores, which could be representative of the other group. The color of the circles in Fig 5A represents the birth weight. For the subset of the group for which it was available, the IQ scores were used to

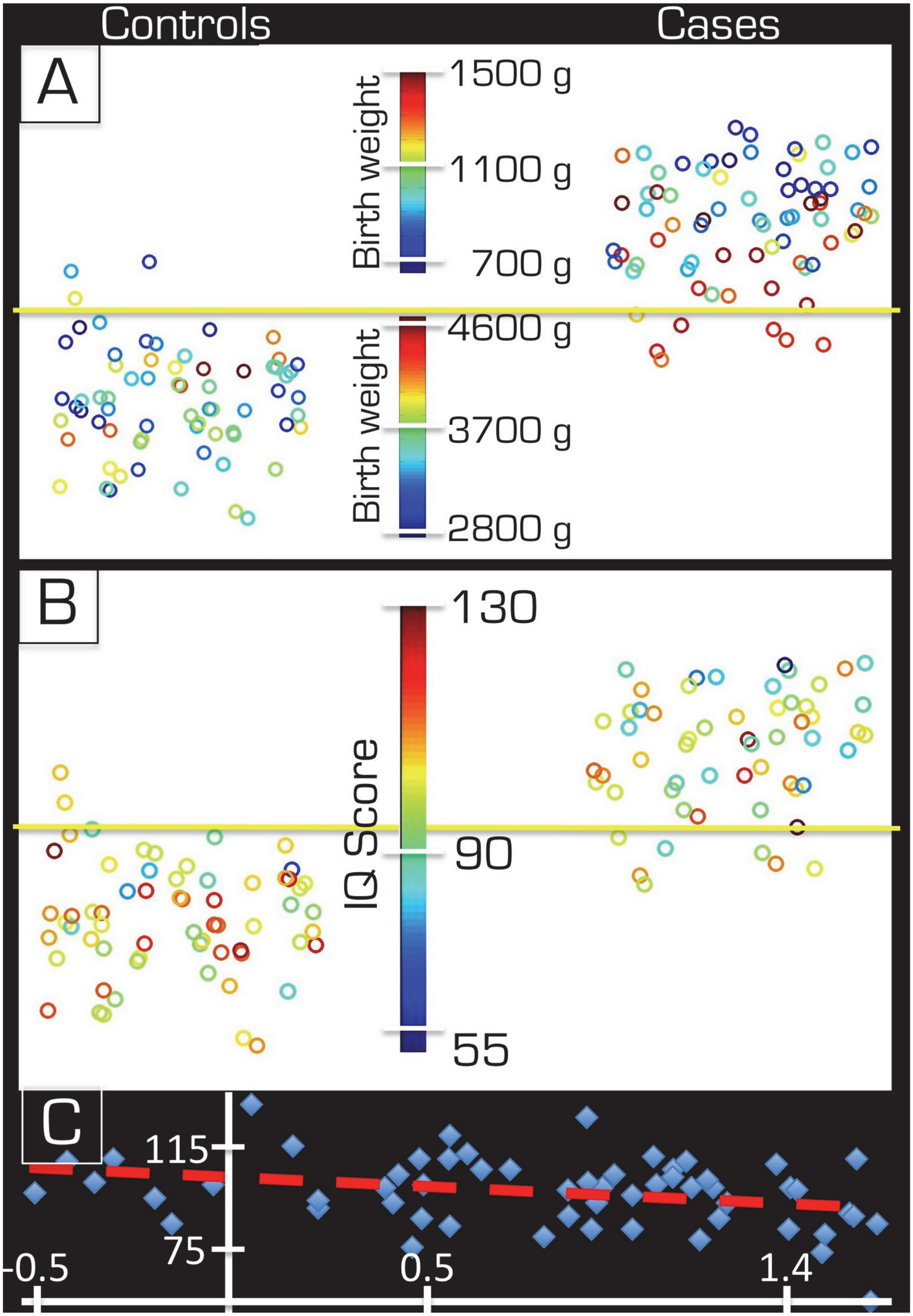
Controls
Cases
A
Birth weight
1500 g
1100 g
700 g
4600 g
3700 g
2800 g
B
IQ Score
130
90
55
C
115
75
-0.5
0.5
1.4

Fig 5. Distance to decision boundary in relation to BW and IQ. In parts A & B the distance to decision boundary (i.e. vertical distance relative to horizontal yellow line) is displayed for both groups. In part A, all 143 subjects are shown while in part B only those with IQ data are represented. The color code in part A indicates birth weight of the individual while on the bottom it represents the full IQ scores (see respective color bars in middle). In part C the distance to decision boundary (horizontal axis) and the full IQ score (vertical axis) are plotted against each other for the group of subjects born preterm. The red dashed line indicates the linear fit to the data ($R = -0.34$, $p < 0.0103$).

doi:10.1371/journal.pone.0123108.g005

color code the scatter plot in Fig 5B. While there is variability, the tendency of the high IQ scores of the control group and the low IQ scores of the case group were farther from the decision boundary. Fig 5C plots the distance to decision boundary on the x–axis against IQ on the y–axis. The 7 preterm–born individuals who fall below the horizontal decision boundary (horizontal yellow line) in Fig 5A and 5B are shown to the left of the vertical line in Fig 5C.

As with the classification results, TIV explains a large portion of the variance in the correlations. Still, after removing the effects of age, sex and TIV the correlation with full IQ ($R = -0.19$, $p < 0.038$) and verbal IQ ($R = -0.18$, $p < 0.042$) remain statistically significant.

Discussion

The success of the SVM in assigning a preterm–born or control individual to the correct group (93%), based on a single MR image, indicates that the distinct brain development of preterm–born individuals can be identified by machine–learning methods. Further, the fact that distance to the decision boundary correlates with variables of interest, such as IQ, GA and BW supports clinical significance of the method. The individuals born SGA were good representatives of the case group as a whole. Therefore it is unlikely that our results would have been driven by a subgroup of individuals who more severely effected.

There were limitations to this study. Although the cohort was very unique and the biographical information well maintained, the MRI images could be further improved with the latest MRI technology and more imaging time allocated. While it may be a limitation in this study, one would expect the rate of correct classifications to improve with higher quality input data. IQ data was not available from all individuals, but still 122 out of the 143 individuals could be evaluated in this manner. Furthermore, current work is under way to collect and analyze IQ data at 18 years of age and preliminary analysis indicates a high degree of correlation between IQ from 5.5 years of age and from late adolescence.

It is not surprising that TIV explains so much of the variance in the classification and correlation results. Extensive previous literature shows a correlation between preterm birth and a reduction in regional and global brain volume. However, it is important to point out that training an SVM using TIV alone cannot achieve the same accuracy in classifying the individuals as born preterm or at term.

Comparing individuals using the leave–one–out procedure inherently requires that a slightly different training sample is used each time (i.e. for each individual being left out). Thus, one may say that each result has its own meaning relative to that particular training sample and that due to these differences the 93% accurate classifications may be misleading. For this reason we performed the two additional training/classification cycles in which we used half the subjects in each group for training and classified the remaining individuals. This procedure yielded similar results to that of the leave–one–out method. It is also important to point out that if the individual left out is not part of the subset of support vectors the model will be identical even if the training sample was different. Therefore in those cases the comparison of distances to the decision boundary from different runs of the leave–one–out procedure is valid.

Inspection of Fig 2 indicates that different cortical areas provide varying weight toward the classification. Lower weight (blue) indicates relatively more GM in the healthy group and vice versa. In particular, anterior aspects of temporal lobe and lateral prefrontal cortex tend to have

a negative weight whereas the anterior and posterior poles provide more positive weights. Regions where those born at term have more GM coincide with the statistically significant regions we previously reported as being different on a group level [6].

In the case of individuals suffering from Alzheimer's disease, it has been found that an SVM trained on MRI data, which was collected at one center, could successfully identify individuals who underwent their MRI examination at a different center [20]. Further investigation is needed to establish whether such universal applicability could be achieved in the case of individuals born preterm. In the case of preterm birth, outcome can depend on local practices [31,32], and these practices can very among centers and countries [33]. Still there is a general consensus that preterm birth leads to a different developmental trajectory compared to that of term birth, and that this developmental trajectory is clearly identifiable. Therefore, while it may not be possible to train an SVM to universally identify a brain that belongs to an individual who was born preterm, SVMs trained on data at any center would be expected to perform well locally.

Employing a similar method, Franke et al. [18] used structural MRI brain images of a large number of children and adolescents and established a procedure for estimating the "brain age" of a given individual when compared against their training data. To test their proposed procedure they included a group of preterm–born adolescents and showed that, as one might expect, lower gestational age at birth resulted in a less "mature" brain when scanned in adolescence. We chose the SVM approach because it runs faster, makes fewer assumptions on the features being Gaussian distributed and requires a less involved image processing pipeline. It also must be pointed out that simply predicting the age of an individual may not correctly reflect their cognitive ability. To this end Erus et al. [34] made recent inquiries and we also aimed at finding correlations between classification confidence and other variables IQ.

Regardless of the method of classification, simple identification of a preterm–born individual as such is clearly not the final goal—even if the fact that it is done automatically is impressive. Rather, the ultimate aim of such methods would be to predict the outcome of a person who was born preterm. In other words, given that it is established that preterm–born individuals follow a distinct path in structural and functional brain development [1–8], the goal is to identify whether any given individual possesses a more preterm–like or a more term–like brain. The present study was a first step toward that goal, in that it investigated whether a *preterm brain* could be automatically distinguished from a *control brain* in the first place. The results indicate that indeed it can. There is recent evidence that SVMs trained on brain MR images can help prediction of future cognitive ability [22]. Indeed the prediction was more accurate when MR images were added to the classification than when based on cognitive tests alone. Future work will focus on similar investigations involving preterm–born individuals. Including SVM classification on MR images into the follow–up can similarly be expected to improve the prediction of outcome.

While the 93% correct classification is impressive, it remains to be seen whether it could be achieved with more heterogeneous groups or if additional data were given to the SVMs from other centers (i.e. data that was not used in the training and identification of the decision boundary between the two groups). On the other hand classifications can also be improved with more data. Here a single MRI image was used to train the SVM classifier, but further data, if available, could be included to more completely describe an individual. These additional data can be other MRI contrast, come from other imaging modalities, be based on demographic information and so on. Although it must be noted that using additional data in this way requires larger number of subjects to prevent over fitting the model.

Only 11 individuals were classified wrongly by the SVM. Their predictive score ranged between—0.49 and +0.67, compared to the range of the entire group (from—2.03 to +1.81). The IQ scores were similarly ambiguous for these individuals who were misclassified. Regardless of

whether preterm–born individual was classified as a control or the other way around, the mean IQ score for both of these cases was approximately 99. Due to the small number of misclassified subjects statistical comparisons are difficult to apply. The reason for misclassification may simply be random noise in the MRI images or movement–related artifacts, which hinder the ability of the SVM to make the correct classification.

Both BW and GA have been used previously as covariates and have been found to correlate with structural and functional outcome (see for example [35–38]). In the present study these variables of interest were found to correlate also with the distance to decision boundary. Interestingly however using BW as a covariate for only those individuals that were born at term the correlation was not significant, indicating specificity for preterm birth.

Even if SVMs correctly classify an individual with respect to group adherence or, better yet, a correct long–term prediction is given as to the structural development of the brain the significance of such findings remains an open question. The main outcome of interest is whether the structural differences that were identified couple with functional/cognitive ability or social skills and quality of life. To make an assessment of such outcome here IQ data were used. IQ is an important measure of outcome and has been shown to correlate with other measures (see for example [39,40]). While this measure correlated well with the distance to decision boundary (see bottom of Fig 5), which indicates a potential of the SVM methods in identifying clinical significance, it must be noted that IQ is but a single description of outcome. Further characterization of the significance of SVM classification will have to follow.

As opposed to voxel–based morphometry [41] analyses, which work very well for group comparisons, using SVM classifications has the added benefit of assigning a unique score to each individual (i.e. the distance to decision boundary). This score allows further stratification of the groups. Here we have shown that depending on the severity of preterm birth (moderate, very or extreme) the mean distance to decision boundary was progressively larger. In other words the individuals in these 3 subgroups were progressively more different from those in the control group. Further work is needed to see if one could subdivide the groups depending on an individual's distance to decision boundary and investigate the outcome of these subgroups.

In conclusion, SVM classification methods proved to be highly reliable in identifying individuals correctly, based on a single MRI image. Further work will focus on investigating how well these results generalize to data across centers and on what kind of improvements are needed, if any, to reach the end goal of predicting, on an individual basis, the specific outcome of persons born preterm.

Acknowledgments

The authors would like to thank Dr. Jonathan Clayden of University Collage London for kind assistance with performing the DeLong test.

Author Contributions

Conceived and designed the experiments: CC HL HF ZN. Performed the experiments: ZN. Analyzed the data: CC ZN. Wrote the paper: CC HL HF ZN.

References

1. Huppi PS, Maier SE, Peled S, Zientara GP, Barnes PD, Jolesz FA, et al. (1998) Microstructural development of human newborn cerebral white matter assessed in vivo by diffusion tensor magnetic resonance imaging. Pediatr Res 44(4):584–590. PMID: 9773850
2. Huppi PS, Murphy B, Maier SE, Zientara GP, Inder TE, Barnes PD, et al. (2001) Microstructural brain development after perinatal cerebral white matter injury assessed by diffusion tensor magnetic resonance imaging. Pediatrics 107(3):455–460. PMID: 11230582

3. Inder TE, Warfield SK, Wang H, Huppi PS, Volpe JJ. (2005) Abnormal cerebral structure is present at term in premature infants. Pediatrics 115(2):286–294. PMID: 15687434
4. Peterson BS, Vohr B, Staib LH, Cannistraci CJ, Dolberg A, Schneider KC, et al. (2000) Regional brain volume abnormalities and long-term cognitive outcome in preterm infants. JAMA 284(15):1939–1947. PMID: 11035890
5. Martinussen M, Fischl B, Larsson HB, Skranes J, Kulseng S, Vangberg TR, et al. (2005) Cerebral cortex thickness in 15-year-old adolescents with low birth weight measured by an automated MRI-based method. Brain 128(Pt 11):2588–2596.
6. Nagy Z, Ashburner J, Andersson J, Jbabdi S, Draganski B, Skare S, et al. (2009) Structural correlates of preterm birth in the adolescent brain. Pediatrics 124(5):e964. doi: 10.1542/peds.2008-3801 PMID: 19858152
7. Stewart AL, Rifkin L, Amess PN, Kirkbride V, Townsend JP, Miller DH, et al. (1999) Brain structure and neurocognitive and behavioural function in adolescents who were born very preterm. Lancet 353 (9165):1653–1657. PMID: 10335784
8. Hack M, Flannery DJ, Schluchter M, Cartar L, Borawski E, Klein N. (2002) Outcomes in young adulthood for very-low-birth-weight infants. N Engl J Med 346(3):149–157. PMID: 11796848
9. Skiöld B, Vollmer B, Böhm B, Hallberg B, Horsch S, Mosskin M, et al. (2012) Neonatal magnetic resonance imaging and outcome at age 30 months in extremely preterm infants. J Pediatr 160(4):559–566. e1. doi: 10.1016/j.jpeds.2011.09.053 PMID: 22056283
10. Counsell SJ, Shen Y, Boardman JP, Larkman DJ, Kapellou O, Ward P, et al. (2006) Axial and radial diffusivity in preterm infants who have diffuse white matter changes on magnetic resonance imaging at term-equivalent age. Pediatrics 117(2):376–386. PMID: 16452356
11. Krishnan ML, Dyet LE, Boardman JP, Kapellou O, Allsop JM, Cowan F, et al. (2007) Relationship between white matter apparent diffusion coefficients in preterm infants at term-equivalent age and developmental outcome at 2 years. Pediatrics 120(3):e604–e609. PMID: 17698966
12. Cristianini N & Shawe-Taylor J. (2000) An introduction to support vector machines and other kernel-based learning methods. Cambridge University Press.
13. Gaonkar B, Davatzikos C. (2013) Analytic estimation of statistical significance maps for support vector machine based multi-variate image analysis and classification. NeuroImage 78270–283. doi: 10.1016/j.neuroimage.2013.03.066 PMID: 23583748
14. Vapnik V (2000) The nature of statistical learning theory. Springer.
15. Fan Y, Shen D, Gur RC, Gur RE, Davatzikos C. (2007) COMPARE: classification of morphological patterns using adaptive regional elements. Medical Imaging, IEEE Transactions on 26(1):93–105. PMID: 17243588
16. Ameye L, De Brabanter J, K Suykens J, Cadron I, Devlieger R, Timmerman D, et al. (2005) Predictive models for long term survival after premature rupture of membranes. Conf Proc IEEE Eng Med Biol Soc 54622–4625. PMID: 17281270
17. Precup D, Robles-Rubio CA, Brown KA, Kanbar L, Kaczmarek J, Chawla S, et al. (2012) Prediction of extubation readiness in extreme preterm infants based on measures of cardiorespiratory variability. Conf Proc IEEE Eng Med Biol Soc 20125630–5633. doi: 10.1109/EMBC.2012.6347271 PMID: 23367206
18. Franke K, Luders E, May A, Wilke M, Gaser C. (2012) Brain maturation: predicting individual BrainAGE in children and adolescents using structural MRI. Neuroimage 63(3):1305–1312. doi: 10.1016/j.neuroimage.2012.08.001 PMID: 22902922
19. Kawasaki Y, Suzuki M, Kherif F, Takahashi T, Zhou SY, Nakamura K et al. (2007) Multivariate voxel-based morphometry successfully differentiates schizophrenia patients from healthy controls. Neuroimage 34(1):235–242. PMID: 17045492
20. Klöppel S, Stonnington CM, Chu C, Draganski B, Scahill RI, Rohrer JD, et al. (2008) Automatic classification of MR scans in Alzheimer's disease. Brain 131(Pt 3):681–689.
21. Mourão-Miranda J, Bokde AL, Born C, Hampel H, Stetter M, et al. (2005) Classifying brain states and determining the discriminating activation patterns: Support Vector Machine on functional MRI data. Neuroimage 28(4):980–995. PMID: 16275139
22. Ullman H, Almeida R, Klingberg T, et al. (2014) Structural maturation and brain activity predict future working memory capacity during childhood development. J Neurosci 34(5):1592–1598. doi: 10.1523/JNEUROSCI.0842-13.2014 PMID: 24478343
23. Katz-Salamon M, Forssberg H, Lagercrantz H, et al. (1997) The Stockholm Neonatal Project: Very Low Birthweight Infants in the Late 20th Century in Stockholm. Acta Paediatrica 86(Suplement 419):1–43.

24. Bohm B, Katz-Salamon M, Institute K, Smedler AC, Lagercrantz H, Forssberg H. (2002) Developmental risks and protective factors for influencing cognitive outcome at 5 1/2 years of age in very-low-birth-weight children. Dev Med Child Neurol 44(8):508–516. PMID: 12206615

25. Ashburner J, Friston KJ. (2005) Unified segmentation. Neuroimage 26(3):839–851. PMID: 15955494

26. Ashburner J. (2007) A fast diffeomorphic image registration algorithm. Neuroimage 38(1):95–113. PMID: 17761438

27. Klein A, Andersson J, Ardekani BA, Ashburner J, Avants B, Chiang MC, et al. (2009) Evaluation of 14 nonlinear deformation algorithms applied to human brain MRI registration. Neuroimage 46(3):786–802. doi: 10.1016/j.neuroimage.2008.12.037 PMID: 19195496

28. Chu C, Ni Y, Tan G, Saunders CJ, Ashburner J. (2011) Kernel regression for fMRI pattern prediction. Neuroimage 56(2):662–673. doi: 10.1016/j.neuroimage.2010.03.058 PMID: 20348000

29. DeLong E, DeLong DM, Clarke-Pearson DL (1988) Comparing the Areas Under Two or More Correlated Receiver Operating Characteristic Curves: A Nonparametric Approach. Biometrics 44:837–845. PMID: 3203132

30. Fenton TR. (2003) A new growth chart for preterm babies: Babson and Benda's chart updated with recent data and a new format. BMC Pediatr 313–13. PMID: 14678563

31. Gressens P, Rogido M, Paindaveine B, Sola A. (2002) The impact of neonatal intensive care practices on the developing brain. J Pediatr 140(6):646–653. PMID: 12072865

32. Murphy BP, Inder TE, Huppi PS, Warfield S, Zientara GP, Kikinis R, et al. (2001) Impaired cerebral cortical gray matter growth after treatment with dexamethasone for neonatal chronic lung disease. Pediatrics 107(2):217–221. PMID: 11158449

33. Van Reempts P, Gortner L, Milligan D, Cuttini M, Petrou S, Agostino R, et al. (2007) Characteristics of neonatal units that care for very preterm infants in Europe: results from the MOSAIC study. Pediatrics 120(4):e815–e825. PMID: 17908739

34. Erus G, Battapady H, Satterthwaite TD, Hakonarson H, Gur RE, Davatzikos C, et al. (2014) Imaging Patterns of Brain Development and their Relationship to Cognition. Cereb Cortex.

35. Nosarti C, Giouroukou E, Healy E, Rifkin L, Walshe M, Reichenberg A, et al. (2008) Grey and white matter distribution in very preterm adolescents mediates neurodevelopmental outcome. Brain 131(Pt 1):205–217.

36. McIntire DD, Bloom SL, Casey BM, Leveno KJ, et al. (1999) Birth weight in relation to morbidity and mortality among newborn infants. N Engl J Med 340(16):1234–1238. PMID: 10210706

37. Bhutta AT, Cleves MA, Casey PH, Cradock MM, Anand KJ. (2002) Cognitive and behavioral outcomes of school-aged children who were born preterm: a meta-analysis. JAMA 288(6):728–737. PMID: 12169077

38. Johnson S. (2007) Cognitive and behavioural outcomes following very preterm birth. Semin Fetal Neonatal Med 12(5):363–373. PMID: 17625996

39. Yung A, Poon G, Qiu DQ, Chu J, Lam B, Leung C, et al. (2007) White matter volume and anisotropy in preterm children: a pilot study of neurocognitive correlates. Pediatr Res 61(6):732–736. PMID: 17426647

40. Marlow N, Wolke D, Bracewell MA, Samara M. (2005) Neurologic and developmental disability at six years of age after extremely preterm birth. N Engl J Med 352(1):9–19. PMID: 15635108

41. Ashburner J, Friston KJ. (2000) Voxel-based morphometry—the methods. Neuroimage 11(6 Pt 1):805–821. PMID: 10860804
