# Supplementary material for: Correlation between white matter microstructure and executive functions suggests early developmental influence on long fibre tracts in preterm born adolescents
Source: PLoS One. 2017 Jun 8;12(6):e0178893. doi: 10.1371/journal.pone.0178893 (PMC5464584; doi:10.1371/journal.pone.0178893)
Supplement: S1 File — (ZIP) [file pone.0178893.s002.zip › New folder/Lundequist et al_2015.pdf]

ACTA PÆDIATRICA
NURTURING THE CHILD

Acta Pædiatrica ISSN 0803-5253

REGULAR ARTICLE

Cognitive outcome varies in adolescents born preterm, depending on gestational age, intrauterine growth and neonatal complications

Aiko Lundequist[1,2], Birgitta Böhm[2], Hugo Lagercrantz[2], Hans Forssberg[2], Ann-Charlotte Smedler (acsr@psychology.su.se)[1]

1.Department of Psychology, Stockholm University, Stockholm, Sweden
2.Department of Women's and Children's Health, Karolinska Institutet, Stockholm, Sweden

Keywords
Adolescents, Long-term cognitive outcome, Medical complications, Preterm birth, Small for gestational age

Correspondence
Ann-Charlotte Smedler, Department of Psychology, Stockholm University, Stockholm S-106 91, Sweden.
Tel: +46 (0)8 16 38 72 |
Fax: +46 (0)8 15 93 42 |
Email: acsr@psychology.su.se

Received
4 August 2014; revised 20 October 2014; accepted 10 November 2014.

DOI:10.1111/apa.12864

The copyright line for this article was changed on 18 June 2015 after original online publication.

ABSTRACT

Aim: The aim of this study was to investigate long-term cognitive outcome in a cohort of 18-year-olds born preterm and previously assessed at the age of 5.5.

Methods: We tested 134 adolescents born preterm with a very low birthweight of <1500 g and 94 term-born controls with a comprehensive cognitive battery at 18 years of age. The cohort was subdivided into 73 extremely preterm, 42 very preterm and 19 moderately preterm infants with gestational ages of 23–27, 28–31 and 32–36 weeks, respectively. The moderately preterm group was dominated by adolescents born small for gestational age.

Results: Very preterm adolescents performed on a par with term-born controls. In contrast, extremely preterm adolescents displayed inferior results on all cognitive tests, more so if they had suffered neonatal complications. Moderately preterm adolescents scored lower than very preterm and full-term born adolescents, particularly on complex cognitive tasks.

Conclusion: Adolescents born at 28 weeks of gestation or later, with appropriate birthweight and no perinatal complications, functioned like term-born peers at 18 years of age. Extremely preterm birth *per se* posed a risk for long-term cognitive deficits, particularly executive deficits. Adolescents born moderately preterm but small for gestational age were at risk of general cognitive deficits.

Epidemiological studies based on Scandinavian national registers have shown that adults born preterm tend to attain lower than average education and income and to have a higher degree of welfare support (1). Numerous follow-up studies in school-aged children have reported lower cognitive performance and more learning problems in preterm children (2,3), even among those born moderately preterm (4). Population-based longitudinal studies have shown that cognitive level in childhood, in particular with respect to executive functions, is related to social outcome in adulthood, as reflected by indices of health, wealth and public safety (5). It is reasonable to assume that a less successful social outcome in adults born preterm may be partially attributed to cognitive deficits.

Several factors are known to contribute to suboptimal cognitive development after preterm birth. The lower the gestational age at birth, the higher the risk of subsequent cognitive deficits and learning difficulties (6). The effect is partially mediated by neonatal complications, such as intraventricular haemorrhage, white matter disease, chronic lung disease and retinopathy of prematurity. These complications have been related to distinct neurodevelopmental disorders, such as cerebral palsy, and also to poor cognitive development in the absence of such disorders (7,8). Furthermore, large cohort studies (9,10) have shown that children born small for gestational age have learning problems and inferior school outcome, indicating that intrauterine growth restriction has a long-term negative impact on cognitive development.

Longitudinal studies of children born preterm indicate that the effect of preterm birth varies with age (11). For some children, the negative influence may become more obvious over time when increasing demands are put on the

Abbreviations
ANOVA, Analysis of variance; IQ, Intelligence quotient.

Key notes
- This study investigated long-term cognitive outcomes in a cohort of 18-year-olds born preterm and previously assessed at the age of 5.5.
- We found that adolescents who were born moderately preterm, but small for gestational age, faced a risk of general cognitive deficits.
- However, adolescents born after 28 weeks of gestation, with an appropriate birthweight and no perinatal complications, did not display an elevated risk for cognitive deficits.

©2014 The Authors. Acta Pædiatrica published by John Wiley & Sons Ltd on behalf of Foundation Acta Pædiatrica 2015 **104**, pp. 292–299
This is an open access article under the terms of the Creative Commons Attribution-NonCommercial-NoDerivs License, which permits use and distribution in any medium, provided the original work is properly cited, the use is non-commercial and no modifications or adaptations are made.

Table 1 Characteristics of the extremely preterm (EPT), very preterm (VPT), moderately preterm (MPT) and term-born control groups

	I<br>EPT (GA 23–27 weeks)<br>n = 73	II<br>VPT<br>(GA 28–31 weeks)<br>n = 42	III<br>MPT<br>(GA 32–36 weeks)<br>n = 19	IV<br>Term<br>(GA ≥ 37 weeks)<br>n = 94
Birthweight SDS*	−0.7 (0.99)	−1.9 (1.59)	−4.0 (1.5)	n.a.†
Neonatal medical complications‡	48%	8.3%	4%	0%
Males	47%	44%	52%	47%
Mothers age at child's birth§	31.9 (5.6)	32.4 (5.3)	32.3 (6.1)	30.9 (5.0)
Mother's education¶	4.2 (1.5)	3.9 (1.2)	4.3 (1.5)	4.0 (1.4)
Father's education¶	4.1 (1.6)	3.9 (1.4)	4.3 (1.5)	4.0 (1.4)

*Intrauterine growth expressed as Birth Weight Standard Deviation Score, BWSDS; Niklasson & Albertsson-Wikland, 2008.
†Term-born controls all had a BW > 2500 g.
‡Having one or more of the following neonatal medical complications: intraventricular haemorrhage, grades III–IV; periventricular leukomalacia, grades III–IV; chronic lung disease, grades 3+; retinopathy of prematurity, grades 3+.
§Expressed as mean (SD).
¶Parents' educational level, mean (SD) based on the classification used by Statistics Sweden (2000): 0 = no formal education, 1 = not finished elementary school, 2 = graduated from junior high school, 3 = completed 2 years of high school, 4 = completed 3 years of high school education, 5 = Bachelor's degree, 6 = Master's degree, 7 = Doctorate degree.

child, as indicated by higher needs for special education and lower school achievement (12). However, there are few follow-up studies on adolescents and young adults (10,13–15) and our knowledge about long-term cognitive outcomes after preterm birth is therefore still limited.

The study was conducted as part of the Stockholm Neonatal Project, a longitudinal population-based study of children born preterm in 1988–1993, with a very low birthweight of <1500 g who had been recruited at birth and followed prospectively (16). During the initial recruitment process, all children who met these criteria and were born in Karolinska Hospital or Löwenströmska Hospital in Stockholm were invited to take part. In addition, all children from the entire county of Stockholm with a birthweight of 1000 g or less who were in need of neonatal intensive care at Karolinska Hospital received an invitation. In this study, all preterm children (n = 182) and matched controls born at term (n = 125), who participated in a previous follow-up assessment at the age of 5.5, were invited to participate in a psychological assessment at the age of 18.

At 5.5 years of age, the preterm group had shown significantly lower results than the controls in visuo-motor, cognitive and executive function tests and their executive deficits were also significant when they were controlled for their intelligence quotient (IQ) (17). Low gestational age and intrauterine growth restriction late in pregnancy were associated with worse outcome, as were perinatal medical complications, particularly severe retinopathy of prematurity (7).

The aim of this study was to investigate cognitive outcome in late adolescence, to determine whether the pattern of cognitive deficits noted in the follow-up at the age of 5.5 had diminished or remained stable over time and if perinatal factors would still predict outcome in late adolescence.

METHODS

Participants

A total of 228 of the 306 (75%) invited adolescents from the original Stockholm Neonatal Project cohort accepted and participated in the study. Of the 78 families lost to follow-up, 12 had moved out of the Stockholm area and could not be located, 42 did not reply to our invitation, 23 declined participation, and one male born preterm was unable to complete the test protocol due to severe intellectual disability. The controls had been recruited in connection with the first comprehensive psychological follow-up of the Project cohort, at the age of 5.5 years. They consisted of healthy age-matched children born in the same hospital as the preterm children, at a full-term age of more than 37 weeks of gestation and with a birthweight of above 2500 g. In total, 134 preterm and 94 term-born controls completed the assessment at the age of 18. Our attrition analysis showed that there were no systematic differences between participants and dropouts in terms of gestational age, perinatal medical complications, developmental outcome at the age of 5.5 or paternal education. However, the mothers' educational level was lower among the dropouts in the preterm group ($p < 0.001$), and the same tendency was seen in the term group ($p = 0.09$).

For statistical analyses, the preterm cohort was divided into three subgroups according to gestational age: 73 extremely preterm infants with a gestational age of 23–27 weeks; 42 very preterm infants with a gestational age of 28–31 weeks and 19 moderately preterm infants with a gestational age of 32–36 weeks. The same subgrouping had been applied in the previous follow-up study (7). The extremely preterm group was the largest, because in the initial recruitment process, the catchment area was considerably greater for newborns weighing <1000 g. The moderately preterm group consisted of children with varying degrees of intrauterine growth restriction, since a very low

Table 2 The neuropsychological assessment battery

Variables	Measurement	Description
General intelligence (IQ)	The Wechsler Intelligence Scale for Children, 3rd ed. (WISC-III), short form	The test provides three IQ measures: full scale (FIQ), verbal (VIQ) and performance (PIQ) scores
Executive function	*Attention and speed* measured by Coding (W), Symbol search (W), Trail making 1 (D-KEFS).<br>*Working memory* measured by Digit span (W), Block repetition (WA)<br>*Cognitive flexibility* measured by Trail making 3, Verbal fluency, Design fluency, Colour-word 3, all from D-KEFS<br>*Complex executive function* measured by Sorting and Colour-word 4 from D-KEFS	(D-KEFS) = subtest from the Delis–Kaplan Executive Function Systems, yielding a scaled score with a mean of 10 ± 3.<br>(W) = subtest from WISC-III.<br>(WA) = subtest from Wechsler Adult Intelligence Scale Revised (WAIS-R)
Episodic memory, visuospatial	Face recognition	Examiner presents 24 pictures for 3 sec each. After 30 min, subjects are shown the 24 earlier presented faces, assembled together with 24 new faces, for 5 sec, for a forced yes–no recognition task
Episodic memory, verbal	Rey Auditory Verbal Learning test (RAVL), learning and retention	Examiner reads 15 words and subject repeats all the words that he/she can remember; five repeated trials. After 30 min delay, the participant is asked to again recall the words, rendering a retention score
Verbal function	Verbal Comprehension Index (VCI from WISC-III)<br>Proverb test (D-KEFS)<br>Boston naming test<br>Rapid naming test	Subtests Similarities and Vocabulary.<br>Interpret proverbs in own words.<br>60 line drawings of objects graded from easy to difficult/ unusual words.<br>Original test. Subject is asked to name 30 objects pictured on a chart as quickly as possible
Visuo-motor functions	Visuo-Motor Integration Test (VMI)<br>Trail making 1 (D-KEFS)	Copy designs<br>Draw a line between numbers

birthweight of <1500 g was an inclusion criterion when the prospective study started in 1988. In fact, 67% of subjects in the moderately preterm group were clinically classified as small for gestational age at birth. Characteristics of the study participants, including measures of birthweight, medical risk factors and parental education, are presented in Table 1.

Data collection

The participants were subject to an individual assessment at the age of 18 years ± 3 weeks. The assessment involved an extensive battery of cognitive tests, examining general intelligence, in the form of IQ, as well as episodic memory, verbal, visuo-motor and executive functions, with specific emphasis on the latter. The follow-up also included self-report measures of health and adjustment, interpersonal relations, school performance, interests and quality of life. The cognitive tests reported on here are listed in Table 2. The assessments took place at the Astrid Lindgren's Children's Hospital and were conducted by 15 Master level students in their last year of the clinical psychology programme at Stockholm University. The first author (AL) trained the students and analysed the test protocols and the senior psychologists (ACS and BB) supervised the testing and the analysis. Each examiner tested around 15 subjects, sampled from both the preterm and control groups. The examiners were blinded to which group the subjects belonged to. The tests were always administered in the same order, starting with the Wechsler Intelligence Scale for Children, 3rd edition, followed by block repetition and face recognition, and the Rey Auditory Verbal Learning tests. After a 20-min refreshment break, the subjects then underwent the Delis–Kaplan Executive Function System and the naming tests. The entire testing period lasted 3.5–4 h.

A number of neonatal complications were classified as involving high developmental risk. We found that 10 (7.5%) of the subjects had intraventricular haemorrhage grade III–V (18), 10 (7.5%) had periventricular leukomalacia grade III–IV (19), 17 (12.7%) had chronic lung disease grade III (20), and 21 (15.7%) had retinopathy of prematurity grade 3+ (21).

Statistical analysis

SPPS for Windows, version 18 (IBM, KIsta, Sweden), was used for the statistical analyses. To test for group differences, one-way analysis of variance (ANOVA) with post hoc tests was applied. *T*-tests were performed to compare cognitive outcomes between extremely preterm subjects with and without neonatal complications and to explore gender differences in cognitive outcome. To further investigate gender effects, two-way ANOVAs of IQ and a composite executive function measure were also conducted. To analyse the result of face recognition, d-prime (d') was

Table 3 Results, in group means and standard deviations, (a) on intelligence, verbal, visuo-motor and memory tests and (b) on tests of executive functions

	I: EPT (n = 73)	II: VPT (n = 42)	III: MPT/SGA (n = 19)	IV: Term (n = 94)	ANOVA F-value	Post hoc tests, significant subgroup differences, (effect size, Cohen's d)
(a)						
Intelligence						
Full Scale (FIQ)	84.3 (21.8)	97.5 (16.0)	81.6 (23.2)	97.2 (17.2)	9.7***	I & II (0.67)<br>I & IV (0.67)
Verbal (VIQ)	90.8 (18.3)	100.1 (13.6)	87.3 (18.3)	99.4 (14.8)	7.1***	I & II (0.57)<br>I & IV (0.52)
Performance (PIQ)	80.6 (23.9)	94.5 (18.8)	77.5 (22.0)	95.7 (20.0)	9.5***	I & II (0.63)<br>I & IV (0.70)
Verbal functions						
WISC VCI	96.9 (22.0)	104.7 (18.3)	89.3 (22.2)	101.9 (17.9)	3.5*	I & II (0.38)
D-KEFS Proverb	7.7 (4.1)	9.9 (2.7)	8.0 (4.0)	9.0 (3.5)	4.5*	I & II (0.60)
Boston naming	48.4 (5.9)	49.7 (5.2)	45.4 (7.5)	49.1 (4.8)	1.0	n.s.
Naming speed (Sec)	43.3 (17.5)	34.6 (9.6)	39.5 (11.1)	38.4 (13.5)	3.5*	I & II (0.58)
Visuo-motor functions						
Trail making 1	7.8 (3.7)	8.6 (2.8)	8.3 (2.7)	9.6 (2.9)	5.3**	I & IV (0.55)
VMI	19.4 (4.0)	20.8 (3.4)	20.0 (3.5)	21.9 (2.9)	7.5**	I & IV (0.74)
Episodic memory						
Face recognition d'	0.8 (0.54)	1.0 (0.60)	1.1 (0.56)	1.0 (0.55)	3.2*	I & II (0.36)<br>I & IV (0.37)
Rey verbal learning	51.9 (12.5)	58.2 (8.4)	56.1 (9.6)	56.8 (8.9)	4.3*	I & II (0.56)<br>I &IV (0.46)
Rey retention	11.6 (2.9)	12.2 (2.7)	12.6 (2.2)	12.0 (2.6)	0.92	n.s.
(b)						
Attention and speed						
Coding	59.0 (13.7)	62.9 (12.1)	58.0 (23.5)	66.2 (12.8)	5.2**	I & IV (0.56)
Symbol search	30.0 (7.7)	34.2 (6.3)	31.5 (6.8)	35.5 (5.5)	10.5***	I & II (0.58)<br>I & IV (0.83)
Working memory						
Digit span	13.6 (3.5)	15.2 (3.1)	14.1 (3.6)	15.4 (3.1)	4.9**	I & IV (0.55)
Block repetition	15.9 (3.2)	17.3 (3.0)	16.2 (3.2)	18.1 (2.8)	7.9***	I & IV (0.74)
Cognitive flexibility and inhibition						
Verbal fluency 3	8.5 (3.3)	11.0 (3.4)	9.9 (3.8)	11.7 (3.3)	9.8***	I & II (0.75)<br>I & IV (0.97)<br>III & IV (0.53)
Design fluency-T	9.8 (2.5)	11.6 (3.4)	9.5 (2.7)	11.1 (2.9)	6.3**	I & II (0.63)<br>I & IV (0.48)
Colour-word 3	7.1 (3.6)	9.7 (2.6)	7.2 (3.6)	9.5 (3.1)	10.0***	I & II (0.80)<br>I & IV (0.72)<br>III & IV (0.72)
Complex tasks						
Colour-word 4	7.3 (3.5)	9.9 (2.7)	7.2 (3.9)	9.5 (3.1)	9.8***	I & II (0.80)<br>I & IV (0.67)<br>III &IV (0.71)
Sorting	7.8 (3.4)	10.1 (2.3)	8.7 (3.1)	10.4 (2.9)	11.2***	I & II (0.75)<br>I & IV (0.83)<br>III & IV (0.58)

D-KEFS, Delis–Kaplan Executive Function System, test battery; EPT, extremely preterm; MPT/SGA, moderately preterm, small for gestational age; VMI, Visual Motor Integration test; VPT, very preterm; WISC VCI, verbal comprehension index from the Wechsler Intelligence Scale for Children.
*p < 0.05, **p < 0.01, ***p < 0.001.

used. Individual d' values were calculated based on the z-score for number of correct identification minus the z-score for false identifications, d′ = z (hits) − z (false) (22). The level of significance was set to 0.05. To avoid Type II errors, such as dismiss group differences of potential clinical relevance, we did not use Bonferroni corrections for multiple comparisons. Instead, statistical significance was complemented by calculation of effect sizes.

The longitudinal study was originally approved by the Ethics Committee at Karolinska Hospital (7). The collection of new data at 18 years of age and continued use of the original database was approved by the Regional Ethics

Board of Stockholm (2007/46-31/3). All participants gave their written consent prior to the data collection.

RESULTS

Results on tests of intelligence, verbal function, visuo-motor function and episodic memory by gestational age are shown in Table 3a and visually presented in Fig. S1a.

General intelligence

ANOVA showed significant results on all three IQ measures. Post hoc tests revealed that the extremely preterm group consistently scored lower than both the very preterm and the term group, with effect sizes in the upper medium range. The very preterm group performed similar to the term group, while the moderately preterm group performed at the same low level as the extremely preterm group. However, the moderately preterm group did not differ significantly from the very preterm and term groups, due to its small size (n = 19) and consequently limited statistical power.

Verbal function

ANOVA was significant for three of the four verbal tests and post hoc tests revealed significant differences between the extremely preterm and very preterm groups, with medium effect sizes (Table 3a). The moderately preterm group performed at the same low level as the extremely preterm group, but again these results were not statistically significantly different from the other groups.

Visuo-motor function

The extremely and moderately preterm groups performed below the level of the term group on both Trail Making and Visual Motor Integration (Fig. S1a). The results were statistically significant for the extremely preterm group, with a high medium effect size (Table 3a).

Episodic memory

ANOVA showed significant group differences on the face recognition and Rey learning tests (Table 3a) and post hoc tests revealed that the extremely preterm group performed significantly lower than the very preterm and term groups, on both tests. There were no group differences in Rey retention. The very preterm group and, more surprisingly, the moderately preterm group performed at the level of the term group on all three measures.

Executive functions

The results of the executive function tests are presented in Table 3b and illustrated in Fig. S1b. ANOVAs and post hoc tests showed that there were significant group differences on all executive function measures and that the extremely preterm group consistently performed significantly lower than the term group, with medium to large effect sizes (Table 3b). On several tests, the extremely preterm group also scored significantly below the very preterm group. The moderately preterm group performed significantly lower than the term group on several measures of cognitive flexibility/inhibition and complex tasks, with medium to large effect sizes.

Neonatal complications

The influence of neonatal complications on cognitive outcome in adolescents was only investigated in the extremely preterm group, in which 48% had suffered one or more severe neonatal complication as specified in the methods section, thus providing sufficient statistical power. Severe complications were relatively rare in the very preterm group (8%), whereas the moderately preterm group was dominated by children born small for gestational age with no additional complications. Test results of extremely preterm subjects who had experienced neonatal complications (n = 35) were compared to those without complications (n = 38), in a series of *t*-tests (Table S2). Significant group differences were found in general intelligence, measured as verbal IQ, performance IQ and full-scale IQ, in cognitive flexibility/inhibition and on complex executive function tasks, while no significant differences were found in attention/speed, working memory, episodic memory, verbal functions or visuo-motor functions.

Influence of gender

In the preterm group as a whole, females showed superior performance to males on several executive function tests, representing attention and speed, working memory and cognitive flexibility, on Rey verbal learning and retention and on visuo-motor tests (Table S1). Effects sizes were medium. In the term group, gender-related differences were found on just three tests and all in favour of females: Rey verbal learning and retention ($p > 0.001$), sorting ($p < 0.01$) and coding ($p < 0.05$).

To further explore the effect of gender, and possible gender by gestational group interaction effects, two-way ANOVAs were performed for the entire preterm group (n = 134), with full-scale IQ and a composite executive function index, respectively, as dependent variables, in two separate analyses. For full-scale IQ, there was no main effect of gender, but a significant gender by gestational group interaction effect ($F_{2,128} = 4.33$, $p < 0.02$). Extremely preterm females had a significantly higher IQ than extremely preterm males, whereas the inverse was true for the moderately preterm group. Notably, there were no significant gender differences in IQ in the very preterm group. The composite executive function index was calculated by adding together the z-scores of all the executive functions tests. For the executive index, there was, again, no main effect of gender, but a statistically significant gender by gestational group interaction effect ($F_{2,125} = 4.16$, $p = 0.02$), reflecting female superiority on the executive function index within the extremely and very preterm groups, whereas males outperformed females in the moderately preterm group.

DISCUSSION

In the present study, gestational age at birth was the one factor that had the largest impact on cognitive outcome in

adolescence. The extremely preterm group, which had a gestational age of 22–27 weeks, had the lowest results on all the cognitive tests, in particular on measures of executive functions. Indeed, this is something we also saw in the earlier follow-up study at the age of 5.5 (7) and the effect had not diminished but rather strengthened over time. The underlying brain mechanisms are not clear, although stress-induced pathways activated by the premature birth are likely to be a factor. Another partial explanation is provided by the neonatal complications that typically affect extremely preterm children. Despite this, the extremely preterm group without neonatal complications had a worse outcome than both the very preterm group and the control group born at term. This provides a strong indication that extremely preterm birth *per se* poses a risk for long-term cognitive development. Whatever the mechanisms that contribute to the suboptimal cognitive outcome, they also affect later brain development, including white matter integrity, as earlier reported in a diffusion tensor imaging study on a subsample of this cohort (23). The next scientific challenge will be to analyse the relation between executive functions on the one hand and alterations in brain structures and connectivity on the other.

A striking and encouraging finding in this study was that adolescents born very preterm, at a gestational age of 28–31 weeks, performed at the same level as term-born controls on all cognitive measures. Again, this is consistent with our earlier findings when the children were 5.5 years old, but in contrast to older follow-up studies of equally preterm born children, such as the Bavarian longitudinal studies (5). Although our results need to be replicated, one possible explanation for the positive outcome of our very preterm group may be the time factor, reflecting that continued improvements in neonatal medicine and care may have resulted not only in higher survival rates, but also in decreased morbidity and, in the end, better long-term outcome. Qualitative differences in the neonatal care between Stockholm and Bavaria could also be a factor, as well as important maternal health factors like a relatively low incidence of smoking and drug abuse among pregnant women in Sweden (24). The fact that all pregnant women in Sweden have access to high-quality maternity health care, free of charge, may be an important factor.

In contrast to the very preterm group, outcome in adolescence for the moderately preterm group, with a gestational age of 32–36 weeks, was lower than that of the term controls and this was significant when it came to the complex executive function tasks. This illustrates that poor nutrition of the foetus will negatively influence the developing brain, with lifelong impact on cognitive functions, and corresponds well to studies showing that adults born small for gestational age have significantly lower academic achievement and professional attainment (9). One possible mechanism is that maternal malnutrition leads to increased cortisol plasma levels in both the mother and the growth-retarded foetus (25), disturbing the hypothalamic–pituitary adrenal axis (26) and other brain circuits. The early foetal programming of the brain may also involve altered expression of the thyroid hormone transporter, which facilitates the entry of thyroid hormones across the blood–brain barrier and across the cell membrane into the neurons (27). The thyroid hormones are essential for proliferation, differentiation, migration, dendritic outgrowth and synaptogenesis, and minor alterations may already have critical effects on the development of neural circuits in the brain.

The cognitive profile of the moderately preterm infants was uneven. We hypothesise that this may reflect that disturbances caused by foetal starvation differentially affecting the neural circuits that underpin different cognitive functions. Given that, the fronto-striatal circuits, which are involved in complex executive functions, may be particularly vulnerable, while the hippocampal structures involved in episodic memory may be less affected.

In this study, male adolescents who had been born extremely or very preterm scored significantly lower results on several tests of executive functions, indicating that male gender remained a risk factor for unfavourable cognitive outcome at the age of 18. These results are consistent with a reported female advantage in educational attainment among adolescents born preterm (28). By contrast, in our moderately preterm group, which was dominated by adolescents born small for gestational age, males had a significantly better outcome than females. This, again, may reflect that different mechanisms are at play in the face of extremely preterm birth, as compared to foetal starvation later in pregnancy. However, our moderately preterm, small for gestational age group, was quite small (n = 19), and the suggested differential gender effect needs to be tested in future studies.

Intriguingly, verbal functions appeared less negatively affected by preterm birth than nonverbal and executive functions at the age of 5.5 years and this was still the case in late adolescence. Notably, one of our positive findings was that the adolescents born preterm performed on completely the same level as the controls in verbal retention and a few other studies have reported similar results (29). It is a common observation that language development tends to be comparatively robust, even in the face of cerebral lesions. It has been suggested that the ability to communicate by language is such a basic human ability, intrinsic to our social functioning, that it takes precedence when the brain's reserve capacity is taxed (30).

The strength of the present study is first and foremost that the preterm cohort was population-based, recruited at birth and had been followed prospectively. The control group was recruited at the age of 5.5 years and was demographically, geographically and socio-economically well matched to the preterm group. Attrition was seemingly unsystematic, moderate given the length of the follow-up and on the same level in the preterm and control groups. The psychological assessments were comprehensive and utilised tests that are frequently used in the clinic. Another strength is the fact that all the subjects were tested at essentially the same age, in the present studies this was 18 years of age plus or minus

3 weeks, by blinded examiners. Finally, due to the universally available and publicly funded health care for expectant mothers and young children in Sweden, socio-economic confounders are relatively weak and our cohort presented an opportunity to study outcome after preterm birth. The weaknesses of our study involved its relatively modest size and, consequently, limited statistical power for examining subgroups and specific risk factors.

We conclude that extremely preterm birth *per se* poses a risk for long-term cognitive outcome, particularly in executive functions. These risks seem to be exacerbated by neonatal complications, while female sex may be protective in the face of extremely or very preterm birth. Various risk factors differentially affect cognitive functions. Adolescents born small for gestational age but only moderately preterm, at 32 weeks or more, were likely to show cognitive deficits, particularly evident on tests of complex executive functions and intelligence. Reassuringly, our findings indicate that adolescents who were born at 28 weeks of gestation or later, with birthweights appropriate for gestational age and no severe neonatal complications, did not face elevated risk for long-term cognitive deficits.

ACKNOWLEDGEMENTS

We gratefully acknowledge financial support from the Swedish Research Council for Working Life and Social Sciences (2006-0936), Swedish Research Council (5925), Foundation Frimurarna Barnhuset, Vårdal Foundation (2007-B100) and Foundation Olle Engkvist Byggmästare, Sällskapet Barnavård.

References

1. Moster D, Lie RT, Markestad T. Long-term medical and social consequences of preterm birth. *N Engl J Med* 2008; 359: 262–73.
2. Litt J, Taylor HG, Klein N, Hack M. Learning disabilities in children with very low birthweight: prevalence, neuropsychological correlates, and educational interventions. *J Learn Disabil* 2005; 38: 130–41.
3. Taylor HG, Klein N, Minich NM, Hack M. Middle-school-age outcomes in children with very low birthweight. *Child Dev* 2000; 71: 1495–511.
4. Wolke D, Meyer R. Cognitive status, language attainment, and prereading skills of 6-year-old very preterm children and their peers: the Bavarian Longitudinal Study. *Dev Med Child Neurol* 1999; 41: 94–109.
5. Moffitt TE, Arseneault L, Belsky D, Dickson N, Hancox RJ, Harrington H, et al. A gradient of childhood self-control predicts health, wealth, and public safety. *Proc Natl Acad Sci* 2011; 108: 2693–8.
6. Hintz SR, Kendrick DE, Stoll BJ, Vohr BR, Fanaroff AA, Donovan EF, et al. Neurodevelopmental and growth outcomes of extremely low birth weight infants after necrotizing enterocolitis. *Pediatrics* 2005; 115: 696–703.
7. Böhm B, Katz-Salamon M, Smedler A-C, Lagrecrantz H, Forssberg H. Developmental risks and protective factors for influencing cognitive outcome at 5 1/2 years of age in very-low-birthweight children. *Dev Med Child Neurol* 2002; 44: 508–16.
8. Rose J, Butler EE, Lamont LE, Barnes PD. Neonatal brain structure on MRI and diffusion tensor imaging, sex, and neurodevelopment in very-low-birthweight preterm children. *Dev Med Child Neurol* 2009; 51: 526–35.
9. Strauss RS. Adult functional outcome of those born small for gestational age: twenty-six-year follow-up of the 1970 British Birth Cohort. *JAMA* 2000; 283: 625–32.
10. Pyhala R, Lahti J, Heinonen K, Pesonen AK, Strang-Karlsson S, Hovi P, et al. Neurocognitive abilities in young adults with very low birth weight. *Neurology* 2011; 77: 2052–60.
11. Hack M, Taylor HG, Drotar D, Schluchter M, Cartar L, Wilson-Costello D, et al. Poor predictive validity of the Bayley Scales of Infant Development for cognitive function of extremely low birth weight children at school age. *Pediatrics* 2005; 116: 333–41.
12. Nosarti C, Murray RM, Hack M, editors. *Neurodevelopmental outcomes of preterm birth: from childhood to adult life*. New York: Cambridge University Press, 2010.
13. Luu TM, Ment L, Allan W, Schneider K, Vohr BR. Executive and memory function in adolescents born very preterm. *Pediatrics* 2011; 127: e639–46.
14. Litt JS, Taylor GH, Margevicius S, Schluchter M, Andreias L, Hack M. Academic achievement of adolescents born with extremely low birth weight. *Acta Paediatr* 2012; 101: 1240–5.
15. Indredavik MS, Vik T, Evensen KA, Skranes J, Taraldsen G, Brubakk AM. Perinatal risk and psychiatric outcome in adolescents born preterm with very low birth weight or term small for gestational age. *J Dev Behav Pediatr* 2010; 31: 286–94.
16. Lagercrantz H, Katz-Salamon M, Forssberg H. The Stockholm Neonatal Project: neonatal mortality and morbidity at the Children's Centre, Karolinska Hospital. *Acta Paediatr Suppl* 1997; 419: 11–5.
17. Böhm B, Smedler A-C, Forssberg H. Impulse control, working memory and other executive functions in preterm children when starting school. *Acta Paediatr* 2004; 93: 1363–71.
18. Papile L-A, Burstein J, Burstein R, Koffler H. Incidence and evolution of subependymal and intraventricular hemmorrhage: a study of infants with birth weights less han 1500 gm. *J Pediatr* 1978; 92: 529–34.
19. Hesser U, Katz-Salamon M, Mortensson W, Flodmark O, Forssberg H. Diagnosis of intracranial lesions in very-low-birthweight infants by ultrasound: incidence and associations with potential risk factors. *Acta Paediatr Suppl* 1997; 419: 16–26.
20. Bancalari E, Abendour GE, Feller R, Gannon J. Bronchopulmonary dysplasia: clinical presentation. *J Paediatr* 1979; 95: 819–22.
21. Committee for the Classification of Retinopathy of Prematurity. An international classification of retinopathy of prematurity. *Arch Ophthalmol* 1987; 105: 905–12.
22. Wickens TD. *Elementary signal detection theory*. New York: Oxford University Press, 2002.
23. Nagy Z, Westerberg H, Skare S, Andersson JL, Lilja A, Flodmark O, et al. Preterm children have disturbances of white matter at 11 years of age as shown by diffusion tensor imaging. *Pediatr Res* 2003; 54: 672–9.
24. Gaddlin PO. Follow-up studies of very low birthweight children in Sweden. *Acta Paediatr* 2011; 100: 940–9.
25. Goland RS, Jozak S, Warren WB, Conwell IM, Stark RI, Tropper PJ. Elevated levels of umbilical cord plasma corticotropin-releasing hormone in growth-retarded fetuses. *J Clin Endocrinol Metab* 1993; 77: 1174–9.

 ©2014 The Authors. Acta Pædiatrica published by John Wiley & Sons Ltd on behalf of Foundation Acta Pædiatrica

26. Lesage J, Blondeau B, Grino M, Bréant B, Dupouy JP. Maternal undernutrition during late gestation induces fetal overexposure to glucocorticoids and intrauterine growth retardation, and disturbs the hypothalamo-pituitary adrenal axis in the newborn rat. *Endocrinology* 2001; 142: 1692–702.
27. Chan SY, Hancox LA, Martin-Santos A, Loubière LS, Walter MN, Gonzalez AM, et al. MCT8 expression in human fetal cerebral cortex is reduced in severe intrauterine growth restriction. *J Endocrinol* 2014; 220: 85–95.
28. Hack M, Flannery DJ, Schluchter M, Cartar L, Borawski E, Klein N. Outcomes in young adulthood for very-low-birth-weight infants. *N Engl J Med* 2002; 346: 149–57.
29. Schafer RJ, Lacadie C, Vohr B, Kessler R, Katz KH, Schneider KC, et al. Alterations in functional connectivity for language in prematurely born adolescents. *Brain* 2009; 132: 661–70.
30. Dennis M, Yeates KO, Taylor HG, Fletcher JM. Brain reserve capacity, cognitive reserve capacity, and age-based functional plasticity after congenital and acquired brain injury in children. In Stern Y, editor. "*Cognitive reserve. Theory and applications*". New York: Taylor & Francis, 2006: 58–83.

SUPPORTING INFORMATION

Additional Supporting Information may be found in the online version of this article:

Figure S1 (a) Mean z-scores[1] for tests of intelligence, episodic memory, verbal and visuo-motor fuctions for the extremely peterm group (EPT), very preterm gorup (VPT), and moderately preterm group (MPT) dominated by adolescents born SGA. (b) Mean z-scores[1] on D-KEFS tests of executive function for the for the extremely preterm group (EPT), very preterm gorup (VPT), and the moderately preterm group (MPT) dominated by adolescents born SGA.

Table S1 Statistically significant gender differences on cognitive measures among preterm and term-born adolescents, respectively.

Table S2 Comparison between extremely preterm (EPT) born adolescents with and without neonatal complications classified as high risk.
