# Supplementary material for: Correlation between white matter microstructure and executive functions suggests early developmental influence on long fibre tracts in preterm born adolescents
Source: PLoS One. 2017 Jun 8;12(6):e0178893. doi: 10.1371/journal.pone.0178893 (PMC5464584; doi:10.1371/journal.pone.0178893)
Supplement: S1 File — (ZIP) [file pone.0178893.s002.zip › New folder/Nagy et al_2009.pdf]

Structural Correlates of Preterm Birth in the Adolescent Brain

WHAT'S KNOWN ON THIS SUBJECT: A large number of studies identified preterm birth as a risk factor for long-term cognitive and structural brain development. However, it is known that developmental outcomes can differ on the basis of neonatal treatment and care.

WHAT THIS STUDY ADDS: This is one of the largest neuroimaging cohort studies investigating the long-term effects of preterm birth. Previous investigators did not combine structural brain images with DTI data. Furthermore, our findings are milder than those reported for similar cohorts.

abstract

OBJECTIVE: The Stockholm Neonatal Project involves a prospective, cross-sectional, population-based, cohort monitored for 12 to 17 years after birth; it was started with the aim of investigating the long-term structural correlates of preterm birth and comparing findings with reports on similar cohorts.

METHODS: High-resolution anatomic and diffusion tensor imaging data measuring diffusion in 30 directions were collected by using a 1.5-T MRI scanner. A total of 143 adolescents (12.18–17.7 years of age) participated in the study, including 74 formerly preterm infants with birth weights of ≤1500 g (range: 645–1486 g) and 69 term control subjects. The 2 groups were well matched with respect to demographic and socioeconomic data. The anatomic MRI data were used for calculation of total brain volumes and voxelwise comparison of gray matter (GM) volumes. The diffusion tensor imaging data were used for voxelwise comparison of white matter (WM) microstructural integrity.

RESULTS: The formerly preterm individuals possessed 8.8% smaller GM volume and 9.4% smaller WM volume. The GM and WM volumes of individuals depended on gestational age and birth weight. The reduction in GM could be attributed bilaterally to the temporal lobes, central, prefrontal, orbitofrontal, and parietal cortices, caudate nuclei, hippocampi, and thalami. Lower fractional anisotropy was observed in the posterior corpus callosum, fornix, and external capsules.

CONCLUSIONS: Although preterm birth was found to be a risk factor regarding long-term structural brain development, the outcome was milder than in previous reports. This may be attributable to differences in social structure and neonatal care practices. *Pediatrics* 2009;124: e964–e972

AUTHORS: Zoltan Nagy, MSc,[a,b] John Ashburner, PhD,[b] Jesper Andersson, PhD,[c] Saad Jbabdi, PhD,[d] Bogdan Draganski, MD,[b] Stefan Skare, PhD,[c] Birgitta Böhm, PhD,[e] Ann-Charlotte Smedler, PhD,[f] Hans Forssberg, MD, PhD,[e] and Hugo Lagercrantz, MD, PhD[a]

[a]Neonatal and [e]Neuropediatric Units, Department of Woman and Child Health, and [c]Magnetic Resonance Center, Department of Clinical Neuroscience, Karolinska Institute, Stockholm, Sweden; [b]Wellcome Trust Center for Neuroimaging, Institute of Neurology, University College London, London, England; [d]Centre for Functional Magnetic Resonance Imaging of the Brain, Department of Clinical Neurology, University of Oxford, Oxford, England; and [f]Department of Psychology, University of Stockholm, Stockholm, Sweden

KEY WORDS

preterm, magnetic resonance imaging, brain, diffusion tensor imaging, follow-up evaluation

ABBREVIATIONS

BW—birth weight
DTI—diffusion tensor imaging
WM—white matter
GM—gray matter
FA—fractional anisotropy
GA—gestational age
GMV—grey matter volume
SGA—small for gestational age
TBV—total brain volume
WMV—white matter volume

www.pediatrics.org/cgi/doi/10.1542/peds.2008-3801

doi:10.1542/peds.2008-3801

Accepted for publication Jun 4, 2009

Address correspondence to Zoltan Nagy, MSc, Department of Woman and Child Health, Karolinska Institute, Astrid Lindgren Children's Hospital, Q2:07, 171 76 Stockholm, Sweden. E-mail: zoltan.nagy@ki.se

PEDIATRICS (ISSN Numbers: Print, 0031-4005; Online, 1098-4275).

Copyright © 2009 by the American Academy of Pediatrics

FINANCIAL DISCLOSURE: *The authors have indicated they have no financial relationships relevant to this article to disclose.*

Downloaded from by guest on November 5, 2016

Many investigators have found that preterm birth affects structural development unfavorably. Children born preterm tend to achieve smaller brain volumes[1,2] and have greater tendencies for structural brain lesions, intraventricular hemorrhage, ventricular dilation,[3,4] and impaired growth of the corpus callosum.[5,6] They also possess a vulnerable white matter (WM) microstructure at term-equivalent age.[7,8]

However, it is now apparent that differences exist in neonatal care methods,[9] which might have significant effects on developmental outcomes.[10,11] The Stockholm Neonatal Project[12] was started to investigate how the long-term outcomes of individuals born preterm fit into the internationally reported spectrum. Therefore, it was designed as a prospective study of children born preterm during a 4.5-year period between 1988 and 1993. At 5.5 years of age, the formerly preterm children and their matched control subjects underwent neuropsychological assessments.[13,14]

In reports of similar adolescent cohorts, the investigators used morphometric analysis of either T1-weighted MRI structural images[15–17] or diffusion tensor imaging (DTI) data.[18–20] Interestingly, these 2 methods have not been combined before.

We expected that we would observe an association between preterm birth and structural brain development and that the gestational age (GA) and the birth weight (BW) would be inversely related to the severity of structural abnormality. Another aim of this study was to compare the outcomes with those of other cohorts cared for in other centers of the world.

TABLE 1 Description of the 2 Groups in This Study

	Case	Control
N	74	69
Female, %	51	53
Age, mean (range), y[a]	14.90 (12.38–17.7)	14.30 (12.18–16.47)
Weight, mean (range), kg	53.18 (25.60–83.80)	55.38 (32.40–88.90)
Height, mean (range), cm	163.94 (140.00–196.40)	165.60 (138.00–191.00)
GA, mean (range), wk	28.54 (24–36)	39.72 (37–42)
BW, mean (range), g	1069.54 (645–1486)	3530 (2750–4655)
Mother's age at birth, mean (range), y	30.66 (20–42)	30.86 (22–44)

The mothers' level of education is not shown because the scoring is on an ordinal scale and therefore mean values are not representative.

[a] The age quoted for subjects born preterm was not corrected for GA. If this correction is made, then the difference between the ages is not statistically significant.

METHODS

Subjects

Between September 1988 and March 1993, infants born at the Karolinska Hospital or transferred to the Karolinska Hospital from Löwenströmska Hospital in Stockholm were included if their BWs were $\leq$1500 g and their GAs were $\leq$36 weeks. In addition, all infants born in the entire county of Stockholm with BWs of $\leq$1000 g who received neonatal intensive care were invited to participate in the study.

Of the 291 infants who were included at birth, 55 died in the neonatal period or in early infancy, and 54 either had moved out of the Stockholm area or declined participation. At 5.5 years of age, 182 were available for follow-up evaluations.[13] At that time, a control group of 125 children with GAs of $\geq$37 weeks was recruited from a population-based register, according to birth date and birth hospital.[14]

All parts of this study were performed with the approval of the local ethics committee, and both the participant and an accompanying adult signed written consent forms. The 182 available case subjects and 125 control subjects were invited in random order, and 74 case subjects and 69 control subjects complied. The rest of the subjects either failed to respond to the invitation or declined. The participants did not differ from the original available group with respect to GA, BW, gender distribution, mother's age at birth, mother's level of education, or general cognitive development (verbal, performance, and full-scale IQ scores) at 5.5 years of age.[14] There was no statistically significant difference between the 2 groups with respect to weight and height at the time of scanning, gender distribution, mother's age at birth, or mother's level of education (Table 1).

MRI Data Collection

All participants underwent a cranial MRI examination at the Karolinska Hospital between April 2005 and February 2006, with a 1.5-T GE Signa Excite Twin-speed scanner (Waukesha, WI). All except 2 of the examinations were performed by Mr Nagy. The protocol (Table 2) included T1/T2-weighted MRI images and a DTI data set with 30 diffusion-weighted MRIs ($b = 1000$ seconds/mm^2) distributed evenly[21] and 4 reference images ($b = 0$ seconds/mm^2). The DTI data set was collected twice, with alternate-phase encoding directions to remove susceptibility-induced artifacts.[22] Although the T1-weighted MRI images were of good quality for all 143 subjects, the DTI images for 11 subjects (4 control subjects) were excluded from further analysis because of excessive movement artifacts.

Preprocessing of T1-Weighted MRI Scans

By using SPM 5 (Wellcome Trust Centre for Neuroimaging, London, England), a freely available software package, the

Downloaded from by guest on November 5, 2016

TABLE 2 Acquisition Parameters for T1-Weighted and DTI Data

Image Type	Mode	Sequence	Echo Time, ms	Repetition Time, ms	Flip Angle, °	Voxel Size, mm^3
T1-weighted	Three-dimensional	Gradient echo	6	24	30	0.98 × 0.98 × 1.5
T2-weighted	Two-dimensional	Turbo spin echo (ETL-12)	84	90	90	0.98 × 0.98 × 4.0
DTI	Two-dimensional	Twice-refocused spin echo	72	Triggered	90	1.96 × 1.96 × 3.0

Triggered indicates peripheral pulse triggering, with 1 to 3 slices collected per heartbeat, depending on heart rate.

T1-weighted MRI images were segmented into gray matter (GM), WM, and cerebrospinal fluid.[23] The GM and WM segments were warped to the space of the average of all of the subjects by using DARTEL.[24] The output images were modulated by the Jacobian determinants to preserve total volume irrespective of warping, resampled to 1.5-mm isotropic voxels, and smoothed with a 6-mm (full width at half-maximum), isotropic, Gaussian kernel before voxelwise statistical analysis. The total GM volume (GMV) and total WM volume (WMV) were estimated by integrating the GM and WM segment images, respectively. GMV and WMV were added to yield the total brain volume (TBV).

Statistical Analysis of T1-Weighted MRI Images

The GMV and WMV were compared between the groups by using 2-sample, 2-tailed *t* tests and assuming unequal variances and also were correlated with age at the time of scanning, BW, and GA. For voxel-based morphometry (VBM),[25] the first analysis was a simple comparison of the 2 groups by using voxelwise *t* tests. The second analysis included age, gender, and TBV as covariates. Because 16 of the formerly preterm subjects were born small for GA (SGA),[26] a separate analysis was performed with only the 58 individuals who were born with an appropriate BW.

Seventeen formerly preterm subjects (4 of them also SGA) and 4 control subjects had radiologic structural abnormalities (data not shown).[27] A final comparison was performed with only the 65 control subjects and 57 formerly preterm adolescents who had negative MRI findings. Within the group of formerly preterm adolescents, positive and negative correlations were made in a voxelwise manner by using GA or BW as a covariate.

In each of the analyses, correction was made for the large number of multiple comparisons. After the correction, *P* values of ≤.05 were considered statistically significant.

Preprocessing of DTI Data

The data sets for the 67 formerly preterm subjects and 65 control subjects with good-quality DTI data were corrected individually for eddy current artifacts and realigned.[28] The mean of the 4 reference images was used to estimate the extent of susceptibility-induced distortion.[22] The 2 data sets were then combined into a single undistorted series (Fig 1), from which the diffusion tensor and fractional anisotropy (FA) images[29] were calculated.

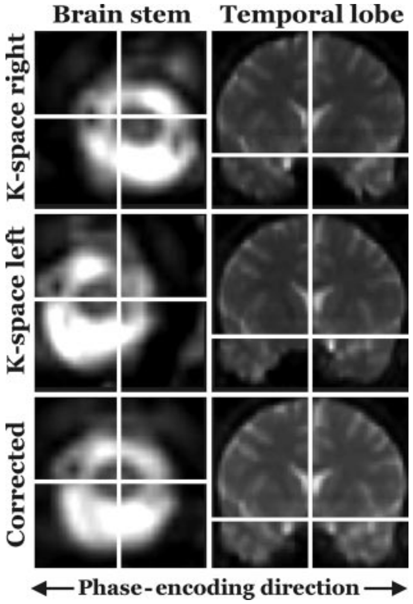

FIGURE 1
Illustration of the need for removal of susceptibility-induced distortions. The top 2 rows illustrate the original data sets, with the phase-encoding direction being left to right (ie, k-space is collected to the right) for the top row and right to left for the middle row. The bottom row is the corrected images that were used for the analyses. Left images are axial images at the level of the brainstem; right, coronal brain slices.

Statistical Analysis of FA Images

By using tools from the freely available FSL software package (Functional MRI of the Brain Centre, University of Oxford, Oxford, England), the tract-based, spatial, statistic pipeline was followed.[30] Nonbrain tissue was stripped from the mean reference image (brain extraction tool)[31] and was used to mask the FA image of the same subject. The FA images were aligned into a common space and resampled to 1-mm isotropic voxels (FMRIB's Non-linear Image Registration Tool). The mean FA image was thinned to create a mean FA skeleton, representing the centers of major WM tracks common to all of the subjects in the 2 groups. Finally, each of the FA images was projected onto this mean skeleton.

The statistical analyses were performed only with the set of voxels that belonged to the mean FA skeleton, with a minimal FA value of 0.2. Because the null distribution was not known and the images had not been smoothed, permutation methods (randomize tool) were used[32] on the cluster level.[33] Student's *t* tests were used to compare the groups, and correlations between variables of interest and the

Downloaded from by guest on November 5, 2016

voxelwise values were also performed. Although the skeleton is only 1 voxel thick, the displayed results are enlarged slightly to aid visualization.

Separate analyses were performed after exclusion of adolescents who were born SGA or demonstrated positive radiologic findings. *P* values of ≤.01 were considered statistically significant, after corrections for multiple comparisons had been made.

Statistical Analysis of Nonimage Data

For comparisons of the groups with respect to age, height, weight, and mother's age at birth, 2-tailed *t* tests were used, assuming unequal variances. A 2-sample test for binomial proportions was used to compare the gender distributions of the 2 groups. The mother's level of education was categorically compiled, with 0 = no schooling, 1 = 6 years of schooling, 2 = 9 years of schooling, 3 = <3 years of high school education, 4 = ≥3 years of high school education, 5 = <3 years of university education, 6 = ≥3 years of university education, and 7 = doctoral degree. For these data, the Wilcoxon rank-sum test (a nonparametric analog of a 2-sample *t* test) was used. In all cases, *P* values of <.05 were considered statistically significant.

RESULTS

Total GMV and WMV

The GMV (Fig 2A) and WMV (Fig 3A) were smaller in the group of adolescents born preterm, by 8.8% and 9.4%, respectively ($P < .001$). The GMV/WMV ratio did not differ statistically significantly between the 2 groups.

A statistically significant linear relationship emerged in which older subjects had smaller GMVs (Fig 2B); however, the slopes were not different between the groups. Correspondingly, WMV increased with the age of the subject, but this linear relationship was not statistically significant (Fig 3B).

Both GMV (Fig 2 C and D) and WMV (Fig 3 C and D) depended directly on BW and GA (more pronounced for WMV). These dependences were nonlinear and were strongest for adolescents who had been born most preterm and with the lowest BW. The GMV was independent of BW above ~1000 g,

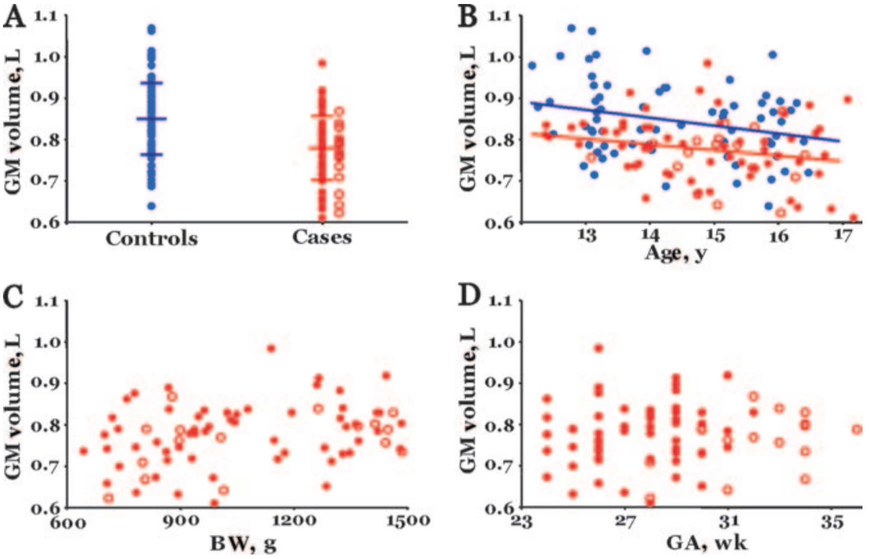

FIGURE 2
Plots of GMV versus preterm birth, age, GA, and BW. Red circles indicate adolescents born preterm (open circles, SGA); blue circles, term. A, Comparison of GMVs between the groups. Horizontal bars indicate means and SDs. B, GMV plotted against age at the time of scanning. The slope of the reduction in GMV versus age was statistically significant (control: $P = .016$; formerly preterm: $P = .039$), but there was no statistically significant difference between the slopes ($P = .596$). C, GMV plotted against BW for the formerly preterm adolescents. D, GMV plotted against GA for the group of formerly preterm adolescents.

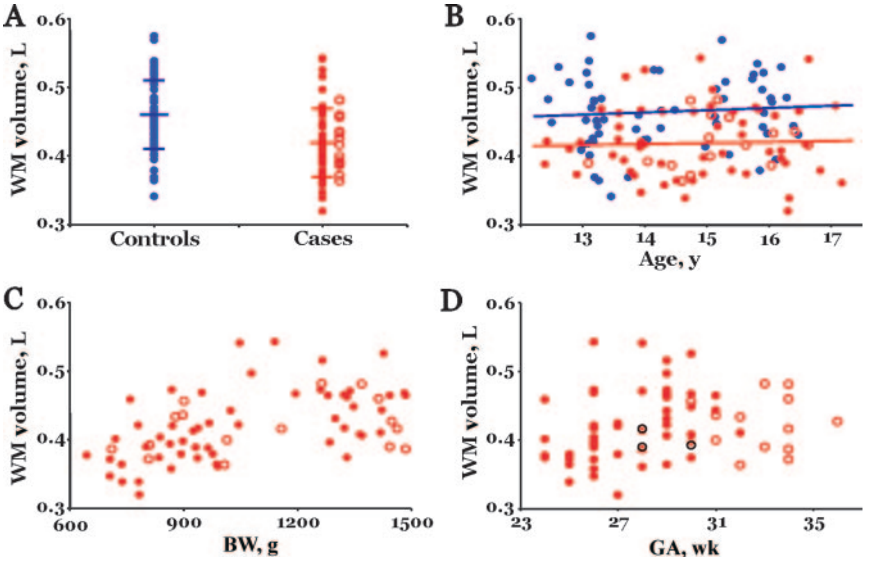

FIGURE 3
Plots of WMV versus preterm birth, age, GA, and BW. Red circles indicate adolescents born preterm (open circles, SGA); blue circles, term. A, Comparison of WMVs between the groups. Horizontal bars indicate means and SDs. B, WMV plotted against age at the time of scanning. The increase in WMV versus age was not statistically significant. C, WMV plotted against BW for the formerly preterm adolescents. D, WMV plotted against GA for the group of formerly preterm adolescents.

Downloaded from by guest on November 5, 2016

TABLE 3 MNI Coordinates for Voxels With Peak *z* and *t* Scores

Region	MNI Coordinates			*P*	*z* Score	*t*
	x	y	z			
Anterior temporal lobe, right	58.2	−11.8	−15.6	<.001	Inf	13.43
Posterior temporal lobe, right	57.0	−24.9	1.3	<.001	Inf	10.58
Anterior temporal lobe, left	−55.5	−9.8	−16.3	<.001	Inf	9.70
Posterior temporal lobe, left	−60.0	−25.0	−6.1	<.001	Inf	9.58
Parietal lobe, right	19.2	−73.7	35.1	<.001	6.17	6.62
Parietal lobe, left	−22.7	−70.7	31.5	<.001	7.78	8.74
Caudate nucleus, right	19.5	3.1	13.6	<.001	6.70	7.28
Caudate nucleus, left	−12.5	10.3	13.4	<.001	5.74	6.10
Hippocampus, right	25.0	−19.1	−19.2	<.001	6.90	7.54
Hippocampus, left	−22.4	−21.2	−19.5	<.001	6.39	6.89
Premotor cortex, right	52.6	4.2	13.8	<.001	Inf	8.89
Premotor cortex, left	−52	6.3	17.7	<.001	7.06	7.75
Orbitofrontal cortex, right	13.0	22.0	−23.8	<.01	5.70	6.06
Orbitofrontal cortex, left	−19.0	18.0	−16.1	<.001	6.10	6.53

The *P* value is the voxel-level statistic for the peak *t* and *z* scores. All of the mentioned regions were significant at the cluster level. MNI indicates Montreal Neurological Institute; Inf, infinity.

whereas the WMV even started to decrease in the range of 1000 to 1500 g. Both the GMV and WMV seemed to be independent of GA at >30 weeks, but there was a bias toward the children being SGA because of the inclusion criterion based on BW alone.

Voxelwise Comparison of GM Images

In comparisons of all of the 74 formerly preterm individuals with the 69 control subjects, the VBM analysis indicated a reduced grey matter volume in specific areas within all 4 lobes of the brain, the caudate nuclei, hippocampi, thalami all bilaterally. Interestingly, the cerebella were almost entirely unaffected (Table 3 and Figs 4 and 5). The converse analysis did not indicate any regions in which the control group had smaller GMV.

After correction for the effects of gender, age at the time of scanning, and TBV, the results became more focal, with involvement of only the temporal and parietal lobes (data not shown). Again, the converse analysis did not yield any regions in which the control group had smaller GMV.

Exclusion of the individuals who were born SGA did not have a noteworthy effect on the results. The major findings did not change significantly after exclusion of all individuals with abnormal radiologic findings.

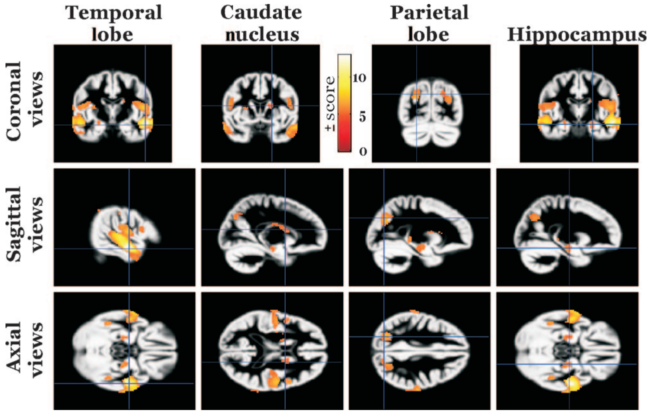

FIGURE 4
Results of the voxel-based morphometric analysis. The highlighted regions represent voxels where the GMV was on average higher for the group of control subjects than for the group of adolescents born preterm ($P < .05$, after correction for multiple comparisons). Most of the results show a striking bilaterality.

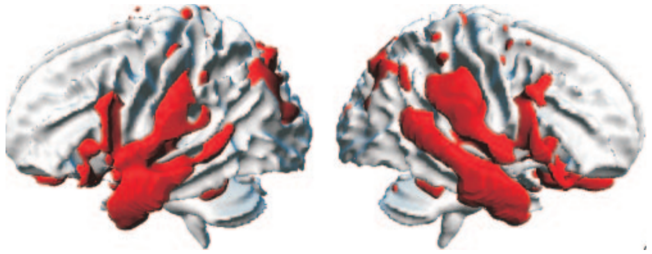

FIGURE 5
Surface-rendered display of the results of the voxel-based morphometric analysis. The results from Fig 4 (red surface) are overlaid on a volume-rendered depiction of the GM/WM boundary (white surface). Both hemispheres are shown to demonstrate the bilaterality of the findings ($P < .05$, after correction for multiple comparisons).

Only 2 focal regions of insignificant volume resulted from the analysis that correlated BW or GA with GMV in a voxelwise manner. The clusters were located in the left prefrontal and parietal cortices and consisted of 32 and 20 voxels, respectively.

Voxelwise Comparison of FA Data

Figure 6 displays the regions within WM tracts in which FA was higher in the control population than in group of formerly preterm adolescents. The affected areas included the external capsule bilaterally (potentially involving the uncinate fasciculus or the inferior frontooccipital fasciculus), the posterior body, isthmus, and anterior parts of the splenium of the corpus callosum, the forceps major (bilaterally), and the fornix (bilaterally). There was

Downloaded from by guest on November 5, 2016

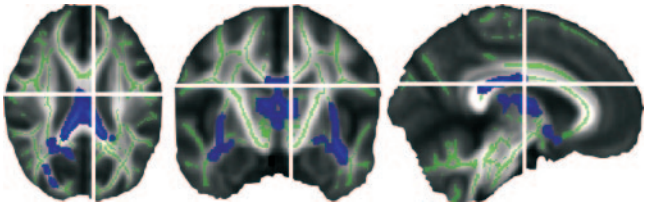

FIGURE 6
Regions of the brain in which FA is lower in adolescents born preterm. The voxels depicted in blue indicate WM tracts where FA is lower in formerly preterm adolescents than in matched control subjects ($P < .01$, after correction for multiple comparisons). These regions include the posterior aspects of the corpus callosum (body, isthmus, and splenium), the external capsules, and the fornix.

no significant cluster when the opposite contrast was tested.

FA correlated with BW and GA regardless of whether subjects were born preterm. Again, the external/extreme capsule, corpus callosum, and fornix were involved; however, when only the control population was considered, these covariations were not significant.

The results did not change significantly when all subject born SGA were excluded from the analysis. Exclusion of those with abnormal radiologic findings restricted the differences between the groups to only the external capsules.

DISCUSSION

By using state-of-the-art MRI acquisition and image-processing methods, we examined a large cohort and found that the total GMVs and WMVs were smaller in formerly preterm adolescents, compared with matched control subjects. Voxel-based morphometric measurement of the GM revealed that this difference was localized to the medial and superior temporal cortex, somatosensory cortex, parietal cortex, and subcortical GM structures. Track-based spatial statistics revealed correspondingly lower FA in the corpus callosum, external capsule, and fornix for formerly preterm adolescents. Furthermore, the GMV and WMV attained depended on both BW and GA, whereas voxelwise analyses revealed statistically significant correlations only between FA and BW/GA.

We found that formerly preterm adolescents had smaller GMV (8.8%) and WMV (9.4%), on average (Figs 2A and 3A). This finding is not unprecedented[1,2,34] and, given the common structural abnormalities in this group,[3,4,35] it was expected. With respect to the GMV, it is possible that, in response to the distressing extrauterine environment, endogenous corticosteroid levels were increased, which is known to inhibit growth.[11] However, the large overlap between the groups is notable (Figs 2A and 3A). Although the results were statistically significant, the biological or clinical significance may not be as alarming. For example, in a similar study, Nosarti et al[1] found a similar effect size (11.8%) in comparisons of GMV but no statistically significant difference in WMV.

In line with previous reports, the GMV decreased monotonically[36,37] while the WMV increased correspondingly[34,36] in both groups between 12 and 17 years of age, although the increase in WM was not statistically significant in this study. In our cohort, the GMV/WMV ratio did not differ between the 2 groups, which indicated that the brain as a whole, not only the GM or WM, developed differently.

The voxelwise comparison between the groups, without covariates, yielded extended regions in which the adolescents who were born preterm had smaller GMVs (Figs 5 and 6). These findings are in excellent agreement with the report by Peterson et al[2] and have commonalities with that by Nosarti et al.[15]

It is debatable whether the variability attributable to TBV should be included as a covariate. Although it is not always included,[15] most investigators remove the effects of TBV before presenting their results.[1,2,34] When such an extensive set of regions are involved, it is not surprising that regional differences covary with TBV. When we included TBV as a covariate, it explained most of the difference between the 2 groups and only the temporal and parietal lobe regions remained statistically significant, to a much reduced spatial extent, which was much less severe than that reported previously.[1,2,15,34]

Simple voxelwise comparison of the 2 groups indicated that FA was lower in the group of formerly preterm adolescents in the posterior part of the corpus callosum, the fornix, and the external capsules bilaterally. These findings are not unprecedented. Both Constable at al[20] and Vangberg et al[18] found similar results, at 12 and 15 years of age, respectively. It should be noted, however, that the extent of WM regions in which we could find statistically significant differences was much less.

The regions in which lower FA was detected generally connect cortical areas in which lower GMV was found. The body of the corpus callosum connects the bilateral motor and sensory cortices, the isthmus contains fibers connecting the parietal and temporal cortices as well as the parahippocampal gyrus, and axons in the splenium run mainly between the occipital cortices.[38,39] The external capsule contains fibers that run to the striatum, fibers in the fornix pass the hippocampi,[40] and the orbitofrontal and anterior

Downloaded from by guest on November 5, 2016

temporal areas are connected by the uncinate fasciculus.[41]

Although there is evidence that injured WM impairs the migration and proper development of neurons,[42,43] it is also known that electrical activity induced by neuronal activity is necessary for proper WM development.[44] With its observational design, the present study cannot unquestionably distinguish whether inferior WM connectivity led to the lower GMV or whether injury to the GM caused the reduction of FA in the corresponding WM fiber tracts.

Whether being born SGA has detrimental effects on brain and cognitive development is still debated. There are reports supporting both possibilities.[45–47] The differences in opinion may be a result of differences in the cohorts. This is not yet conclusive, however, because brain volumes alone may not always accurately reflect cognitive ability.[1,6] It should also be noted that, at 5.5 years of age, the IQ was significantly lower for the eldest GA group (32–36 weeks), of whom 63% were SGA. Our negative findings may be explained by catch-up development or may be a result of the small number of individuals ($n = 16$) in this subgroup.

In a separate analysis, we also excluded all individuals with positive radiologic diagnoses, to investigate whether the reductions in GMV or FA were apparent only in individuals with structural brain injuries. However, this affected only the FA analysis. This is probably because most of the identified injuries affected the WM, to a larger extent than injuries to the GM.

Overall, the differences between formerly preterm adolescents and appropriate control subjects were subtler than had been reported previously. This is in line with previous examinations and current radiologic findings for the same cohort. A large proportion of the infants were supported only with supplemental oxygen (25%) or nasal continuous positive airway pressure therapy (35%). Less than 25% of the infants were supported with mechanical ventilation only.[12] The neonatal mortality rate was low (17.5%), and corticosteroid prophylaxis and surfactant therapy were not yet routine (<10% of infants). A comprehensive neuropsychological assessment at 5.5 years of age revealed that the preterm group scored lower than the control subjects on the Wechsler Preschool and Primary Scale of Intelligence-Revised performance scale, which indicated deficits in visuospatial cognitive functioning, whereas no deficits were observed in the verbal domain.[14] Also, at 5.5 years of age, the preterm children tended to score lower on tests of executive functioning.[13] However, these observed deficits were minor and subclinical in nature. Structural abnormalities in this cohort were less frequent and less severe[27] than reported elsewhere.[47] Furthermore, in a recent report from our center,[48] structural brain abnormalities of neonates born preterm were less frequent than reported from other centers. We hypothesize that a combination of social structure and minimally invasive neonatal care may underlie this favorable outcome.

All of the case subjects ($N = 182$) and control subjects ($N = 125$) who participated in the follow-up evaluation at 5.5 years of age are presently (2007–2010) invited to participate in a psychological assessment at 18 years of age. The possible relationships between the morphologic findings in this study and the results of this psychological assessment will be investigated. Future plans also include tractography based on the DTI data, to elucidate whether the regions with lower FA values in formerly preterm individuals are parts of the fiber tracks connecting the areas where the cortex was found to be smaller.

ACKNOWLEDGMENTS

Funding support was provided by Sällskapet Barnavård (Sweden), the Swedish Research Council, the Bank of Sweden Tercentenary Foundation, and the Wellcome Trust (United Kingdom).

Many thanks go to Jessica Schiott for contacting and booking the subjects. Yords Österman and Marie Lundberg are experienced MRI radiographers who trained Mr Nagy extensively in data collection. Patient comfort and data quality were improved because of their involvement. Either Dr Anna-Karin Edstedt Bonamy (pediatrician) or Ms Jonna Karlen (medical student) attended each of the scans, to provide support in case of medical emergency and to assist with the scans. Their hard work and dedication were invaluable in the completion of this study. The late Dr David Freedman of the Department of Statistics, University of California, Berkeley, provided patient advice. Finally, the manuscript was much improved after Drs Geraint Rees and Chloe Hutton offered their assistance with the preparation.

REFERENCES

1. Nosarti C, Al Asady MH, Frangou S, Stewart AL, Rifkin L, Murray RM. Adolescents who were born very preterm have decreased brain volumes. *Brain*. 2002;125(7):1616–1623
2. Peterson BS, Vohr B, Staib LH, et al. Regional brain volume abnormalities and long-term cognitive outcome in preterm infants. *JAMA*. 2000;284(15):1939–1947
3. Dyet LE, Kennea N, Counsell SJ, et al. Natural history of brain lesions in extremely preterm infants studied with serial magnetic resonance imaging from birth and neurodevelopmental assessment. *Pediatrics*. 2006;118(2):536–548
4. Rutherford MA. *MRI of the Neonatal Brain*. London, England: Saunders; 2002
5. Anderson NG, Laurent I, Woodward LJ, Inder TE. Detection of impaired growth of the cor-

Downloaded from by guest on November 5, 2016

pus callosum in premature infants. *Pediatrics.* 2006;118(3):951–960
6. Stewart AL, Rifkin L, Amess PN, et al. Brain structure and neurocognitive and behavioural function in adolescents who were born very preterm. *Lancet.* 1999;353(9165):1653–1657
7. Hüppi PS, Maier SE, Peled S, et al. Microstructural development of human newborn cerebral white matter assessed in vivo by diffusion tensor magnetic resonance imaging. *Pediatr Res.* 1998;44(4):584–590
8. Anjari M, Srinivasan L, Allsop JM, et al. Diffusion tensor imaging with tract-based spatial statistics reveals local white matter abnormalities in preterm infants. *Neuroimage.* 2007;35(3):1021–1027
9. Van Reempts P, Gortner L, Milligan D, et al. Characteristics of neonatal units that care for very preterm infants in Europe: results from the MOSAIC study. *Pediatrics.* 2007;120(4). Available at: www.pediatrics.org/cgi/content/full/120/4/e815
10. Gressens P, Rogido M, Paindaveine B, Sola A. The impact of neonatal intensive care practices on the developing brain. *J Pediatr.* 2002;140(6):646–653
11. Murphy BP, Inder TE, Hüppi PS, et al. Impaired cerebral cortical gray matter growth after treatment with dexamethasone for neonatal chronic lung disease. *Pediatrics.* 2001;107(2):217–221
12. Katz-Salamon M, Forssberg H, Lagercrantz H. The Stockholm Neonatal Project: very low birthweight infants in the late 20th century in Stockholm. *Acta Paediatr.* 1997;86(suppl 419):1–43
13. Böhm B, Smedler AC, Forssberg H. Impulse control, working memory and other executive functions in preterm children when starting school. *Acta Paediatr.* 2004;93(10):1363–1371
14. Böhm B, Katz-Salamon M, Smedler AC, Lagercrantz H, Forssberg H. Developmental risks and protective factors for influencing cognitive outcome at 5 1/2 years of age in very-low-birthweight children. *Dev Med Child Neurol.* 2002;44(8):508–516
15. Nosarti C, Giouroukou E, Healy E, et al. Grey and white matter distribution in very preterm adolescents mediates neurodevelopmental outcome. *Brain.* 2008;131(1):205–217
16. Allin M, Henderson M, Suckling J, et al. Effects of very low birthweight on brain structure in adulthood. *Dev Med Child Neurol.* 2004;46(1):46–53
17. Martinussen M, Fischl B, Larsson HB, et al. Cerebral cortex thickness in 15-year-old adolescents with low birth weight measured by an automated MRI-based method. *Brain.* 2005;128(11):2588–2596
18. Vangberg TR, Skranes J, Dale AM, Martinussen M, Brubakk AM, Haraldseth O. Changes in white matter diffusion anisotropy in adolescents born prematurely. *Neuroimage.* 2006;32(4):1538–1548
19. Skranes J, Vangberg TR, Kulseng S, et al. Clinical findings and white matter abnormalities seen on diffusion tensor imaging in adolescents with very low birth weight. *Brain.* 2007;130(3):654–666
20. Constable RT, Ment LR, Vohr BR, et al. Prematurely born children demonstrate white matter microstructural differences at 12 years of age, relative to term control subjects: an investigation of group and gender effects. *Pediatrics.* 2008;121(2):306–316
21. Jones DK, Horsfield MA, Simmons A. Optimal strategies for measuring diffusion in anisotropic systems by magnetic resonance imaging. *Magn Reson Med.* 1999;42(3):515–525
22. Andersson JL, Skare S, Ashburner J. How to correct susceptibility distortions in spin-echo echo-planar images: application to diffusion tensor imaging. *Neuroimage.* 2003;20(2):870–888
23. Ashburner J, Friston KJ. Unified segmentation. *Neuroimage.* 2005;26(3):839–851
24. Ashburner J. A fast diffeomorphic image registration algorithm. *Neuroimage.* 2007;38(1):95–113
25. Ashburner J, Friston KJ. Voxel-based morphometry: the methods. *Neuroimage.* 2000;11(6):805–821
26. Fenton TR. A new growth chart for preterm babies: Babson and Benda's chart updated with recent data and a new format. *BMC Pediatr.* 2003;3:13
27. Nagy Z, Jonsson B. Cerebral MRI findings in a cohort of ex-preterm and control adolescents. *Acta Paediatr.* 2009;98(6):996–1001
28. Andersson JL, Skare S. A model-based method for retrospective correction of geometric distortions in diffusion-weighted EPI. *Neuroimage.* 2002;16(1):177–199
29. Basser PJ, Pierpaoli C. Microstructural and physiological features of tissues elucidated by quantitative-diffusion-tensor MRI. *J Magn Reson B.* 1996;111(3):209–219
30. Smith SM, Jenkinson M, Johansen-Berg H, et al. Tract-based spatial statistics: voxelwise analysis of multi-subject diffusion data. *Neuroimage.* 2006;31(4):1487–1505
31. Smith SM. Fast robust automated brain extraction. *Hum Brain Mapp.* 2002;17(3):143–155
32. Nichols TE, Holmes AP. Nonparametric permutation tests for functional neuroimaging: a primer with examples. *Hum Brain Mapp.* 2002;15(1):1–25
33. Hayasaka S, Nichols TE. Validating cluster size inference: random field and permutation methods. *Neuroimage.* 2003;20(4):2343–2356
34. Reiss AL, Abrams MT, Singer HS, Ross JL, Denckla MB. Brain development, gender and IQ in children: a volumetric imaging study. *Brain.* 1996;119(5):1763–1774
35. Skranes J, Evensen KI, Løhaugen GC, et al. Abnormal cerebral MRI findings and neuroimpairments in very low birth weight (VLBW) adolescents. *Eur J Paediatr Neurol.* 2008;12(4):273–283
36. Giedd JN, Blumenthal J, Jeffries NO, et al. Brain development during childhood and adolescence: a longitudinal MRI study. *Nat Neurosci.* 1999;2(10):861–863
37. Sowell ER, Peterson BS, Thompson PM, Welcome SE, Henkenius AL, Toga AW. Mapping cortical change across the human life span. *Nat Neurosci.* 2003;6(3):309–315
38. Sunderland S. The distribution of commissural fibres in the corpus callosum in the macaque monkey. *J Neurol Psychiatry.* 1940;3(1):9–18
39. Moses P, Courchesne E, Stiles J, Trauner D, Egaas B, Edwards E. Regional size reduction in the human corpus callosum following pre- and perinatal brain injury. *Cereb Cortex.* 2000;10(12):1200–1210
40. Poletti CE, Creswell G. Fornix system efferent projections in the squirrel monkey: an experimental degeneration study. *J Comp Neurol.* 1977;175(1):101–128
41. Petrides M, Pandya DN. Association fiber pathways to the frontal cortex from the superior temporal region in the rhesus monkey. *J Comp Neurol.* 1988;273(1):52–66
42. Inder TE, Hüppi PS, Warfield S, et al. Periventricular white matter injury in the premature infant is followed by reduced cerebral cortical gray matter volume at term. *Ann Neurol.* 1999;46(5):755–760
43. Leviton A, Gressens P. Neuronal damage accompanies perinatal white-matter damage. *Trends Neurosci.* 2007;30(9):473–478
44. Demerens C, Stankoff B, Logak M, et al. Induction of myelination in the central nervous system by electrical activity. *Proc Natl Acad Sci U S A.* 1996;93(18):9887–9892
45. Tolsa CB, Zimine S, Warfield SK, et al. Early alteration of structural and functional brain development in premature infants born with intrauterine growth restriction. *Pediatr Res.* 2004;56(1):132–138
46. Ranke MB, Vollmer B, Traunecker R, et al.

Downloaded from by guest on November 5, 2016

Growth and development are similar in VLBW children born appropriate and small for gestational age: an interim report on 97 preschool children. *J Pediatr Endocrinol Metab*. 2007;20(9):1017–1026

47. Skranes JS, Martinussen M, Smevik O, et al. Cerebral MRI findings in very-low-birth-weight and small-for-gestational-age children at 15 years of age. *Pediatr Radiol*. 2005;35(8):758–765

48. Horsch S, Hallberg B, Leifsdottir K, et al. Brain abnormalities in extremely low gestational age infants: a Swedish population based MRI study. *Acta Paediatr*. 2007;96(7):979–984

Downloaded from by guest on November 5, 2016

Structural Correlates of Preterm Birth in the Adolescent Brain
Zoltan Nagy, John Ashburner, Jesper Andersson, Saad Jbabdi, Bogdan Draganski, Stefan Skare, Birgitta Böhm, Ann-Charlotte Smedler, Hans Forssberg and Hugo Lagercrantz
Pediatrics 2009;124;e964; originally published online October 26, 2009;
DOI: 10.1542/peds.2008-3801

**Updated Information & Services**	including high resolution figures, can be found at: /content/124/5/e964.full.html
**References**	This article cites 45 articles, 11 of which can be accessed free at: /content/124/5/e964.full.html#ref-list-1
**Citations**	This article has been cited by 15 HighWire-hosted articles: /content/124/5/e964.full.html#related-urls
**Subspecialty Collections**	This article, along with others on similar topics, appears in the following collection(s): **Neurology** /cgi/collection/neurology_sub
**Permissions & Licensing**	Information about reproducing this article in parts (figures, tables) or in its entirety can be found online at: /site/misc/Permissions.xhtml
**Reprints**	Information about ordering reprints can be found online: /site/misc/reprints.xhtml

PEDIATRICS is the official journal of the American Academy of Pediatrics. A monthly publication, it has been published continuously since 1948. PEDIATRICS is owned, published, and trademarked by the American Academy of Pediatrics, 141 Northwest Point Boulevard, Elk Grove Village, Illinois, 60007. Copyright © 2009 by the American Academy of Pediatrics. All rights reserved. Print ISSN: 0031-4005. Online ISSN: 1098-4275.

American Academy of Pediatrics
DEDICATED TO THE HEALTH OF ALL CHILDREN™

Downloaded from by guest on November 5, 2016

PEDIATRICS®
OFFICIAL JOURNAL OF THE AMERICAN ACADEMY OF PEDIATRICS

Structural Correlates of Preterm Birth in the Adolescent Brain

Zoltan Nagy, John Ashburner, Jesper Andersson, Saad Jbabdi, Bogdan Draganski, Stefan Skare, Birgitta Böhm, Ann-Charlotte Smedler, Hans Forssberg and Hugo Lagercrantz

Pediatrics 2009;124;e964; originally published online October 26, 2009;
DOI: 10.1542/peds.2008-3801

The online version of this article, along with updated information and services, is located on the World Wide Web at:
/content/124/5/e964.full.html

PEDIATRICS is the official journal of the American Academy of Pediatrics. A monthly publication, it has been published continuously since 1948. PEDIATRICS is owned, published, and trademarked by the American Academy of Pediatrics, 141 Northwest Point Boulevard, Elk Grove Village, Illinois, 60007. Copyright © 2009 by the American Academy of Pediatrics. All rights reserved. Print ISSN: 0031-4005. Online ISSN: 1098-4275.

American Academy of Pediatrics
DEDICATED TO THE HEALTH OF ALL CHILDREN™

Downloaded from by guest on November 5, 2016
