# Supplementary material for: Correlation between white matter microstructure and executive functions suggests early developmental influence on long fibre tracts in preterm born adolescents
Source: PLoS One. 2017 Jun 8;12(6):e0178893. doi: 10.1371/journal.pone.0178893 (PMC5464584; doi:10.1371/journal.pone.0178893)
Supplement: S1 File — (ZIP) [file pone.0178893.s002.zip › New folder/Nagy_Jonsson_2009.pdf]

ACTA PÆDIATRICA
PROMOTING CHILD HEALTH

Acta Pædiatrica ISSN 0803–5253

REGULAR ARTICLE

Cerebral MRI findings in a cohort of ex-preterm and control adolescents

Zoltan Nagy (zoltan.nagy@ki.se), Baldvin Jónsson

Department of Woman and Child Health, Neonatology, Karolinska Institute, Stockholm, Sweden

Keywords
Brain injury, Cohort, Follow-up, MRI, Preterm birth

Correspondence
Zoltan Nagy, Department of Woman and Child Health, Neonatology, Astrid Lindgren Children's Hospital Q2:07, Stockholm 171 76, Sweden.
Tel: +46-8-51777354 |
Fax: +46-8-51777353 |
Email: zoltan.nagy@ki.se

Received
19 November 2008; revised 17 February 2009; accepted 23 February 2009.

DOI:10.1111/j.1651-2227.2009.01278.x

Abstract

Aim: Newborn infants were entered between 1988 and 1993 into a prospective, long-term, follow-up study. We aimed to investigate how the outcome of preterm-born individuals on cerebral magnetic resonance imaging (MRI) compared to that reported on similar cohorts internationally.
Methods: The 74 ex-preterm (12.38–17.7 years, 51% girls) and 69 control participants (12.18–16.47 years, 53% girls) underwent a MRI examination on a 1.5T scanner. Two experienced neuroradiologists examined the T1- and T2-weigthed images first independently and then in consensus without knowledge of group adherence.
Results: Only 21 (4 controls) of the 143 sets of scans showed any abnormalities. All but one of these were of mild extent. Among the ex-preterm adolescents two showed only incidental findings while the other 15 had either gliosis or white matter loss. Eleven subjects had white matter loss, seven of which had no other abnormalities. Four subjects had gliosis, three of which had no other abnormalities. The extent, severity or frequency of injury was not related to being born small for gestational age.

Conclusion: Although the rate of structural abnormalities was higher in the group of adolescents born preterm, this rate was well below that reported from other centres around the world. We attribute this to the minimally invasive neonatal care and to different social structures in Sweden compared to that of other reports on similar cohorts.

INTRODUCTION

The basis for investigating the long-term effects of preterm birth is not only scientific but contains an ethical aspect as well. Cerebral findings and neurodevelopmental outcome of individuals who are born too early has become an area of intense research. Previous investigators report that children born preterm tend to have more structural brain lesions, intra-ventricular haemorrhage (IVH) and ventricular dilatation (1,2); possess a vulnerable white matter microstructure which can already be detected at term-equivalent age (3); achieve smaller brain volumes (4) and impaired growth of the corpus callosum (5); may have impaired cerebral vasculature (6); demonstrate suboptimal cognitive ability starting from early childhood (7), through later childhood (8,9) and adolescence (5) and even into young adulthood (10), to mention but a few relevant aspects.

Given the differences that exist between neonatal care practices (11) and the effect they can have on the outcome of the individual (12–14) it is important to examine the outcome of individuals at many settings. The Stockholm Neonatal Project (15) is a prospective study of preterm birth and was started in 1988 with the aim to examine how the outcome of graduates of the minimally invasive Scandinavian neonatal care fit into the internationally reported spectrum.

Previous investigators who compared magnetic resonance imaging (MRI) and histology found a good correspondence in normal development and in cases of haemorrhage or infarction (16). The process of normal or disordered myelination can also be followed well with this technique (17). The available spatial resolution and the ease of prescribing the imaging orientation also allow studies into the development of the cortex (18), germinal matrix and cortical folding (19). Another advantage of MRI is the different contrasts it offers. The predictive value of MRI findings and their correlation with normal development, motor and cognitive function or gender have been demonstrated extensively (4,5,20).

The aim of the present study was to use MRI and investigate the long-term effects of preterm birth on the brain structure. The participants were ex-preterm and term adolescents at the mean age of approximately 15 years who were participants of the Stockholm Neonatal Project (15). Based on previous reports we anticipated a higher incidence of positive findings in the ex-preterm group compared to that of controls. However, of equal importance were how this outcome compared to reports from other centres in the world and whether the minimally invasive care in the local neonatal units made a difference in the outcome of its graduates.

Abbreviations
BW, birth weight; GA, gestational age; MRI, magnetic resonance imaging; PVL, periventricular leucomalacia; IVH, intraventricular haemorrhage; SGA, small for gestational age.

 ©2009 The Author(s)/Journal Compilation ©2009 Foundation Acta Pædiatrica/

METHODS

Subjects

A total of 687 infants were born with birth weight (BW) ≤1500 g in Stockholm County during the period between September 1988 and March 1993. The Stockholm Neonatal Project (15) is a prospective population-based study, derived from this greater cohort, in which infants were included if they were born at (n = 233) or transferred to (n = 58) the Karolinska Hospital or the Löwenströmska Hospital. That is the project includes essentially all very low birth weight infants who needed tertiary care and mechanical ventilation during the period. In addition infants born in the entire county of Stockholm were included if they were born with a BW less than or equal to 1000 g (n = 42). At birth 291 infants were included. The rate of small for gestational age (SGA) infants (BW < 2 SD) was 22.3%. The mortality rate was 17.5% (n = 51) in the neonatal period and four died later due to sudden infant death syndrome. In addition 33 families moved away and 21 declined participation.

The distribution of the remaining 182 with respect to GA and BW was not different from the distribution of the original 291 included at birth, and the rate of those born SGA was 21.6%. They were available for clinical, cognitive and psychological follow-up at 5½ years of age (21). At that time, 125 children were selected from a population-based register according to birth date, birth hospital and having gestational age (GA) of 37 weeks or more in order to form a control group. This control group underwent the same test battery.

Of the 182 cases, 36 did not respond to the initial letter of invitation. Further 54 responded that they did not want to participate or initially were positive but declined on the day of the MRI examination. Sixteen subjects that otherwise would have participated were excluded because of surgical closure of ductus arteriousus with a clip of unknown composition. The remaining 76 of the cases had an MRI examination. One of these subjects could only tolerate the T2-weighted image, while the neonatal records were lost for another.

Of the 125 controls, 22 did not respond to the initial letter of invitation while a further 34 responded negatively or declined on the day of the examination. The total available group and the included subgroup were similar in both groups with respect to GA, BW and gender distribution as well as the mother's age and education level at birth (Table 1).

Based on neonatal ultrasound examinations, the rate of brain injury was slightly lower in the group of ex-preterm participants compared to the rest of the available group choosing not to attend the MRI in adolescence (Table S1). However, the participants were not statistically significantly different from the non-participants on any of the three categories.

Finally, we had useful MRI data from 74 ex-preterm and 69 control adolescents. The two groups did not differ significantly with respect to age, weight or height at the time of MRI scan, or mother's age at birth or mother's level of education (Table S2).

The study was performed with the approval of the local ethics committee. Furthermore, both the participant and an accompanying adult signed a form of written consent.

Collection of MRI data

The MRI examinations were performed between April 2005 and February 2006 at the MR-centre at the Karolinska University Hospital. The order of invitation was randomized including cases and controls in a mixed sequence.

The mean age at the time of MRI examination was 14.92 years (range 12.38–17.17 years).

All images were collected on a 1.5T Signa Excite MR scanner (General Electric, Waukesha, WI, USA). The cranial examination consisted of T1-, T2- and diffusion- weighted images but the neuroradiologists only examined the T1- and T2-weighted images. The diffusion-weighted images will be treated elsewhere separately. All images were collected in an axial orientation (See Table 2 for acquisition details).

Diagnostic procedures of the MRI data

Two experienced neuroradiologists scored all the 143 sets of MRI images. Although the acquisition was axial for all images the radiologists could digitally re-slice the images at any orientation. For example a mid-sagittal view of the corpus callosum was available with 1.5 × 98 mm^2 resolution. Initially, they scored the images independently and blinded as to whether the participant belonged to the group of ex-preterm or control adolescents. When the diagnoses differed the images were re-evaluated and discussed until a consensus was reached. The anticipated findings were gliosis, white matter loss, periventricular haemorrhage and

Table 1 Comparison of the total available group and the included subgroup

	Case group		Control group	
	Total available (n = 182)	Subgroup included (n = 74)	Total available (n = 125)	Subgroup included (n = 69)
GA (weeks)	28.14 (23–36)	28.54 (24–36)	39.75 (37–42)	39.72 (37–42)
BW (g)	1043 (550–1500)	1069 (645–1486)	3494 (2560–4655)	3530 (2750–4655)
Percent of girls	52 (95/182)	51 (38/74)	48 (60/125)	49 (34/69)
Mother's age (years)	30.76 (16–42)	30.66 (20–42)	30.36 (19–44)	30.86 (22–44)
Mother's education (score)	4 (0–6)	4 (2–6)	4 (1–6)	4 (2–6)

The demographic data available indicate that the participants were good representatives of the group from which they were chosen. All data were displayed as mean (range) except percent of girls and mothers' level of education, which were given as percent (ratio) and median (range) respectively.

Table 2 Description of MRI acquisition parameters

	Mode	Sequence	TE (ms)	TR (ms)	Flip angle (degrees)	Voxel size (mm^3)	Echo train length
T1 images	3D	Gradient echo	6	24	30	0.98 × 0.98 × 1.5	N/A
T2 images	2D	Turbo spin echo	84	6000	90	0.98 × 0.98 × 4.0	12

abnormalities with myelination, the corpus callosum or the cerebellum. Each was scored separately with 0 indicating a normal finding and 1–3 representing mild, moderate and severe deviations, respectively. Ventricular dilatation was also noted but counted separately only when white matter loss was not coincident. All incidental abnormalities found were also noted.

Statistical methods

To compare the groups with respect to age, height, weight (at the time of examination) and mother's age at birth two-tailed *t*-tests were employed assuming unequal variances. A two-sample test for binomial proportions was used to compare the gender distribution of the two groups. A two-sample Kolmogorov–Smirnov goodness-of-fit test was used to ascertain that the available 182 individuals did not differ from the originally included 291 with regards to GA and BW and to compare the participants and non-participants with respect to neonatal signs of brain injury (Table S1). The level of education of the mothers had been categorically compiled with 0 = no schooling, 1 = 6 years of schooling, 2 = 9 years of schooling, 3 = less than 3 years of high school, 4 = 3 or more years of high school, 5 = less than 3 years university education, 6 = 3 or more years university education and 7 = doctoral degree. For these data the Wilcoxon rank sum test (a non-parametric analogue of a two-sample *t*-test) was used.

In all cases a p-value less than 0.05 was considered statistically significant.

RESULTS

Suspected abnormalities (Score 0–3)

Of the abnormalities that were given scores from 0 to 3 (i.e. normal, mild, moderate and severe, respectively), myelination was classified as normal in all subjects. No evident corpus callosum abnormalities could be discerned.

Only 17 of the 143 participants had positive findings. Of these two belonged to the group of control subjects (both female) and 15 to the group of ex-preterm subjects (6 female). Only one of the positive findings (a male ex-preterm adolescent) was classified as moderate, all the rest received mild scores. There was no indication that these adolescents came from families of lower social classes. Among the ex-preterm adolescents the median of the mother's level of education was 5 (up to 3 years of university education) compared to a score of 4 (at least 3 years of high-school) as the median of the total group (Table S2).

The mean BW of the cases with positive findings was 993 g (range: 700–1463 g). This was similar to that of the total case group (mean: 1069, range: 645–1486 g) indicating that a lower than average BW in preterm children does not increase the incidence of radiological findings in adolescence. For completeness the BW of the two controls with abnormal radiological findings were 3680 and 4165 g, both above the average of their group (3530 g).

*Cases***:** Altogether we identified 16 cases that were small for gestation age (22). Only 4 of the cases were both SGA and had positive findings (GA range: 30–34 week; BW: 811–1463 g) providing little evidence that being SGA increased the incidence of structural brain injury.

Of the ex-preterm adolescents who had a positive MRI four had gliosis in the middle or posterior region of the supratentorial white matter (Fig. 1). Three of the four subjects with gliosis had no other abnormalities while one subject also had white matter loss in the posterior regions.

Eleven subjects had loss of white matter (Figs. 2A–C) mostly in the middle and posterior periventricular regions. Seven of these had no other abnormalities, while one also had gliosis, two had signs of periventricular haemorrhage

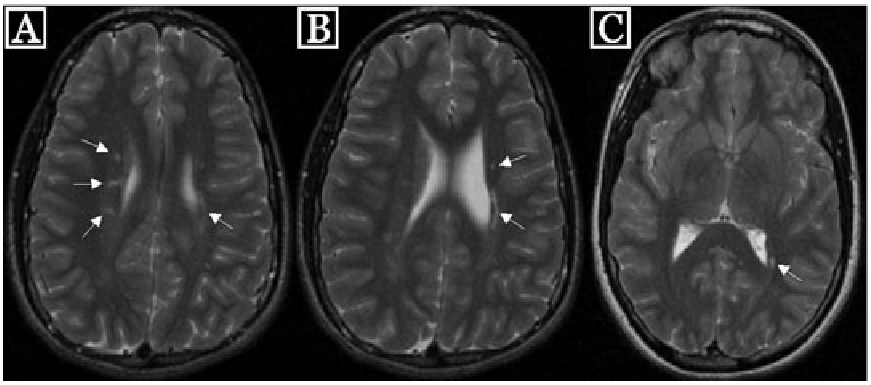

Figure 1 Examples of gliosis from two subjects. A and B belong to the same subject and C illustrates a second subject. The arrows indicate the sites of injury.

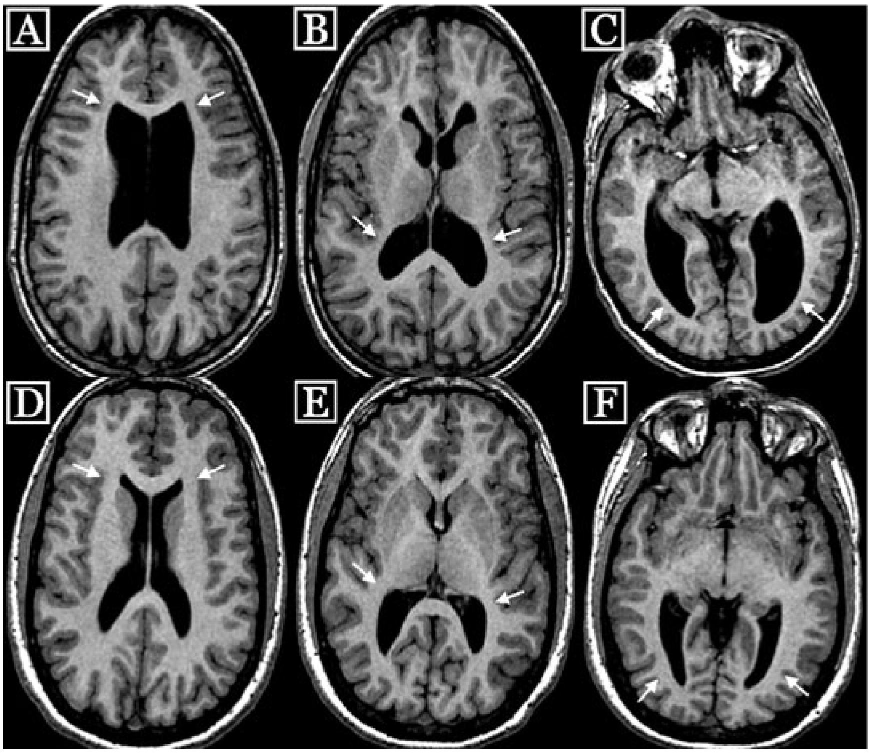

Figure 2 Representative examples to define white matter loss. Three subjects are shown. The top row displays two subjects with positive findings (parts A and B belong to the same subject). The arrowheads indicate the sites of injury in the anterior (A), middle (B) and posterior (C) regions. The bottom row (D–F) displays a single subject with no indication of white matter loss despite the extended ventricles. The slices depict approximately the same axial level as in parts (A–C) and the arrowheads indicate approximately corresponding areas.

and one had a focal cerebellar defect, probably of ischemic origin.

Controls: The two controls both had mild gliosis on the right middle periventricular region.

Additional abnormalities (not scored):

In addition, to the suspected abnormalities that were scored, several other abnormalities were noted.

Cases: Of the ex-preterm adolescents who had a mild or moderate score above, remnant signs of periventricular leucomalacia (PVL), reduced quantity of periventricular white matter and possibly even cavitated infarcts (23), were noted in five. The subject with the cerebellar lesion was also suspected of having had a hypoxic-ischeamic encephalopathy event after the 35th gestational week and had a left occipital/medial temporal infarction with moderate substance loss. One subject was also a suspected case of hydrocephalus.

Among the cases that received a normal score above, two had notable positive MRI findings. One had a suspected cavernoma, a 7-mm low signal lesion in left insula, underneath cortex. Another had a suspected craniopharyngeoma, a <1-cm lesion with high and low signal at posterior suprasellar cistern and tuber cinereum.

Finally, one of the ex-preterm participants had a missing septum pellucidum while in another the 4th ventricle was found to be slightly wider than normal but supposed to be of no significance.

Controls: In the two control subjects who had a mild abnormal score above remnant signs of PVL were noted.

Of the controls without suspected abnormalities above, signs of hypoxic-ischemic encephalopathy were found in one subject with bilateral widening of occipital sulci and possible, partial thinning of cortex in both occipital lobes. No parenchymal lesions were seen. The estimated time of injury was after week 35 of gestation. Another had incidentally high signal on the T2-weighted image in the right petrous apex, which was suspected to be cholesterin granuloma.

Ventricular dilatation: Note that the ventricle size was not explicitly scored. Usually, enlargement of the ventricles coincided with a reduction in white matter. However, if the reduction in white matter was negligible then the images were scored normal even if the ventricles tended to have a larger size (Figs. 2D–F). For completeness 15 ex-preterm individuals and 4 control subjects had enlarged ventricles without any other abnormalities.

DISCUSSION

We have investigated a large cohort of 74 ex-preterm adolescents and 69 controls and found only 21 subjects (4 controls) with notable abnormalities. Within the group of adolescents born preterm, the positive findings did not seem to correlate with GA, BW or being SGA. As expected the rate of positive findings was higher in the ex-preterm group but 17 out

of 74 individuals is below our predicted rate. Of these, two individuals had incidental unexpected abnormalities.

One of the strengths of this study is the number of individuals included. With respect to BW, GA, gender distribution, maternal education and maternal age at birth the individuals who participated did not differ on the average from those that chose not to attend. The second advantage is that it is a prospective study where participants were included at birth, then followed up at $5^1/_2$ years of age.

On the other hand, limitations were also identified. First, due to the fact that the inclusion criteria included BW being less than 1500 g, rather than GA, subjects with longer gestation tended to be small for their GA. The rate in the original cohort was 22.3%. Although the subgroup of participants had a similar rate of 21.6% it is slightly lower than the expected 30%. This can be a weakness in the design (24) because there are indications that children who are born SGA are worse off compared to others born preterm (25) and therefore may lead to an underestimation of the effect of being born SGA in the current study. However, bearing the above in mind, we did not find an obvious connection between being SGA at birth and the extent, severity and frequency of injury. Furthermore, even if GA were used as an inclusion criterion some children who are SGA will still be included and hence the same issue would still persists. Second, a large proportion of the available individuals chose not to participate, more so among the cases than the controls. However those included where good representatives of total available group (Table 1).

There are contradicting reports on whether being born SGA disadvantages a child further (25,26). We found that having been born SGA did not exaggerate the frequency, extent or severity of structural brain injury, although this study was not designed to investigate this dependence and therefore this finding needs further validation.

Neonatal care practices can have a significant effect on the outcome of the individual (12–14) and considering the differences that exists between sites (11) one of the motivations for this study was to investigate whether the local neonatal care practices reflect on outcome of the members of this cohort. For example, over 50% of very low birth weight infants in Stockholm county needed only continuous positive airway pressure as respiratory support (27). The overall incidence of bronchopulmonary dysplasia, retionopathy of prematurity, IVH and PVL in this cohort were significantly lower (28,29). Other factors that may influence outcome in this population are more subtle. The use of mother's own milk and parental attendance in the units that were already encouraged at the time (15) may also play a role. In light of these considerations the relatively scarce findings in this study are perhaps not surprising.

In the ex-preterm group only 17 out of the 74 individuals had any positive findings. All of these were considered mild except for one moderate case. For completeness we also reported benign findings. Even if these are included in the count, the rate of positive findings is still lower than expected. For example, on long-term follow-up, Stewart et al. (5) reported that that only 17 of 72 preterm born individuals had normal MRI findings while Skranes et al. (26) reported MRI pathology in 84% of a large cohort of adolescents born preterm. Correspondingly, Maalouf et al. (30) found only 13 of 41 participants normal upon MRI examination in the newborn period (see also Ref. 1).

It must be noted however, that the low rate of positive findings in this study may, at least partially, be a result of selection bias because the rate of brain injury detected on neonatal ultrasound was slightly lower in the subgroup of participants than those choosing not to attend (Table S1). Although the participants did not differ significantly from rest of the available group, this may create a slightly biased and artificially favourable finding. However, this discrepancy is still inefficient to explain the differences in the rates of positive findings compared to other studies mentioned above. Also, enlargement of the ventricles was only noted as abnormal if it was coincident with white matter reduction. Including all individuals with slight ventricular dilation would increase the rate of positive findings to 30 out of 74, which is still below the rates reported elsewhere (26).

The rate of lesions due to PVL or periventricular haemorrhage was lower than expected in the group of adolescents born preterm. Lower than average weight for GA in these cases did not increase the risk of such lesions. As in other studies, a small number of individuals in the control group also had positive MRI findings (5), and non-symptomatic periventricular lesions (26).

Future directions of investigation will include the measurement of cortical thickness and cortical volume as well as analysis of the diffusion tensor imaging data to scrutinize the integrity of the white matter. In addition, further cognitive and psychological testing of the participants in this cohort is under way.

CONCLUSION

In conjunction with previous results, the rate of positive MRI findings in adolescents was higher in ex-preterm individuals than those born at term. Notably however, the overall rate of MRI abnormalities in the case group was lower than those reported from other centres which may be due to the minimally invasive neonatal care practices at the Karolinska neonatal intensive care unit. Furthermore, being born SGA did not exaggerate these findings further.

ACKNOWLEDGEMENTS

The two neuroradiologists, Drs. Olof Flodmark and Anders Lilja, generously provided their expertise. Many thanks also to Yords Österman, Marie Lundberg, Jonna Karlen and Anna-Karin Edstedt Bonamy. Sällskapet Barnavård, The Swedish Research Council and The Bank of Sweden Tercentenary Foundation funded the study.

References

1. Dyet LE, Kennea N, Counsell SJ, Maalouf EF, Ajayi-Obe M, Duggan PJ, et al. Natural history of brain lesions in extremely preterm infants studied with serial magnetic resonance imaging from birth and neurodevelopmental assessment. *Pediatrics* 2006; 118: 536–48.

2. Rutherford MA. *MRI of the neonatal brain*. London: W.B. Saunders, 2002: 85–95.
3. Huppi PS, Maier SE, Peled S, Zientara GP, Barnes PD, Jolesz FA, et al. Microstructural development of human newborn cerebral white matter assessed in vivo by diffusion tensor magnetic resonance imaging. *Pediatr Res* 1998; 44: 584–90.
4. Peterson BS, Vohr B, Staib LH, Cannistraci CJ, Dolberg A, Schneider KC, et al. Regional brain volume abnormalities and long-term cognitive outcome in preterm infants. *JAMA* 2000; 284: 1939–47.
5. Stewart AL, Rifkin L, Amess PN, Kirkbride V, Townsend JP, Miller DH, et al. Brain structure and neurocognitive and behavioural function in adolescents who were born very preterm. *Lancet* 1999; 353: 1653–7.
6. Malamateniou C, Counsell SJ, Allsop JM, Fitzpatrick JA, Srinivasan L, Cowan FM, et al. The effect of preterm birth on neonatal cerebral vasculature studied with magnetic resonance angiography at 3 Tesla. *Neuroimage* 2006; 32: 1050–9.
7. Wood NS, Marlow N, Costeloe K, Gibson AT, Wilkinson AR. For EPICure Study Group. Neurologic and developmental disability after extremely preterm birth. *N Engl J Med* 2000; 343: 378–84.
8. Isaacs EB, Edmonds CJ, Chong WK, Lucas A, Morley R, Gadian DG. Brain morphometry and IQ measurements in preterm children. *Brain* 2004; 127: 2595–607.
9. Marlow N, Wolke D, Bracewell MA, Samara M. Neurologic and developmental disability at six years of age after extremely preterm birth. *N Engl J Med* 2005; 352: 9–19.
10. Hack M, Flannery DJ, Schluchter M, Cartar L, Borawski E, Klein N. Outcomes in young adulthood for very-low-birth-weight infants. *N Engl J Med* 2002; 346: 149–57.
11. Van Reempts P, Gortner L, Milligan D, Cuttini M, Petrou S, Agostino R, et al. Characteristics of neonatal units that care for very preterm infants in Europe: results from the MOSAIC study. *Pediatrics* 2007; 120: e815–25.
12. Als H, Duffy FH, McAnulty GB, Rivkin MJ, Vajapeyam S, Mulkern RV, et al. Early experience alters brain function and structure. *Pediatrics* 2004; 113: 846–57.
13. Gressens P, Rogido M, Paindaveine B, Sola A. The impact of neonatal intensive care practices on the developing brain. *J Pediatr* 2002; 140: 646–53.
14. Murphy BP, Inder TE, Huppi PS, Warfield S, Zientara GP, Kikinis R, et al. Impaired cerebral cortical gray matter growth after treatment with dexamethasone for neonatal chronic lung disease. *Pediatrics* 2001; 107: 217–21.
15. Katz-Salamon M, Forssberg H, Lagercrantz H. The Stockholm Neonatal Project: very low birthweight infants in the late 20th century in Stockholm. *Acta Paediatr* 1997; 86 Suppl 419: 1–43.
16. Felderhoff-Mueser U, Rutherford MA, Squier WV, Cox P, Maalouf EF, Counsell SJ, et al. Relationship between MR imaging and histopathologic findings of the brain in extremely sick preterm infants. *AJNR Am J Neuroradiol* 1999; 20: 1349–57.
17. Barkovich AJ. Concepts of myelin and myelination in neuroradiology. *AJNR Am J Neuroradiol* 2000; 21: 1099–109.
18. Kostovic I, Judas M, Rados M, Hrabac P. Laminar organization of the human fetal cerebrum revealed by histochemical markers and magnetic resonance imaging. *Cereb Cortex* 2002; 12: 536–44.
19. Battin MR, Maalouf EF, Counsell SJ, Herlihy AH, Rutherford MA, Azzopardi D, et al. Magnetic resonance imaging of the brain in very preterm infants: visualization of the germinal matrix, early myelination, and cortical folding. *Pediatrics* 1998; 101: 957–62.
20. Rutherford MA, Pennock JM, Counsell SJ, Mercuri E, Cowan FM, Dubowitz LM, et al. Abnormal magnetic resonance signal in the internal capsule predicts poor neurodevelopmental outcome in infants with hypoxic-ischemic encephalopathy. *Pediatrics* 1998; 102: 323–8.
21. Bohm B, Katz-Salamon M, Institute K, Smedler AC, Lagercrantz H, Forssberg H. Developmental risks and protective factors for influencing cognitive outcome at 5 1/2 years of age in very-low-birthweight children. *Dev Med Child Neurol* 2002; 44: 508–16.
22. Fenton TR. A new growth chart for preterm babies: Babson and Benda's chart updated with recent data and a new format. *BMC Pediatr* 2003; 3: 13.
23. Flodmark O, Lupton B, Li D, Stimac GK, Roland EH, Hill A, et al. MR imaging of periventricular leukomalacia in childhood. *AJR Am J Roentgenol* 1989; 152: 583–90.
24. Arnold CC, Kramer MS, Hobbs CA, McLean FH, Usher RH. Very low birth weight: a problematic cohort for epidemiologic studies of very small or immature neonates. *Am J Epidemiol* 1991; 134: 604–13.
25. Tolsa CB, Zimine S, Warfield SK, Freschi M, Sancho RA, Lazeyras F, et al. Early alteration of structural and functional brain development in premature infants born with intrauterine growth restriction. *Pediatr Res* 2004; 56: 132–8.
26. Skranes JS, Martinussen M, Smevik O, Myhr G, Indredavik M, Vik T, et al. Cerebral MRI findings in very-low-birth-weight and small-for-gestational-age children at 15 years of age. *Pediatr Radiol* 2005; 35: 758–65.
27. Jonsson B, Katz-Salamon M, Faxelius G, Broberger U, Lagercrantz H. Neonatal care of very-low-birthweight infants in special-care units and neonatal intensive-care units in Stockholm. Early nasal continuous positive airway pressure versus mechanical ventilation: gains and losses. *Acta Paediatr Suppl* 1997; 419: 4–10.
28. Hack M, Horbar JD, Malloy MH, Tyson JE, Wright E, Wright L. Very low birth weight outcomes of the National Institute of Child Health and Human Development Neonatal Network. *Pediatrics* 1991; 87: 587–97.
29. Hesser U, Katz-Salamon M, Mortensson W, Flodmark O, Forssberg H. Diagnosis of intracranial lesions in very-low-birthweight infants by ultrasound: incidence and association with potential risk factors. *Acta Paediatr Suppl* 1997; 419: 16–26.
30. Maalouf EF, Duggan PJ, Rutherford MA, Counsell SJ, Fletcher AM, Battin M, et al. Magnetic resonance imaging of the brain in a cohort of extremely preterm infants. *J Pediatr* 1999; 135: 351–7.

SUPPORTING INFORMATION

Additional Supporting Information may be found in the online version of this article:

Table S1 Differences in signs of brain injury or retinopathy between those included or excluded.
Table S2 Description of the two groups compared in this study.

Please note: Wiley-Blackwell are not responsible for the content or functionality of any supporting materials supplied by the authors. Any queries (other than missing material) should be directed to the corresponding author for the article.
