# Supplementary material for: Correlation between white matter microstructure and executive functions suggests early developmental influence on long fibre tracts in preterm born adolescents
Source: PLoS One. 2017 Jun 8;12(6):e0178893. doi: 10.1371/journal.pone.0178893 (PMC5464584; doi:10.1371/journal.pone.0178893)
Supplement: S1 File — (ZIP) [file pone.0178893.s002.zip › New folder/Stalnacke et al_2014.pdf]

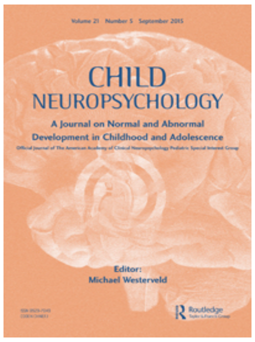

Routledge
Taylor & Francis Group

Child Neuropsychology

A Journal on Normal and Abnormal Development in Childhood and Adolescence

ISSN: 0929-7049 (Print) 1744-4136 (Online) Journal homepage: http://www.tandfonline.com/loi/ncny20

Individual cognitive patterns and developmental trajectories after preterm birth

Johanna Stålnacke, Aiko Lundequist, Birgitta Böhm, Hans Forssberg & Ann-Charlotte Smedler

To cite this article: Johanna Stålnacke, Aiko Lundequist, Birgitta Böhm, Hans Forssberg & Ann-Charlotte Smedler (2015) Individual cognitive patterns and developmental trajectories after preterm birth, Child Neuropsychology, 21:5, 648-667, DOI: 10.1080/09297049.2014.958071

To link to this article: http://dx.doi.org/10.1080/09297049.2014.958071

Published online: 29 Sep 2014.

Submit your article to this journal

Article views: 290

View related articles

View Crossmark data

Citing articles: 2 View citing articles

Full Terms & Conditions of access and use can be found at
http://www.tandfonline.com/action/journalInformation?journalCode=ncny20

Download by: [Karolinska Institutet, University Library] **Date:** 05 November 2016, At: 14:43

Child Neuropsychology, 2015
Vol. 21, No. 5, 648–667, http://dx.doi.org/10.1080/09297049.2014.958071

Routledge
Taylor & Francis Group

Individual cognitive patterns and developmental trajectories after preterm birth

Johanna Stålnacke[1], Aiko Lundequist[1,2], Birgitta Böhm[2], Hans Forssberg[2], and Ann-Charlotte Smedler[1]

[1]Department of Psychology, Stockholm University, Stockholm, Sweden
[2]Department of Women's and Children's Health, Karolinska Institute, Stockholm, Sweden

Cognitive outcome after preterm birth is heterogeneous, and group level analyses may disguise individual variability in development. Using a person-oriented approach, this study investigated individual cognitive patterns and developmental trajectories from preschool age to late adolescence. As part of a prospective longitudinal study, 118 adolescents born preterm, with a birth weight < 1,500 g, participated in neuropsychological assessments at age 5½ years and at 18 years. At each age, four cognitive indices, two tapping general ability and two tapping executive functions, were formed to reflect each individual's cognitive profile. Cluster analyses were performed at each age separately, and individual movements between clusters across time were investigated. At both 5½ and 18 years, six distinct, and similar, cognitive patterns were identified. Executive functions were a weakness for some but not all subgroups, and verbal ability was a strength primarily among those whose overall performance fell within the normal range. Overall, cognitive ability at 5½ years was highly predictive of ability at age 18. Those who performed at low levels at 5½ years did not catch up but rather deteriorated in relative performance. Over half of the individuals who performed above the norm at 5½ years improved their relative performance by age 18. Among those performing around the norm at 5½ years, half improved their relative performance over time, whereas the other half faced increased problems, indicating a need for further developmental monitoring. Perinatal factors were not conclusively related to outcome, stressing the need for cognitive follow-up assessment of the preterm-born child before school entry.

***Keywords*:** Cluster analysis; Executive function; Longitudinal; Parental education; Perinatal factors.

Being born preterm or with low birth weight is associated with risk for neurodevelopmental deficits. Through premature birth, the immature brain is exposed to an environment that affects brain development and places the preterm infant at risk for suboptimal neurocognitive outcome. Processes such as cortical alignment and layering, synaptogenesis, and the

The authors want to acknowledge the contribution of Hugo Lagercrantz to the Stockholm Neonatal Project. This study was supported by grants from the Swedish Council for Working Life and Social Research (Grant 2006-0936), the Faculty of Social Sciences at Stockholm University, Sällskapet Barnavård, and the Foundation Olle Engkvist Byggmästare.

The Stockholm Neonatal Project is a prospective longitudinal cohort study; results from this project are continually published.

Address correspondence to Johanna Stålnacke, Department of Psychology, Stockholm University, SE-106 91 Stockholm, Sweden. E-mail: johanna.stalnacke@psychology.su.se

© 2014 Taylor & Francis

migration and proliferation of the precursor cells that are instrumental for myelination may be affected (Aylward, 2005; Perlman, 2001; Volpe, Kinney, Jensen, & Rosenberg, 2011). Research shows that, although variability in outcome is large, 50% to 70% of children born very or extremely preterm present at least subtle dysfunctions that affect behavioral, intellectual, and educational outcome. These dysfunctions become more obvious with increasing age, as expected development does not occur (Aylward, 2010; Howard, Anderson, & Taylor, 2008).

With regard to general cognitive ability, as measured by IQ, the majority of school-aged children born very or extremely preterm perform within the normal range (Aylward, 2005; Bhutta, Cleves, Casey, Cradock, & Anand, 2002; Böhm, Katz-Salamon, Smedler, Lagercrantz, & Forssberg, 2002; Johnson, 2007). However, their mean scores are lower than is the case for their term-born peers and the distribution is skewed, with a larger than expected number of children having IQ scores in the range bordering to intellectual disability, that is, scores in the 70–85 span (Aylward, 2005). Nonverbal reasoning and visuospatial abilities are often attenuated and a discrepancy between verbal IQ and nonverbal (performance) IQ is common (Aylward, 2005; Johnson, 2007). The relatively few studies of adults born preterm indicate that the described pattern seems to persist (Allin et al., 2008; Hack et al., 2002; Lundequist, 2012).

Of particular interest is the effect of preterm birth on executive function development. Executive function is an umbrella term for a set of cognitive processes important for active and purposeful regulation of thought, emotion, and behavior. These processes are especially important in novel situations or when automatic responses are maladaptive (Anderson, Jacobs, & Anderson, 2008; Diamond, 2013). Inhibition, working memory, and cognitive flexibility are generally regarded as core executive functions (e.g., Diamond, 2013; Miyake et al., 2000). Even a subtle depreciation in executive functions may influence cognitive, social, and academic functioning. Executive function deficits are related to lower IQ scores, will have generalized effects on knowledge acquisition and will also affect social interaction and adaptive functioning. Preterm children are at risk for specific attention and executive function deficits, presumably as an effect of diffuse white matter abnormalities and subsequent disturbances to, perhaps particularly, the frontal-striatal pathways (Howard et al., 2008). Deficits in working memory and higher order executive functions, such as planning, are more clearly associated with perinatal medical complications, particularly problems with oxygenation and confirmed brain injury (Anderson & Doyle, 2004; Taylor, Minich, Bangert, Filipek, & Hack, 2004; Woodward, Edgin, Thompson, & Inder, 2005). However, deficits in sustained attention, requiring inhibitory control, seem to be present even in preterm children without known brain injuries and are likely attributable to the preterm birth per se (Howard et al., 2008).

As pointed out by Gäddlin (2011), relatively few studies have investigated cognitive development of individuals born preterm beyond the early school years and through adolescence. The few long-term studies that do exist, including our own follow-up study of the Stockholm Neonatal Project cohort at age 18, indicate that executive function deficits are likely to persist (Hack et al., 2002; Lundequist, 2012; Luu, Ment, Allan, Schneider, & Vohr, 2011; Taylor, Minich, et al., 2004; Wilson-Ching et al., 2013). The course of development is far from clear: Some studies have reported that cognitive outcome such as working memory deficits observed in childhood may improve with age (Saavalainen et al., 2007; Taylor, Minich, et al., 2004), whereas others have stated that early assessments are only weakly related to later cognitive outcome (Hack et al., 2005).

A wide spectrum of biological and medical factors have been linked to these cognitive deficits; among the more evident are lower gestational age at birth (Bhutta et al., 2002), intrauterine growth restriction (Raz, Debastos, Newman, & Batton, 2012), serious perinatal medical complications (Roze et al., 2009; Vohr et al., 2000), and male sex (Ingemarsson, 2003). By contrast, environmental factors such as higher socioeconomic conditions and parental education are related to better outcome (Taylor, Clayton, & Rowley, 2004). Also, although more perilous outcome is associated with lower gestational age and perinatal medical complications, the individual variability is large, with children of similar medical history exhibiting very different outcomes (Anderson, Howard, & Doyle, 2010; Lundequist, Böhm, & Smedler, 2013).

Reports on outcome after preterm birth are often based on mean endpoints of cross-sectional data. However, given the heterogeneity of outcome among the preterm born, it is important to identify subsets of individuals who present different cognitive patterns and developmental trajectories. In a variable-based analysis, each variable reflects what is characteristic of the average person. Relationships among factors (e.g., cognitive strengths and weaknesses) and their role in the make-up of an individual are assumed to be the same for all individuals. In the person-oriented approach, the meaning of the involved factors is determined by the intra-individual variability and interactions among these factors. The more common variable-oriented approach thus needs to be complemented with a person-oriented approach to uncover the heterogeneity of behavior and development (Bergman, Magnusson, & El-Khouri, 2003; Magnusson & Torestad, 1993).

A few longitudinal studies have investigated patterns of cognitive development among the preterm born. Koller, Lawson, Rose, Wallace, and McCarton (1997) studied individual trajectories of cognitive development from birth to age 6 years in children born with a birth weight less than 1,500 g. For the majority of the children, cognitive abilities at 12 months did not predict outcome at 6 years; however, level of cognitive function showed stability after age 3 to 4 years. The five distinct patterns of cognitive development that they identified were linked to biomedical factors and maternal education. Luu and colleagues (Luu, Vohr, Allan, Schneider, & Ment, 2011) identified distinct trajectories of cognitive development from age 8 to 16 years in a cohort of very preterm children born with a birth weight less than 1,250 g. Developmental trajectories for verbal ability, nonverbal ability, and receptive vocabulary, respectively, were for some subgroups of the very preterm born similar to those of their term-born peers. These more favorable developmental trajectories were associated with lower rates of neurological or sensory impairment and higher levels of maternal education. There were also indications of catch-up to term-born peers with regard to cognitive ability in some subgroups. Both of these studies have identified clusters based on each individual's score on a specific test across several points in time and have thus grouped the individuals in terms of patterns of development in that measure only.

An alternate method for clustering is to, multivariately, identify distinct cognitive profiles across several abilities at a specific point in time, thereby capturing several aspects of the individual simultaneously. In a previous analysis of outcome in attention, memory, sensory-motor, verbal, and visuospatial functions among 5½-year-olds born preterm, we identified subgroups of children with similar neuropsychological profiles as well as overall cognitive levels (Lundequist et al., 2013). Such differences in outcome are disguised in analyses comparing preterm-born children as a cohesive group to a control group. Whether distinct multivariate cognitive outcome patterns remain stable over time is yet unknown.

Within the Stockholm Neonatal Project, we have prospectively followed a cohort of preterm-born individuals from birth to age 18 years; the cluster analysis mentioned above was based on data from age 5½ years. At that age, we also found that general cognitive ability was attenuated compared to full-term peers and that executive functions were a specific weakness (Böhm et al., 2002; Böhm, Smedler, & Forssberg, 2004). At 18 years, this pattern remained, and, on a group level, performance at 18 years was largely predicted by performance at age 5½ years (Lundequist, 2012). However, individual variation in cognitive patterns and their development over time were not addressed in those analyses. To our knowledge, there are no published studies that have investigated individual profiles in cognitive outcome after preterm birth, as well as the long-term developmental trajectories associated with such profiles.

The aim of this study was to identify individual cognitive patterns at preschool age and at late adolescence as well as development over time, in a cohort of individuals born preterm. Specifically:

- Are there clinically meaningful subgroups within the group of preterm born with respect to cognitive profiles at age 5½ years and age 18 years, respectively?
- Do the subgroups show developmental stability from preschool age to adolescence?
- To what extent can these subgroups be predicted by sex, perinatal factors, and parental education levels?

METHOD

Participants

The participants were recruited from the Stockholm Neonatal Project (SNP), a longitudinal prospective population-based study of children born preterm (gestational age less than 37 weeks) and with a birth weight less than 1,500 g. In the initial recruitment process, all children who met these inclusion criteria and were born between September 1988 and March 1993 at Karolinska Hospital and at Löwenströmska Hospital in Stockholm were invited through their parents to participate from birth onwards. In addition, during the same time period, all children from the entire county of Stockholm who were born with a birth weight less than 1,000 g and who were in need of neonatal intensive care at Karolinska Hospital were invited to participate. Extensive medical data were collected in the perinatal period, and a first evaluation of psychomotor development was performed at 10 months of age (corrected for prematurity). Developmental follow-up assessments were performed on two occasions, at age 5½ years (corrected age) and 18 years. Neuropsychological data from the Neuropsychological Assessement 4-7 years (NEPSY 4–7; Korkman, 1990) and the Wechsler Preschool and Primary Scale of Intelligence-Revised (WPPSI-R; Wechsler, 1999) at 5½ years and the Delis-Kaplan Executive Function System (D-KEFS; Delis, Kaplan, & Kramer, 2001) and the Wechsler Intelligence Scale for Children (WISC-III; Wechsler, 1991) at 18 years, as well as data from the perinatal period were included in the analyses in the present study. Perinatal risk factors were originally defined when the study was initiated in 1988, and those included in the present analyses had proven to influence outcome in early follow-up studies. In addition, the Griffiths' Mental Development Scales, Scales A–E (Lindstam, 1968), below referred to as Griffiths, used as an index of psychomotor development at 10 months corrected age, were included.

The 213 children constituting all survivors of the original SNP cohort were invited for a follow-up at 5½ years. At that time, 19 families had moved away from the area and 13 declined to participate, leaving 181 children born preterm available for assessment. All children who participated in the 5½ years of assessment were again invited for another follow-up at 18 years. Ten families had moved and their address could not be identified, and 12 declined participation. In addition, 24 failed to reply. One preterm male was unable to complete the test protocol due to intellectual disability. Hence, 134 preterm children completed the assessment. The original protocol at age 5½ years did not include the full WPPSI-R and 16 eligible participants had completed their assessment prior to its introduction, leaving 118 participants included in the present study. There were no systematic differences in terms of gestational age, birth weight, other perinatal factors, sex, and early cognitive outcome at 5½ years between those who remained in the study and drop-outs. However, participants lost to follow-up at 18 years had mothers with slightly lower education background. This was true also in the control group and is a common pattern in longitudinal studies. Characteristics of the participants are displayed in Table 1.

At 5½ years, a control group (n = 125) was recruited and assessed. The controls were children born at term on the same day and at the same hospital as the preterm children. To be eligible for inclusion, the child was born ≥ 37 weeks of gestation, had a birth weight ≥ 2,500 g, and was classified as a healthy baby at birth. At age 18 years, 91

Table 1 Participant Characteristics.

	M ± *SD or No. (%)*	Range (min-max)
Gestational age at birth (weeks)	27.8 ± 2.8	24–36
Extremely preterm (≤ 27 wks GA)	64 (54.2%)	
Very preterm (28–31 wks GA)	39 (33.0%)	
Moderately preterm, very low birth weight (32–36 wks GA; < 1,500 g)	15 (12.7%)	
Birth weight (g)	1,011 ± 263	550–1,490
Intrauterine growth[a] (BWSDS)	−1.70 ± 1.68	−8.2–1.0
Sex (% males)	53 (45%)	
Multiple pregnancy, no. of children	11 (9.3%)	
Perinatal medical complications[b]		
0 risk factors	82 (69.5%)	
1 risk factor	26 (22.0%)	
2 risk factors	9 (7.6%)	
3 risk factors	1 (0.8%)	
Mother's age at child's birth (yrs)	32.5 ± 5.4	19–43
Mother's educational attainment[c]	4.2 ± 1.4	2–6
Father's educational attainment[c]	4.1 ± 1.5	1–7
Griffiths' Mental Development Scales at 10 months' (n = 99), sum of A-E scales' stanine scores	24.6 ± 9.1	5–44

Notes. N = 118.

[a]*Intrauterine growth*. Birth Weight Standard Deviation Score (BWSDS; Niklasson & Albertsson-Wikland, 2008).

[b]*Perinatal medical complications* are defined as severe levels of intraventricular hemorrhage (IVH) Grade III-V, periventricular leukomalacia, (PVL) Grade III-IV, chronic lung disease (CLD), retinopathy of prematurity (ROP) 3 +.

[c]*Parental education* attainment level as classified by Statistics Sweden (2000). 0 = no education, 1 = noncomplete compulsory education; 2 = completed compulsory education (9 yrs); 3 = two years secondary education; 4 = graduated from secondary school (12 yrs); 5 = Bachelor degree; 6 = Master degree; 7 = Doctoral degree.

controls participated in the study and completed the assessment. Sex, maternal age, and levels of parental education did not differ between the control group and the preterm-born group. The control group scores are used as reference scores in the present study.

SNP was originally approved by the Ethics Committee of Karolinska Hospital (Böhm et al., 2002). Throughout, participation has been based on informed consent, obtained for each data collection. The collection of follow-up data at 18 years as well as the continuing use of the SNP database were approved by the Regional Ethics Board in Stockholm (2007/46-31/3).

Cognitive Indices

Four cognitive indices at each age were formed based on data from age 5½ years and 18 years. Two indices were aimed to reflect the individual's general ability (IQ): Verbal ability (verbal function index; VFI) reflects verbal reasoning and knowledge; nonverbal ability (performance function index; PFI) reflects nonverbal, visuospatial, and abstract reasoning and is less dependent on learning and experience. For the operationalization of these indices, the raw scores of tests included in the Wechsler verbal and performance scales, respectively, were used. Two other indices were aimed to reflect executive functions. A model proposed by Miyake and colleagues (Miyake & Friedman, 2012; Miyake et al., 2000) posits a common factor for all executive function abilities as well as a specific updating and a specific shifting ability. Based on this model, we formed a working memory index (WMI), reflecting the ability to hold in mind and manipulate information, and a cognitive flexibility index (CFI), reflecting the ability to flexibly adjust to new demands or priorities and the ability to change perspectives.

A brief description of the test variables included in the indices, as well as their means, standard deviations, and range are presented in Table 2. At 5½ years, VFI-5 was formed using the results from the verbal tests in the WPPSI-R (Comprehension, Information, Similarities, and Vocabulary); PFI-5 was formed using the results from the performance tests in the WPPSI-R (Block Design, Geometric Designs, Object Assembly, and Picture Completion); WMI-5 was formed using Digit Span from the NEPSY 4–7 and Knox Cubes (Arthur, 1947); CFI-5 was formed using the results from Animal Pegs and Coding from the WPPSI-R and Verbal Fluency and Color Shape from the NEPSY 4–7.

At age 18, VFI-18 was formed from the WISC-III subtests Vocabulary and Similarities; PFI-18 was formed from the WISC-III subtests Block Design and Picture Completion; WMI-18 was formed from the WISC-III subtest Digit Span Backwards and Block Repetition Backwards from the Wechsler Adult Intelligence Scales-Third Edition Neuropsychological Instrument (WAIS-III NI; Wechsler et al., 2004); CFI-18 was formed from the D-KEFS tests Trail Making-4, Color-Word-3, Verbal Fluency-2 and the WISC-III subtests Coding and Symbol Search.

The choice had been made to administer the WISC-III outside the age range for which it was normed rather than WAIS-III to ascertain adequate differentiation between low-performing participants. The raw scores for both the preterm and control groups were used in the analyses and no ceiling effects were observed.

For each age separately, a Confirmatory Factor Analysis (results not shown) was performed to ensure that the theoretically composed indices fit the data. The models had good fit at both ages, and at both ages, our hypothesized model with four indices had a better fit compared to allowing all tests to load on one common factor. We thus concluded that our operationalizations were valid to use for further analyses.

Table 2 Included Variables, Brief Description, Mean (*z*-Score), Standard Deviation and Range.

Variables and Indices	Variable Description	Variable Characteristics			
		N	Mean	*SD*	Range
VFI-5	Verbal ability index at age 5½, *z*-score	118	−0.43	1.18	−3.6–1.8
Comprehension (WPPSI)	Questions about social situations or common concepts		−0.43	1.55	0–29
Information (WPPSI)	General knowledge questions		−0.59	1.24	10–26
Similarities (WPPSI)	The child is asked to explain how two words (nouns) are alike/similar.		−0.45	1.13	0–24
Vocabulary (WPPSI)	The child is asked to name or define words of increasing difficulty.		−0.27	1.45	7–36
PFI-5	Nonverbal ability index at age 5½, z-score	118	−0.46	0.99	−4.2–1.6
Block Design (WPPSI)	The child is asked to put together red-and-white blocks in a pattern according to a displayed model.		−0.67	1.18	2–34
Geometric Designs (WPPSI)	The child is asked to copy simple geometric designs.		−0.34	1.10	1–57
Object Assembly (WPPSI)	The child is presented with the pieces of a puzzle in a standard arrangement and fits the pieces together to form a meaningful whole within 90 seconds.		−0.51	1.24	9–30
Picture Completion (WPPSI)	The child is shown artwork of common objects with a missing part, and asked to identify the missing part by pointing to and/or naming it.		−0.33	1.16	5–24
WMI-5	Working memory index at age 5½, *z*-score	118	−0.30	0.86	−3.6–1.4
Digit Span (NEPSY)	The child is asked to repeat in exact order an increasing number of digits.		−0.28	1.17	0–5
Knox Cubes	The examiner taps a sequence on four cubes attached horizontally to wooden board and the child has to repeat it in the correct order.		−0.32	0.95	0–10
CFI-5	Cognitive flexibility index at age 5½, *z*-score	118	−0.53	0.88	−3.8–1.1
Animal Pegs (WPPSI)	Requires the child to, as fast as possible, match animals to colored pegs on a board according to a code.		−0.79	1.53	5–60
Coding (WPPSI)	The child is asked to mark rows of shapes with different lines according to a code. Speed test.		−0.35	1.15	0–46
Verbal Fluency (NEPSY)	The child has to list as many animals he or she can think of in a minute, followed by generating a list of things you can eat or drink.		−0.47	1.02	3–33
Color Shape (NEPSY)	The child is requested to point a path across a board by alternating between similar shapes and similar colors.		−0.50	1.10	0–9

Notes. WPPSI refers to WPPSI-R and NEPSY to NEPSY 4–7. For the indices, range is expressed as a *z*-score range; for the tests' scores, range refers to raw scores. *Z*-scores were formed using the mean and standard deviation of the SNP control group and thus reflect level *vis-à-vis* norm.

Table 2 (Continued).
Included Variables.

Variables and Indices	Variable Description	Variable Characteristics			
		N	Mean	*SD*	Range
VFI-18	Verbal ability index at age 18, *z*-score	118	−0.25	1.25	−4.6–1.5
Similarities (WISC)	The child is asked to explain how two words (nouns) are alike/similar.		−0.32	1.30	1–32
Vocabulary (WISC)	The child is asked to name or define words of increasing difficulty.		−1.19	1.36	18–60
PFI-18	Nonverbal ability index at age 18, *z*-score	118	−0.38	1.08	−3-9–1.4
Block Design (WISC)	The child is asked to put together red-and-white blocks in a pattern according to a displayed model.		−0.53	1.26	13–69
Picture Completion (WISC)	Name the pertinent detail that is missing from a picture.		−0.22	1.13	9–28
WMI-18	Working memory index at age 18, *z*-score	118	−0.45	0.96	−3.5–1.8
Corsi Block (WAIS)	A spatial memory task whereby the child is requested to point to blocks on a board in the exact opposite order as the test administrator.		−0.53	1.33	2–12
Digit Span (WISC)	An increasing number of digits are to be repeated in the exact opposite order as given by the test administrator.		−0.38	1.05	2–11
CFI-18	Cognitive flexibility index at age 18, *z*-score	118	−0.64	1.17	−4-7–1.5
Verbal Fluency 2 (D-KEFS)	Semantic categories. List as many words as possible in a minute that fit into the given category (animals and boys names, respectively).		−0.35	1.39	13–63
Trail Making 4 (D-KEFS)	Connect by drawing a line to every other letter and every other digit. Letters in alphabetical order, digits in increasing order.		−1.10	2.21	36–470
Color-Word 3 (D-KEFS)	Stroop test. When reading the name of colors printed in different colors, the task is to name the printed color rather than read the print.		−0.61	1.55	32–195
Coding (WISC)	The task is to transcribe a digit-symbol code and complete as many as possible in 2 minutes.		−0.48	1.05	27–98
Symbol Search (WISC)	Rows of symbols and target symbols, the child is asked to mark whether or not the target symbols appear in each row. Complete as many as possible in 2 minutes.		−0.67	1.37	8–45

Notes. WISC refers to WISC-III and WAIS to WAIS-III NI. For the indices, range is expressed as the *z*-score range; for the tests' score, range refers to raw scores. *Z*-scores were formed using the mean and standard deviation of the SNP control group and thus reflect level *vis-à-vis* norm.

To create the indices used in the pattern analysis, each test score was *z* transformed using the mean and standard deviation from the SNP control group scores. The *z* sum for each index was divided by the number of test scores included, resulting in an average *z*-score for each index. The index score thus reflects performance relative to the term-born control group.

Pattern Analysis

The Linking of Clusters after Removal of a Residue (LICUR) analysis (Bergman et al., 2003), a method for cross-sectional classification analysis followed by linkage over time, was performed to discern patterns and their stability and change over time. In this method, clusters of individuals with similar cognitive patterns are identified first at one point in time (age 5½ years in our case) and then, independently, at another point in time (at 18 years). The patterns themselves are subsequently compared to see if typical cognitive patterns at age 5½ are similar to the cognitive patterns at age 18. If similar patterns exist at both points in time, this would imply structural stability. Of interest is also to see if a specific individual has a similar cognitive pattern at both ages, that is, is found in clusters with similar patterns. The streams of how individuals move across time gives an indication of developmental trajectories. LICUR thus involves four steps: First, identification of multivariate outliers (residue) separately for each age; these are individuals who have a unique pattern of test scores. Second, cluster analyses of the individuals separately at each age. Third, linking of the classifications at each age to one another, and, fourth, analysis of individuals' movements across the two time points. The LICUR analysis was performed in Sleipner (Bergman & El-Khouri, 2002).

Ward's cluster analysis, a hierarchical agglomerative method, was applied. The agglomerative method initially assumes each individual case to be a cluster and, at iteration, the two clusters with the most similar scores on all four indices, that is, VFI, PFI, WMI, and CFI, are fused. Similarity was measured in squared Euclidean distance. The Ward method fuses the two clusters that result in a minimum increase in variance. The cluster solution chosen should contain theoretically meaningful and sufficiently homogenous clusters that together account for at least two thirds of the explained variance. The cluster analyses, based on each individual's four index *z*-scores were performed after excluding multivariate outliers, on the data sets from age 5½ years and age 18 years, respectively.

Structural stability of the clusters between the two points in time was investigated. This analysis compares clustering solutions by matching each cluster centroid in the 5½-year solution to the most similar cluster centroid from the 18-year solution; what clusters are similar and to what extent. The clusters are pairwise matched step by step. The two clusters from different time points that are most similar with regard to centroids are matched first; from the remaining clusters, the most similar clusters are then matched, etcetera.

To study how individuals move from one cluster at age 5½ years to another cluster at age 18 years, exact analysis of single cells in a contingency table was used. In these analyses the typical and atypical longitudinal streams can be examined (Bergman et al., 2003).

Analyses of variable and participant characteristics as well as background characteristics of the identified clusters were performed in SPSS 22 for Windows (SPSS, Inc., Chicago, Illinois). Comparisons concerning cluster characteristics were made using Fisher's exact test or analysis of variance (ANOVA). A *p*-value of less than .05 (two-sided) was considered to indicate statistical significance.

RESULTS

Residue

At 5½ years, three multivariate outliers, or residue cases, were identified and were not included in the cluster analysis. At 18 years, again three residue cases were identified and removed prior to the cluster analysis. None of the residue cases at 5½ were also a residue at 18 years.

Clusters at Age 5½ Years and at Age 18 Years

At age 5½ years, the six-cluster solution best matched the criteria. The explained variance for this solution was 66.8%, which is in line with the recommendation. The homogeneity of five of the clusters was sufficient; however, the cluster with the lowest scores (5F) was less homogeneous than ideal, homogeneity coefficient = 1.1. The solution is presented in Figure 1; the homogeneity coefficients and the means and standard deviations of the cluster centroids are presented in the top part of Table 3. The clusters were labelled from 5A to 5F in order of level of performance with 5A being the highest performing cluster.

Again, at age 18, the six-cluster solution was deemed the most meaningful. The explained variance was 71.5% and thus surpassed the recommended two thirds. The clusters were also sufficiently homogeneous. The clusters were labelled 18A+, 18A, 18B, 18D, 18E, and 18F in level of performance with 18A+ being the highest performing

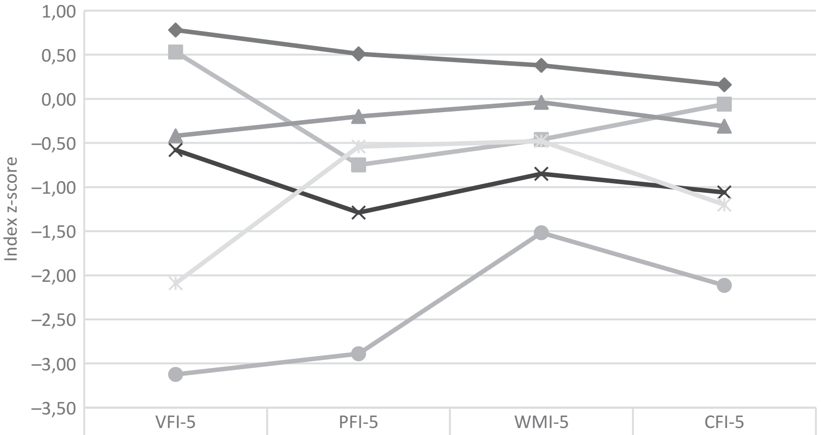

	VFI-5	PFI-5	WMI-5	CFI-5
Cluster 5A (n = 26)	0,78	0,51	0,38	0,16
Cluster 5B (n = 13)	0,53	−0,75	−0,46	−0,06
Cluster 5C (n = 39)	−0,42	−0,20	−0,04	−0,31
Cluster 5D (n = 19)	−0,58	−1,29	−0,85	−1,06
Cluster 5E (n = 13)	−2,09	−0,54	−0,48	−1,20
Cluster 5F (n = 5)	−3,12	−2,89	−1,52	−2,11

Figure 1 Identified clusters at age 5½. Scores represent the index centroid (average score for the individuals who make up the cluster). *Z*-scores relative to term-born peers.

Table 3 Six-Cluster Solution Descriptions (5½ Years).

	Clusters							
	5A	5B	5C	5D	5E	5F	Total	*p*-value
n	26	13	39	19	13	5	115	
VFI-5	0.78 ± 0.42	0.53 ± 0.28	−0.42 ± 0.46	−0.58 ± 0.56	−2.09 ± 0.52	−3.12 ± 0.54		
PFI-5	0.51 ± 0.50	−0.75 ± 0.39	−0.20 ± 0.52	−1.29 ± 0.50	−0.54 ± 0.70	−2.89 ± 1.15		
WMI-5	0.38 ± 0.77	−0.46 ± 0.36	−0.04 ± 0.62	−0.85 ± 0.56	−0.48 ± 0.44	−1.52 ± 0.62		
CFI-5	0.16 ± 0.56	−0.06 ± 0.39	−0.31 ± 0.59	−1.06 ± 0.49	−1.20 ± 0.63	−2.11 ± 0.27		
Homogeneity coefficient	0.67	0.26	0.61	0.56	0.68	1.10		
EESS							66.8%	
Sex (no. of boys)	12 (46.2)	6 (46,2)	15 (38.5)	10 (52.6)	4 (30.8)	4 (80.0)	51 (44.3)	.49
Gestational age (weeks)	28.2 ± 2.1	27.7 ± 3.0	28.2 ± 2.9	27.8 ± 2.7	27.5 ± 3.7	24.6 ± 0.9	27.8 ± 2.8	.17
Birth weight (g)	1,045 ± 256	971 ± 204	1,069 ± 279	981 ± 287	969 ± 275	744 ± 46	1,013 ± 266	.14
Intrauterine growth	−1.8 ± 1.7	−1.8 ± 2.3	−1.7 ± 1.4	−1.9 ± 1.4	−1.6 ± 2.5	−0.6 ± 0.9	−1.7 ± 1.7	.75
Perinatal medical complications								.004
0 risk factors	25^{a} (21.7)	8 (61.5)	29 (74.4)	11^{a} (57.9)	6 (46.2)	2 (40.0)	81 (70.4)	
1 risk factor	1^{b} (3.8)	3 (23.1)	9 (23.1)	$4^{a,b}$ (21.1)	5 (38.5)	2 (40.0)	24 (20.9)	
2 or 3 risk factors	0^{b} (0.0)	2 (15.4)	1 (2.6)	4^{b} (21.1)	2 (15.4)	1 (20.0)	10 (8.7)	
Parental education								.001
≤ 9 years (1 + 2)	0 (0.0)	0 (0.0)	$1^{a,b}$ (2.6)	0 (0.0)	1 (7.7)	3^{a} (60.0)	5 (4.3)	
10–12 years (3 + 4)	4 (15.4)	1 (7.7)	16^{b} (41.0)	8 (42.1)	3 (23.1)	1^{b} (20.0)	33 (28.7)	
University degree (5–7)	22 (84.6)	12 (92.3)	22^{a} (56.4)	11 (57.9)	9 (69.2)	1^{b} (20.0)	77 (67.0)	
Grifffiths' Mental Development Scales at 10 months	30.2 ± 8.4	26.0 ± 9.5	25.7 ± 7.9	21.8 ± 7.5	18.6 ± 8.4	12.5 ± 5.8	24.9 ± 8.9	<.001

Notes. Data are mean ± standard deviation or number (%).

p-value; for continuous variables, ANOVA; for categorical data, Fisher's exact test.

VFI-5, PFI-5, WMI-5, and CFI-5 are represented by the *z*-score relative to term-born peers of the cluster centroids for Verbal Ability Index, Nonverbal Ability Index, Working Memory Index, and Cognitive Flexibility Index, respectively. The centroids are the means of the indices computed across the subjects that form the cluster.

For homogeneity coefficient (HC), a lower number implies a more homogenous cluster, that is, containing individuals with very similar profile scores. Ideally HC < 1 (Bergman et al., 2003).

Explained Error Sum of Squares (EESS) is the percent of variance explained by the chosen cluster solution. Ideally EESS > 67% (Bergman et al., 2003).

Intrauterine growth expressed as Birth Weight Standard Deviation Score (BWSDS; Niklasson & Albertsson-Wikland, 2008).

Perinatal medical complications are defined as severe levels of intraventricular hemorrhage (IVH) Grade III-V, periventricular leukomalacia (PVL) Grade III–IV, chronic lung disease (CLD), retinopathy of prematurity (ROP) 3 +.

Parental education attainment level as classified by Statistics Sweden (2000): 0 = no education; 1 = noncomplete compulsory education; 2 = completed compulsory education (9 yrs); 3 = two years secondary education; 4 = graduated from secondary school (12 yrs); 5 = Bachelor degree; 6 = Master degree; 7 = Doctoral degree. Parent with highest level of education is represented.

[a] [b]Each subscript letter denotes a subset of medical complications or parental education categories whose column proportions do not differ significantly from each other at the .05 level.

Griffiths' Mental Development Scales at 10 months is the sum of the A–E scales' stanine scores. Total $N = 96$; 5A $n = 21$, 5B $n = 11$, 5C $n = 35$, 5D $n = 17$, 5E $n = 8$, 5F $n = 4$. Post hoc analyses: 5F < 5A, 5B, 5C; 5A > 5D, 5E, 5F.

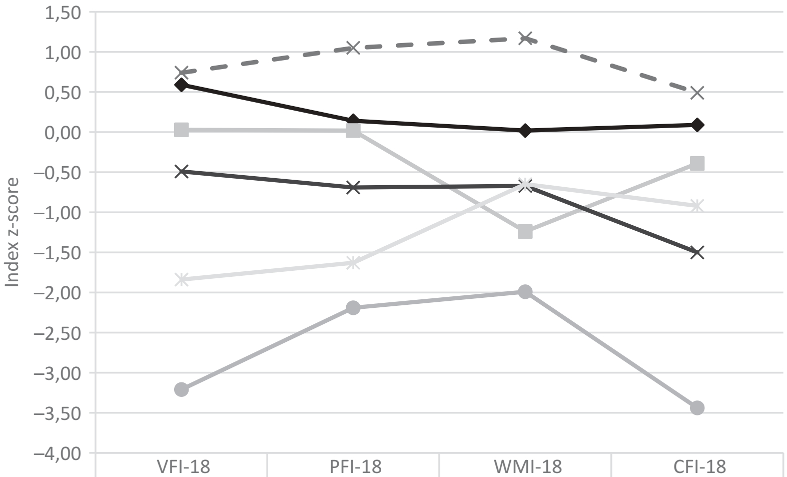

	VFI-18	PFI-18	WMI-18	CFI-18
Cluster 18A+ (n = 10)	0,74	1,05	1,17	0,49
Cluster 18A (n = 42)	0,59	0,14	0,02	0,09
Cluster 18B (n = 16)	0,03	0,02	−1,24	−0,39
Cluster 18D (n = 26)	−0,49	−0,69	−0,67	−1,50
Cluster 18E (n = 16)	−1,84	−1,63	−0,65	−0,92
Cluster 18F (n = 5)	−3,21	−2,19	−1,99	−3,44

Figure 2 Identified clusters at age 18. Scores represent the index centroid (average score for the individuals who make up the cluster). *Z*-scores relative to term-born peers.

cluster. The solution is presented in Figure 2; the homogeneity coefficients and the means and standard deviations of the cluster centroids are presented in the top part of Table 4.

Structural Stability and Longitudinal Streams

The pairwise matching of similar clusters across time revealed that five of the clusters at 5½ years corresponded well or rather well to a cluster at age 18, as measured by average squared Euclidean distance (ASED). The exceptions were Cluster 5C, which had no match at age 18 years, and Cluster 18A+, which was essentially a breakout from Cluster 18A.

How the individuals moved from one cluster at 5½ years to another cluster at 18 years is depicted in Figure 3 and the contingency table is presented in Figure 4. All participants in Cluster 18A+ came from the 5A cluster. The remaining 5A participants were almost all to be found in Cluster 18A. Participants from Cluster 5B typically moved to Cluster 18B. The large Cluster 5C split up, with almost half of the participants improving in performance relative to term-born peers (to Cluster 18A) and the rest maintaining or deteriorating in performance over time. Movements from clusters performing at norm to low clusters, that is, from the 5A or 5B cluster at age 5½ years to Clusters 18D, 18E, or 18F at age 18 were extremely rare. Likewise, movements from the low clusters at 5½ years to the higher clusters at age 18 were unusual. There were no transfers from Clusters 5D, 5E, or 5F to Clusters 18A+ or 18B; only a few transfers occurred to 18A.

Table 4 Six-Cluster Solution Descriptions (18 Years).

	Clusters							
	18A+	18A	18B	18D	18E	18F	Total	*p*-value
n	10	42	16	26	16	5	115	
VFI-18	0.74 ± 0.51	0.59 ± 0.53	0.03 ± 0.57	−0.49 ± 0.66	−1.84 ± 0.47	−3.21 ± 0.47		
PFI-18	1.05 ± 0.27	0.14 ± 0.57	0.02 ± 0.30	−0.69 ± 0.57	−1.63 ± 0.73	−2.19 ± 0.45		
WMI-18	1.17 ± 0.39	0.02 ± 0.53	−1.24 ± 0.53	−0.67 ± 0.57	−0.65 ± 0.54	−1.99 ± 0.28		
CFI-18	0.49 ± 0.53	0.09 ± 0.56	−0.39 ± 0.49	−1.50 ± 0.61	−0.92 ± 0.84	−3.44 ± 0.98		
Homogeneity coefficient	0.38	0.60	0.61	0.73	0.88	0.73		
EESS							71.5%	
Sex (no. of boys)	4 (40.0)	16 (38.1)	8 (50.0)	13 (50.0)	8 (50.0)	1 (20.0)	50 (43.5)	
Gestational age (weeks)	27.9 ± 2.1	28.3 ± 2.9	27.7 ± 1.9	27.4 ± 3.0	27.7 ± 3.0	28.4 ± 4.3	27.9 ± 2.8	.85
Birth weight (g)	1,044 ± 275	1,065 ± 262	1,036 ± 233	928 ± 247	1,013 ± 311	927 ± 218	1,015 ± 262	.40
Intrauterine growth	−1.6 ± 1.5	−1.7 ± 1.8	−1.5 ± 1.2	−1.8 ± 1.3	−1.6 ± 1.7	−2.5 ± 1.5	−1.7 ± 1.7	.90
Perinatal medical complications								<.001
0 risk factors	10 (100.0)	37^{a} (88.1)	7^{a} (43.8)	13^{a} (50.0)	11 (68.8)	2 (40.0)	80 (80.0)	
1 risk factor	0 (0.0)	3^{b} (7.1)	9^{b} (56.3)	$8^{a,b}$ (30.8)	3 (18.8)	2 (40.0)	25 (21.7)	
2 or 3 risk factors	0 (0.0)	$2^{a,b}$ (4.8)	0 (0.0)	5^{b} (19.2)	2 (12.5)	1 (20.0)	10 (8.7)	
Parental education								.13
≤ 9 years (1 + 2)	$0^{a,b}$ (0.0)	1 (2.4)	0 (0.0)	0 (0.0)	1 (6.3)	1^{a} (20.0)	3 (2.6)	
10–12 years (3 + 4)	0^{b} (0.0)	11 (26.2)	5 (31.3)	9 (34.6)	7 (43.8)	1^{b} (20.0)	33 (28.7)	
University degree (5–7)	10^{a} (100.0)	30 (71.4)	11 (68.8)	17 (65.4)	8 (50.0)	3^{b} (60.0)	79 (68.7)	
Grifffiths' Mental Development Scales at 10 months	28.3 ± 9.0	28.8 ± 7.9	23.7 ± 7.5	21.7 ± 8.9	22.1 ± 8.1	12.5 ± 7.4	24.8 ± 9.0	.001

Notes. Data are mean ± standard deviation or number (%).

p-value; for continuous variables, ANOVA; for categorical data, Fisher's exact test.

VFI-18, PFI-18, WMI-18, and CFI-18 are represented by the *z*-score relative to term-born peers of the cluster centroids for Verbal Ability Index, Nonverbal Ability Index, Working Memory Index, and Cognitive Flexibility Index, respectively. The centroids are the means of the indices computed across the subjects that form the cluster.

Homogeneity coefficient (HC) where the lower number implies a more homogenous cluster, that is, containing individuals with very similar profile scores. Ideally HC < 1 (Bergman et al., 2003).

Explained Error Sum of Squares (EESS) where the percent of variance explained by the chosen cluster solution. Ideally EESS > 67% (Bergman et al., 2003).

Intrauterine growth expressed as Birth Weight Standard Deviation Score (BWSDS; Niklasson & Albertsson-Wikland, 2008).

Perinatal medical complications are defined as severe levels of intraventricular hemorrhage (IVH) Grade III–V, periventricular leukomalacia (PVL) Grade III–IV, chronic lung disease (CLD), retinopathy of prematurity (ROP) 3 +.

Parental education attainment level as classified by Statistics Sweden (2000): 0 = no education; 1 = noncomplete compulsory education; 2 = completed compulsory education (9 yrs); 3 = two years secondary education; 4 = graduated from secondary school (12 yrs); 5 = Bachelor degree; 6 = Master degree; 7 = Doctoral degree. Parent with highest level of education is represented.

[a b] Each subscript letter denotes a subset of medical complications or parental education categories whose column proportions do not differ significantly from each other at the .05 level.

Griffiths' Mental Development Scales at 10 months, sum of the A-E scales' stanine scores. Total $N = 97$; 18A+ $n = 7$, 18A $n = 36$, 18B $n = 13$, 18D $n = 23$, 18E $n = 14$, 18 $n = 4$. Post hoc analyses: 18F < 18A+, 18A; 18D < 18A.

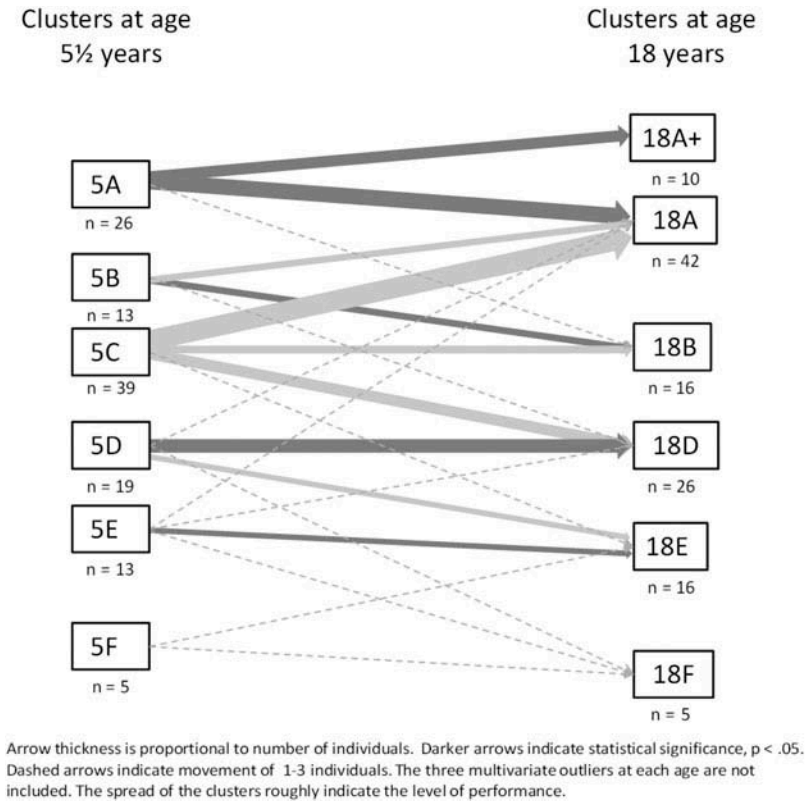

Figure 3 Longitudinal streams.

Age 18 years

Age 5½ years	18A+	18A	18B	18D	18E	18F	Residue	n =
5A	**10*** **	**14***	2	[***]	[*]			26
5B		6	**6** **	1				13
5C	[*]	**18** (*)	7	10	3		1	39
5D		1 [**]	[*]	**12** ***	**5** (*)	1		19
5E		3		3	**5** *	**2** (*)		13
5F					2	1	**2** *	5
Residue			1		1	1		3
n =	10	42	16	26	16	5	3	

*** p-value<0.001
** p-value<0.01
* p-value <0.05
(*) p-value <0.10

[ ] – Significance of rare or dry streams

Figure 4 Significant movements and rare or dry streams between the identified clusters at age 5½ years and age 18 years.

The residue cases from 5½ years, one case with extremely low working memory but above the norm in nonverbal ability at 5½ years and two cases with extremely low scores on all four indices at 5½ years, were members of Cluster 18B and of Clusters 18E and 18F, respectively, at age 18 years. Two of the cases found in Cluster 5F at age 5½ years were residue cases at 18 years. One case from Cluster 5C was a residue at age 18; he scored above the norm on three indices but extremely low on working memory.

Background Characteristics and Cluster Solutions

The clusters were analyzed on membership background characteristics: gestational age at birth, birth weight, intrauterine growth (expressed as birth weight standard deviation score), and perinatal medical complications, as well as sex and level of parental education. The association between cluster membership and the Griffiths at 10 months was also analyzed.

Age 5½ years. Among individuals in Cluster 5A, absence of perinatal medical complications and, among individuals in the 5D cluster, the presence of two or more perinatal medical complications were overrepresented. In Cluster 5F, parents with only primary school (ninth grade) education attainment were overrepresented; in Cluster 5C, parents with secondary (twelfth grade) education were overrepresented. Cluster membership was associated with the 10-month Griffiths scores and post hoc analyses revealed that individuals in Cluster 5A performed significantly higher than those in Clusters 5D, 5E, and 5F; individuals in Cluster 5F performed significantly lower than those in Clusters 5A, 5B, and 5C. There were no other statistically significant differences between the clusters. The lower part of Table 3 displays the background characteristics for each cluster separately as well as associated *p*-values.

Age 18 years. Among individuals in Cluster 18A, absence of perinatal medical complications, in Cluster 18B, the presence of one perinatal medical complication and, in Cluster 18D, the presence of two or more medical complications were overrepresented. Cluster membership was associated with the 10-month Griffiths scores and the post hoc analyses revealed that individuals in Cluster 18A+ had scored significantly higher on the Griffiths than those in Cluster 18F. Likewise, Cluster 18A had scored significantly higher on the Griffiths than Clusters 18D and 18F. None of the 10 members of Cluster 18A+ experienced perinatal medical complications and all had at least one parent with a university degree; these associations, however, were not statistically significant. The lower part of Table 4 displays the background characteristics for each cluster separately.

DISCUSSION

Indeed, we were able to identify meaningful subgroups within the group of preterm born, with respect to cognitive profiles at age 5½ and age 18 years, respectively. There were six subgroups at both ages, based primarily on overall level of performance on the tests that constituted our cognitive indices. However, at both ages individual variation with regard to cognitive strengths and weaknesses were also apparent. In some subgroups, the pattern was even, with individuals performing at the same level relative to term-born controls on all four index measures, whereas individuals in some subgroups were characterized by a wide variability in relative performance. Those with a relative strength or

relative weakness in verbal ability, or a relative strength in working memory at 5½ years, tended to show the same strength or weakness at age 18. Individuals with even cognitive profiles at 5½ years continued to exhibit even profiles at age 18. At 18 years, Clusters 18A+ and 18A performed at or above the norm on all four indices, that is, in essence these individuals were cognitively rather well functioning despite their suboptimal start in life. The individuals in Cluster 18B displayed a pattern of at-norm performance in general ability but with a weakness in executive functions, especially working memory. This profile is in line with studies that have reported executive function to be a specific weakness among those born preterm (Böhm et al., 2004; Howard et al., 2008; Lundequist, 2012). Verbal performance is often cited as a relative strength among those born preterm (Aylward, 2005; Johnson, 2007). In our study, this was not a typical profile. Notably, for those subgroups performing significantly below the norm, verbal ability was rather a relative weakness. Perhaps this can be understood in terms of verbal ability reflecting the ability to learn and accumulate knowledge, which in turn requires nonverbal and executive abilities. Thus, for those whose preterm birth has had a clear negative impact on brain development, the ability to learn is hampered.

Comparing clusters across time addresses the stability in cognitive patterns— that is, were the patterns identified at 5½ years essentially the same as those identified at age 18? In our analyses, the patterns identified at the two ages displayed good structural stability. Indeed, five of the six identified patterns had a good match across the two points in time. However, the stability in patterns does not necessarily imply stability in individual development. An indication of developmental stability is that the same individuals tend to be classified into clusters of similar levels and profiles at the two time points. By studying the longitudinal streams, that is, how individuals moved from one cluster at age 5½ years to a cluster at age 18 years, individual stability in cognitive pattern could also be analyzed. In our sample, most individuals moved to a cluster of similar level and profile. Indeed, movements from the higher clusters (5A, 5B) to a lower cluster (18D, 18E, or 18F) were extremely rare, as were movements from a low to a high cluster. Thus, stability was present both on a structural and an individual level. Of interest was the pattern of Cluster 5C, which was not matched by any cluster at age 18. The cognitive profile of Cluster 5C was even, and performance was slightly below norm. Almost half of the members of this cluster at 5½ years improved their performance and, by age 18, were assigned to a cluster with performance above the norm. Some exhibited a general ability at the norm with an executive weakness at age 18, whereas approximately one third had deteriorated in relative performance.

Previous studies have been inconclusive with regard to whether the developmental deficits observed at early ages will persist or whether catch-up effects are to be expected (Hack et al., 2005; Saavalainen et al., 2007; Taylor, Minich, et al., 2004). In this study, the individuals in the lower performing clusters at age 5½ years were unlikely to move to clusters performing at norm at age 18— that is, there was no sign of catch-up among those who lagged behind at late preschool age. Rather, there are indications of a relative deterioration in performance. On the other hand, the presence of a higher cluster at age 18, the 18A+ cluster, implies a relative improvement in performance compared to full-term peers. A good half of the individuals assigned to Clusters 5A, 5B, or 5C were assigned to a higher performing cluster at age 18, the majority to clusters performing at or above the norm on all four indices. That is, those who did well at age 5½ continued to do so and may even have improved their relative performance.

The relationship between the background characteristics and cluster membership was investigated. The analyses revealed that cluster membership could not be predicted by the background variables, neither at age 5½ years nor at 18 years. This means that perinatal data alone cannot predict outcome in preschool years or adolescence. However, the Griffiths developmental score at 10 months had some predictive value: Those performing very low were likely to score low on cognitive tests at age 5½ and 18 years. But, there were individuals who scored in the tenth percentile or lower on the Griffiths as infants who at 5½ years or 18 years were members of a high-performing cluster. Parental education and perinatal medical complications were associated with cluster membership but not in a conclusive manner. Well-educated parents as well as no experience of perinatal medical complications were protective factors and were associated with higher levels of cognitive performance. Still, these seemingly protective factors were not strong enough to predict cluster membership. Interestingly, the high-performing break-out cluster at age 18 (18A+) was entirely composed of individuals who had not suffered from perinatal medical complications and who had well-educated parents. This is in line with the findings from Luu and colleagues (Luu, Vohr, et al., 2011), who also showed that individuals with developmental trajectories similar to controls had mothers with a higher level of education and had lower rates of neurological or sensory impairments.

Our findings stress the importance of follow-up and continued monitoring before school entry of the preterm-born child, even after a seemingly uneventful perinatal period and when no obvious signs of cognitive deficits present themselves early in development. Early interventions such as training programs to strengthen executive functions might benefit some, but perhaps more important is information and support to families and to ensure a school environment where adjustments and compensations are made for possible cognitive weaknesses. In the face of cognitive deficits, such early support might allow for optimal development and prevent the child from falling further behind.

The strengths of this study include its longitudinal design and the fact that this cohort of children has had almost universal access to high-quality maternal and neonatal care in Sweden, enabling the study of the effects of preterm birth per se. The limitation is primarily the sample size, which limits the power of the statistical analyses. Some associations with background variables, as well as longitudinal streams involving smaller clusters that did not reach statistical significance, might do so in a larger study. Also, the results are conditioned on the tests and indices used, and we cannot exclude that other measures would have presented a slightly different picture. We have not investigated the link between cluster membership and brain-imaging data, which might reveal associations in brain volume or connectivity to performance level and profiles. Such analyses could give insight into the neural basis of the different patterns and might provide important information on essential mechanisms at play for those in our study whose development was uncertain at age 5½ years, that is, primarily individuals in Cluster 5C.

The aim of this study was to investigate the long-term stability in cognitive outcome patterns and developmental trajectories of subgroups of individuals born preterm. The person-oriented approach enabled us to look at the variability in individual cognitive profiles and development and thus to ascertain that individual outcome patterns are not disguised by group outcomes. We conclude that there are subgroups among the preterm born with distinct cognitive profiles. Cognitive ability at age 5½ years is highly predictive of ability at age 18: Individuals performing at low levels

at age 5½ years are unlikely to improve over time; and individuals who are performing at norm are unlikely to fall far below norm over time. However, compared to variable-based analyses, our study also shows that there are improvements in performance among a group of individuals performing at or above the norm at 5½ years that is offset by a slight lowering in performance among those in lower performing clusters. Also, the presumed relative strength in verbal ability among preterm born seems to be true for those who generally function within the normal range, whereas individuals with more pronounced deficits do not show this verbal advantage. Importantly, the present study shows that executive dysfunction is by no means characteristic for all individuals born preterm. The cognitive outcome after preterm birth in late adolescence is diverse, with a large group being seemingly unaffected by the bumpy start while others exhibit specific or general cognitive deficits. Already at late preschool age, however, developmental trajectories are fairly well established. Therefore, developmental monitoring and identification of those at risk for a less favorable outcome is imperative. Interventions could thus be initiated early, prior to school entry, to limit the effects that even mild cognitive deficits may have on learning and overall development.

Original manuscript received May 2, 2014
Revised manuscript accepted August 20, 2014
First published online October 3, 2014

REFERENCES

Allin, M., Walshe, M., Fern, A., Nosarti, C., Cuddy, M., Rifkin, L., … Wyatt, J. (2008). Cognitive maturation in preterm and term born adolescents. *Journal of Neurology, Neurosurgery, and Psychiatry*, *79*(4), 381–386. doi:10.1136/jnnp.2006.110858

Anderson, P. J., & Doyle, L. W. (2004). Executive functioning in school-aged children who were born very preterm or with extremely low birth weight in the 1990s. *Pediatrics*, *114*(1), 50–57. doi:10.1542/peds.114.1.50

Anderson, P. J., Howard, K., & Doyle, L. W. (2010). Executive function development in preterm children. In C. Nosarti, R. M. Murray, & M. Hack (Eds.), *Neurodevelopmental outcomes of preterm birth. From childhood to adult life* (pp. 195–208). Cambridge: Cambridge University Press.

Anderson, V., Jacobs, R., & Anderson, P. J. (2008). *Executive functions and the Frontal lobes: A lifespan perspective*. New York, NY: Psychology Press.

Arthur, G. A. (1947). *Point scale of performance tests. Revised form II*. New York, NY: The Psychological Corporation.

Aylward, G. P. (2005). Neurodevelopmental outcomes of infants born prematurely. *Journal of Developmental and Behavioral Pediatrics*, *26*(6), 427–440. doi:10.1097/00004703-200512000-00008

Aylward, G. P. (2010). Methodological considerations in neurodevelopmental outcome studies of infants born prematurely. In C. Nosarti, R. M. Murray, & M. Hack (Eds.), *Neurodevelopmental outcomes of preterm birth. From childhood to adult life* (pp. 164–175). Cambridge: Cambridge University Press.

Bergman, L. R., & El-Khouri, B. M. (2002). *SLEIPNER - A statistical package for person-oriented analyses*. Stockholm: Department of Psychology, Stockholm University.

Bergman, L. R., Magnusson, D., & El-Khouri, B. M. (2003). *Studying individual development in an interindividual context. A person-oriented approach*. Mahwah, NJ: Lawrence Eribaum Associates.

Bhutta, A. T., Cleves, M. A., Casey, P. H., Cradock, M. M., & Anand, K. J. S. (2002). Cognitive and behavioral outcomes of school-aged children who were born preterm. *JAMA*, *288*(6), 728–737. doi:10.1001/jama.288.6.728

Böhm, B., Katz-Salamon, M., Smedler, A.-C., Lagercrantz, H., & Forssberg, H. (2002). Developmental risks and protective factors for influencing cognitive outcome at 5½ years of age in very-low-birthweight children. *Developmental Medicine & Child Neurology*, *44*(8), 508–516. doi:10.1111/j.1469-8749.2002.tb00321.x

Böhm, B., Smedler, A.-C., & Forssberg, H. (2004). Impulse control, working memory and other executive functions in preterm children when starting school. *Acta Paediatrica*, *93*, 1363–1371. doi:10.1111/j.1651-2227.2004.tb02938.x

Delis, D. C., Kaplan, E., & Kramer, J. H. (2001). *Delis-Kaplan executive function system (D-KEFS). Examiner's manual*. San Antonio, TX: The Psychological Corporation.

Diamond, A. (2013). Executive functions. *Annual Review of Psychology*, *64*, 135–168. doi:10.1146/annurev-psych-113011-143750

Gäddlin, P.-O. (2011). Follow-up studies of very low birthweight children in Sweden. *Acta Paediatrica*, *100*(7), 940–949. doi:10.1111/j.1651-2227.2011.02288.x

Hack, M., Flannery, D. J., Schluchter, M., Cartar, L., Borawski, E., & Klein, N. (2002). Outcomes in young adulthood for very-low-birth-weight infants. *The New England Journal of Medicine*, *346*(3), 149–157. doi:10.1056/NEJMoa010856

Hack, M., Taylor, H. G., Drotar, D., Schluchter, M., Cartar, L., Wilson-Costello, D., & Morrow, M. (2005). Poor predictive validity of the Bayley Scales of Infant Development for cognitive function of extremely low birth weight children at school age. *Pediatrics*, *116*(2), 333–341. doi:10.1542/peds.2005–0173

Howard, K., Anderson, P. J., & Taylor, H. G. (2008). Executive functioning and attention in children born preterm. In V. Anderson, R. Jacobs, & P. J. Anderson (Eds.), *Executive functions and the frontal lobes. A lifespan perspective* (pp. 219–241). New York, NY: Psychology Press.

Ingemarsson, I. (2003). Gender aspects of preterm birth. *BJOG: An International Journal of Obstetrics and Gynaecology*, *110*(s20), 34–38. doi:10.1046/j.1471–0528.2003.00022.x

Johnson, S. (2007). Cognitive and behavioural outcomes following very preterm birth. *Seminars in Fetal & Neonatal Medicine*, *12*(5), 363–373. doi:10.1016/j.siny.2007.05.004

Koller, H., Lawson, K., Rose, S. A., Wallace, I., & McCarton, C. (1997). Patterns of cognitive development in very low birth weight children during the first six years of life. *Pediatrics*, *99*(3), 383–389. doi:10.1542/peds.99.3.383

Korkman, M. (1990). *NEPSY, neuropsychological assessment 4–7 years. Swedish version*. Stockholm: Psykologiförlaget AB.

Lindstam, R. (1968). *Griffiths mental development scales. Swedish version*. Stockholm: Skandinaviska testförlaget.

Lundequist, A. (2012). *Longitudinal studies of executive and cognitive development after preterm birth* (Doctoral Thesis). Stockholm University.

Lundequist, A., Böhm, B., & Smedler, A.-C. (2013). Individual neuropsychological profiles at age 5½ years in children born preterm in relation to medical risk factors. *Child neuropsychology*, *19*(3), 313–331. doi:10.1080/09297049.2011.653331

Luu, T. M., Ment, L. R., Allan, W., Schneider, K. C., & Vohr, B. R. (2011). Executive and memory function in adolescents born very preterm. *Pediatrics*, *127*(3), e639–e646. doi:10.1542/peds.2010-1421

Luu, T. M., Vohr, B. R., Allan, W., Schneider, K. C., & Ment, L. R. (2011). Evidence for catch-up in cognition and receptive vocabulary among adolescents born very preterm. *Pediatrics*, *128*(2), 313–322. doi:10.1542/peds.2010-2655

Magnusson, D., & Torestad, B. (1993). A holistic view of personality: A model revisited. *Annual Review of Psychology*, *44*, 427–452. 10.1146/annurev.ps.44.020193.002235

Miyake, A., & Friedman, N. P. (2012). The nature and organization of individual differences in executive functions: Four general conclusions. *Current Directions in Psychological Science*, *21*(1), 8–14. doi:10.1177/0963721411429458

Miyake, A., Friedman, N. P., Emerson, M. J., Witzki, A. H., Howerter, A., & Wager, T. D. (2000). The unity and diversity of executive functions and their contributions to complex "Frontal Lobe" tasks: A latent variable analysis. *Cognitive Psychology*, *41*(1), 49–100. doi:10.1006/cogp.1999.0734

Niklasson, A., & Albertsson-Wikland, K. (2008). Continuous growth reference from 24th week of gestation to 24 months by gender. *BMC Pediatrics*, *8*, 8. doi:10.1186/1471-2431-8-8

Perlman, J. M. (2001). Neurobehavioral deficits in premature graduates of intensive care—potential medical and neonatal environmental risk factors. *Pediatrics*, *108*(6), 1339–1348. doi:10.1542/peds.108.6.1339

Raz, S., Debastos, A. K., Newman, J. B., & Batton, D. (2012). Intrauterine growth and neuropsychological performance in very low birth weight preschoolers. *Journal of the International Neuropsychological Society*, *18*(02), 200–211. doi:10.1017/S1355617711001767

Roze, E., Van Braeckel, K. N. J. A., van der Veere, C. N., Maathuis, C. G. B., Martijn, A., & Bos, A. F. (2009). Functional outcome at school age of preterm infants with periventricular hemorrhagic infarction. *Pediatrics*, *123*(6), 1493–1500. doi:10.1542/peds.2008-1919

Saavalainen, P., Luoma, L., Bowler, D., Määttä, S., Kiviniemi, V., Laukkanen, E., & Herrgård, E. (2007). Spatial span in very prematurely born adolescents. *Developmental Neuropsychology*, *32*, 769–785. doi:10.1080/87565640701539535

Taylor, H. G., Minich, N., Bangert, B., Filipek, P. A., & Hack, M. (2004). Long-term neuropsychological outcomes of very low birth weight: Associations with early risks for periventricular brain insults. *Journal of the International Neuropsychological Society*, *10*(07), 987–1004. doi:10.1017/S1355617704107078

Taylor, L. C., Clayton, J. D., & Rowley, S. J. (2004). Academic socialization: Understanding parental influences on children's school-related development in the early years. *Review of General Psychology*, *8*(3), 163–178. doi:10.1037/1089-2680.8.3.163

Vohr, B. R., Wright, L. L., Dusick, A. M., Mele, L., Verter, J., Steichen, J. J., … Kaplan, M. D. (2000). Neurodevelopmental and functional outcomes of extremely low birth weight infants in the National Institute of Child Health and Human Development Neonatal Research Network, 1993–1994. *Pediatrics*, *105*(6), 1216–1226. doi:10.1542/peds.105.6.1216

Volpe, J. J., Kinney, H. C., Jensen, F. E., & Rosenberg, P. A. (2011). The developing oligodendrocyte: Key cellular target in brain injury in the premature infant. *International Journal of Developmental Neuroscience*, *29*(6), 565–582. doi:10.1016/j.ijdevneu.2011.07.008

Wechsler, D. (1991). *Wechsler intelligence scale for children: WISC-III. Swedish version*. Stockholm: Psykologiförlaget AB.

Wechsler, D. (1999). *Wechsler preschool and primary scale of intelligence - revised. Swedish version*. Stockholm: Psykologiförlaget AB.

Wechsler, D., Nyman, H., Johansson, C., Bragesjö, M., Bothén, P., Granath, K., & Waaler, E. (2004). *Wechsler adult intelligence scales - III NI. Swedish version for neuropsychological assessment*. Stockholm: Pearson Education Ltd.

Wilson-Ching, M., Molloy, C. S., Anderson, V. A., Burnett, A., Roberts, G., Cheong, J. L. Y., … Anderson, P. J. (2013). Attention difficulties in a contemporary geographic cohort of adolescents born extremely preterm/extremely low birth weight. *Journal of the International Neuropsychological Society*, *19*(10), 1097–1108. doi:10.1017/S1355617713001057

Woodward, L. J., Edgin, J. O., Thompson, D., & Inder, T. E. (2005). Object working memory deficits predicted by early brain injury and development in the preterm infant. *Brain*, *128*(11), 2578–2587. doi:10.1093/brain/awh618
